# Epithelial-mesenchymal interaction protects normal colonocytes from 4-HNE-induced phenotypic transformation

**DOI:** 10.1371/journal.pone.0302932

**Published:** 2024-04-26

**Authors:** Jacques Dupuy, Emma Cogo, Edwin Fouché, Françoise Guéraud, Fabrice Pierre, Pascale Plaisancié

**Affiliations:** Toxalim UMR1331 (Research Centre in Food Toxicology), Toulouse University, INRAE, ENVT, INP-Purpan, UPS, Toulouse, France; University of Rijeka Faculty of Medicine: Sveuciliste u Rijeci Medicinski fakultet, CROATIA

## Abstract

**Introduction:**

Recent studies have shown that epithelial-stromal interactions could play a role in the development of colorectal cancer. Here, we investigated the role of fibroblasts in the transformation of normal colonocytes induced by 4-HNE.

**Methods:**

Normal Co colonocytes and nF fibroblasts from the same mouse colon were exposed, in monoculture (m) or coculture (c), to 4-HNE (5 μM) twice weekly for 3 weeks. Gene expression was then analysed and the ability of Co colonocytes to grow in anchorage-independent conditions was tested in soft agar. Fibroblasts previously treated or not with 4-HNE were also seeded in culture inserts positioned above the agar layers to allow paracrine exchanges with colonocytes.

**Results:**

First, 60% of the genes studied were modulated by coculture in Co colonocytes, with notably increased expression of BMP receptors. Furthermore, while 4-HNE increased the ability of monoculture-treated Co colonocytes to form colonies, this effect was not observed in coculture-treated Co colonocytes. Adding a selective BMPR1 inhibitor during the treatment phase abolished the protective effect of coculture. Conversely, addition of a BMP4 agonist to the medium of monoculture-treated Co colonocytes prevented phenotypic transformation by 4-HNE. Second, the presence of nF(m)-HNE fibroblasts during the soft agar assay increased the number and size of Co(m) colonocyte colonies, regardless of whether these cells had been previously treated with 4-HNE in monoculture. For soft agar assays performed with nF(c) and Co(c) cells initially treated in coculture, only the reassociation between Co(c)-HNE and nF(c)-HNE resulted in a small increase in the number of colonies.

**Conclusions:**

During the exposure phase, the epithelial-mesenchymal interaction protected colonocytes from 4-HNE-induced phenotypic transformation via activation of the BMP pathway. This intercellular dialogue also limited the ability of fibroblasts to subsequently promote colonocyte-anchorage-independent growth. In contrast, fibroblasts pre-exposed to 4-HNE in monoculture strongly increased the ability of Co(m) colonocytes to form colonies.

## 1 Introduction

Colorectal cancer (CRC) is one of the leading causes of cancer death in men and women in developed countries with aging populations. According to recent statistics, there were 1.9 million new cases worldwide in 2022 [1]. Moreover, CRC incidence and mortality rates are steadily increasing in adults aged less than 50 years in the USA and Europe, with the median age of diagnosis changing from 72 years in 2001–2002 to 66 years in 2015–2016 [2].

Like many human cancers, sporadic CRC develops partly because of exposure to avoidable risk factors, including alcohol consumption, inactivity, overweight status and Western dietary patterns. A Western diet rich in heme proteins and fat notably increases the risk of epithelial intestinal cell exposure to polyunsaturated fatty acid peroxidation products, such as malondialdehyde, 4-hydroxy-2-hexenal (4-HHE) and 4-hydroxy-2-nonenal (4-HNE) [3–5]. These aldehydes present in our diet or produced during digestion add to those generated at the cellular level by oxidative stress and accumulate in tissues with age, all of which contribute to cellular dysfunction [6]. For example, 25-month-old Fischer 344 rats had 2 to 3 times more 4-HNE-protein adducts in their serum than did 7-month-old rats, while the concentration of free 4-HNE increased from approximately 0.3 μM in 7-month-old rats to 0.7 μM in 25-month-old rats [7]. It should also be noted that these molecules are cytotoxic and highly mutagenic, function as deregulators of various intracellular molecular pathways and are implicated in a large number of pathologies, such as metabolic diseases, neurodegenerative diseases and cancers [8–12]. Several studies have demonstrated that 4-HHE and 4-HNE resulting from the peroxidation of dietary lipids during gastrointestinal transit promote colorectal carcinogenesis [3, 5]. γH2AX labelling assays also revealed that 4-HHE and 4-HNE are genotoxic to normal colonocytes [3, 13] and may lead to the initiation and progression of carcinogenesis. This hypothesis was confirmed by Wang et al., who showed that exposure to a weekly dose of 4-HNE (1 μM) for 10 weeks induced the transformation of immortalized normal mouse colonocytes and that allografts from these cells resulted in the formation of poorly differentiated carcinomas [14].

Because of the cellular alterations they induce, these reactive aldehydes could play an important role in the initiation and promotion of CRC not only by acting on epithelial cells but also on subepithelial fibroblasts. Indeed, 4-HNE has been shown to promote the transition of fibroblasts toward an activated phenotype [11], the latter being known to play a major role in various intestinal pathologies. Recent research has thus introduced the notion of a mesenchymal microenvironment or tumor ecosystem and shown that the development of CRC can be explained not only by the progressive accumulation of mutations and epigenetic modifications within the nucleus of colonocytes but also by the interactions of these cells with the mesenchyme [15]. Among mesenchymal cell populations, subepithelial fibroblasts play a central role in dialogue with the epithelium due to their proximity to colonocytes and their role in regulating the proliferation and differentiation of the epithelial cells they surround [16, 17]. When homeostasis is disrupted, fibroblasts acquire an activated form. These cells are called activated fibroblasts and, in the case of a tumoral environment, cancer-associated fibroblasts (CAFs). While resting fibroblasts are thought to inhibit tumor initiation and progression, most CAFs support tumor development, notably by increasing epithelial cell proliferation and survival, secreting numerous proinflammatory cytokines and actively participating in extracellular matrix remodeling [18, 19]. For example, subcutaneous injection of CAFs with HCT116 human colon cancer cells clearly promoted tumor growth in mice compared with injection of HCT116 cells alone [20]. An experiment carried out on severe combined immunodeficiency (SCID) mice also showed that injection of colon cancer stem cells with CAFs into the graft improved tumor uptake [21].

In this context, our aim was to investigate the effect of a fibroblastic microenvironment on the transformation of normal colonocytes induced by repeated exposure to a noncytotoxic concentration of 4-HNE, which was chosen as a "model" molecule for the study of lipid peroxidation products. The concentration of 4-HNE used (5 μM) was within the same order of magnitude as that detected in intestinal contents [5]. To carry out our study, we used a previously validated coculture model to explore the epithelial–mesenchymal interactions of healthy colons [22]. This model involves conditionally immortalized normal colonocytes and conditionally immortalized normal fibroblasts from the same mouse colon, making it a very valuable tool for studying the role of epithelial–mesenchymal interactions in the early phases of colonocyte transformation during tumorigenesis. Repeated exposure to 4-HNE was followed by a soft agar colony formation assay. This test measures the anchorage-independent growth ability of cells, a characteristic of cell transformation during tumorigenesis [23].

## 2 Materials and methods

### 2.1 Cell culture

Conditionally immortalized normal colonocytes (Co) and fibroblasts (nF) derived from the same mouse colon were established as previously described [22, 24, 25]. Both cell lines express the simian virus 40 large tumor antigen thermolabile gene (AgT tsA58) under the control of interferon γ (IFN-γ). The cells were grown at a permissive temperature of 33°C in DMEM supplemented with 10% (v/v) fetal calf serum, 1% (v/v) penicillin/streptomycin, 10 units/mL IFN-γ and 10 U/ml epidermal growth factor (EGF) [22]. The two cell lines did not have mutations in *Apc*, *Tp53*, *Kras*, *Smad4* or *Braf*. The genome sequence of the cells is accessible through the following link. https://www.ncbi.nlm.nih.gov/sra/PRJNA800346.

### 2.2 Experimental design and 4-HNE exposure

**Fig 1:**
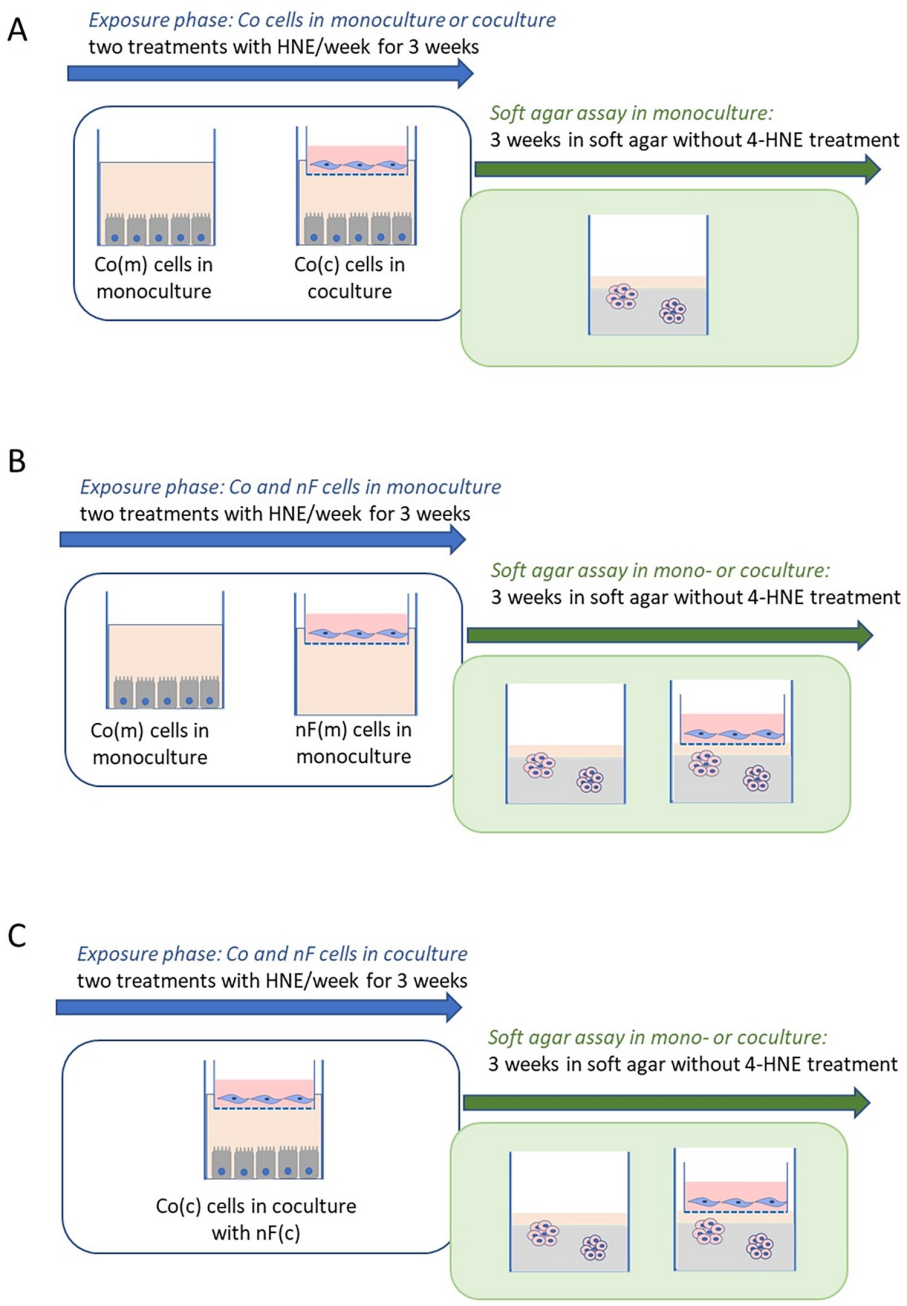
Schematic representation of the 4-HNE treatment and soft agar culture protocols. Normal mouse colonocytes in monoculture (m) or in coculture (c) with nF fibroblasts were exposed to a noncytotoxic concentration of 4-HNE (5 μM, 1 h) twice weekly during the proliferative phase. After 3 weeks of repeated 4-HNE treatment, we assessed the expression of various genes in Co and nF cells in monoculture and coculture using RT‒qPCR and performed a soft agar colony formation assay, which is recognized for its ability to assess cell transformation. Cells were not exposed to 4-HNE during the soft agar assay. **A/** Co cells in monoculture or coculture with nF fibroblasts during the treatment phase. A soft agar assay was performed in the absence of fibroblasts and without 4-HNE treatment. **B/** Co and nF cells in monoculture were exposed to 4-HNE during the treatment phase. The soft agar assay was performed in the absence or presence of previously treated fibroblasts. The latter were seeded in culture inserts placed on top of the agar wells. **C/** Co and nF cells in coculture were exposed to 4-HNE during the treatment phase. The soft agar assay was performed in the absence or presence of previously treated fibroblasts. The latter were seeded in culture inserts placed on top of the agar wells.

Co(m) and nF(m) cells correspond to cells treated in monoculture, while Co(c) and nF(c) cells refer to those treated in coculture (Fig 1). 4-HNE was synthesized as previously described [26] and was used at a noncytotoxic concentration of 5 μM (S1 Fig). The cell treatment phase lasted 3 weeks. On Monday of the first week, Co cells were seeded in 6-well plates (Sarstedt, Numbrecht, Germany) at a density of 4.10^5^ cells/well. The nF fibroblasts were seeded onto Thincert™-Tissue Culture Inserts in six-well plates with a 0.4 μm pore size (Greiner bio-One France, Courtaboeuf, France) at a density of 3.10^4^ cells/insert and cultured in the presence or absence of colonocytes on the bottom of the well. We chose to seed epithelial cells in 6-well plates rather than on Thincert™-Tissue Culture inserts, as epithelial cells are very difficult to trypsinize in these inserts, leading to cell selection over time and hence experimental bias. Co and nF cells were exposed to 4-HNE (5 μM, 1 h) in serum-free medium on Tuesday between 11:00 and 12:00. The control wells received serum-free medium alone. The cells were then trypsinized on Thursday morning, counted (two counts per well with Luna^TM^ cell counter), centrifuged and reseeded at the previously indicated density. The cells were exposed again to 4-HNE or to serum-free medium on Friday. The procedure was continued for 3 weeks (Fig 1). After the trypsin steps, the unused cells were centrifuged, and the pellets were stored for western blotting. The experiments were repeated at least four times in triplicate.

### 2.3 Treatment with the bone morphogenetic protein (BMP) signaling agonist SB4 or prostaglandin E2 (PGE_2_)

For experiments with SB4 (2-[[(4-bromophenyl)methyl]thio]benzoxazole; Sigma Aldrich), Co(m) cells were subjected to the same protocol as described above but in the presence of either the SB4 agonist (0.1–1 μM) or an equal volume of dimethyl sulfoxide (DMSO) vehicle. The SB4 and its vehicle were renewed every day and added during treatment with 4-HNE in serum-free medium. The experiments were repeated at least four times in triplicate. Experiments with PGE_2_ (1 μM, Sigma Aldrich) were carried out using an identical protocol.

### 2.4 Coculture with K02288, a BMP type I receptor inhibitor, or PGE_2_

For experiments with K02288 (Sigma Aldrich), cocultured Co(c) and nF(c) cells were subjected to the same protocol as described in the "Exposure to 4-HNE" section but in the presence of either K02288 (500 nM) or an equal volume of DMSO vehicle. K02288 and its vehicle were renewed every day and added during treatment with 4-HNE in serum-free medium. The experiments were repeated at least four times in triplicate. Experiments with PGE_2_ (1 μM) were carried out using an identical protocol.

### 2.5 Cell count assay using a Luna^TM^ cell counter

After each trypsin step, nF fibroblasts and Co colonocytes suspended in culture medium were counted (in duplicate) with a LUNA^TM^ (LogoS Biosystems, Villeneuve d’Ascq, France) using the “Bright-Field Counting mode” as described in the manual. Before the cells were counted, the upper and lower gates of the counter were adjusted manually to eliminate small particles.

### 2.6 Soft agar colony formation assay

To establish the soft agar assay, we used the protocol described by Du et al. [23] and Borowicz et al. [27], which we adapted for use with our cell lines. Briefly, agar solutions (1.4% and 2%) prepared in distilled water were autoclaved and stored at 4°C before use. A few days before the start of the assay, 6-well plates were prepared by dispensing 1 ml of a preheated 0.5% agar solution (bottom agar layer) into each well. To do this, 2 ml of 2% agar solution (42°C) was added to 6 ml of complete medium prewarmed to 37°C in a water bath. The plate was then left at room temperature for 1 h to allow the bottom agar layer to solidify before being stored at 4°C until use. On the day of preparation on the top agar layer, the Co cells were trypsinized, suspended at a density of 1.333 × 10^3^ cells/ml in complete medium and maintained in a water bath at 37°C. The top layer of agar was prepared by mixing 3 ml of 1.4% agar solution (at approximately 42°C) with 9 ml of diluted cell suspension to obtain a final cell concentration of 10^3^ cells/ml. One milliliter of this mixture was then rapidly dispensed into each well, producing a top layer of 0.35% agar medium. The plate was gently shaken by hand and held at room temperature for 1 h. Each treatment condition was tested in triplicate. Before transferring the plates to the incubator, 0.5 ml of complete medium was added to each well. If needed, nF(m) and nF(c) fibroblasts were trypsinized, counted and seeded onto Thincert™ tissue culture inserts in 6-well plates at a density of 3.10^4^ fibroblasts/insert to be placed on top of the agar wells. nF(m) fibroblasts were reassociated with Co(m) colonocytes, and nF(c) fibroblasts were reassociated with Co(c) colonocytes. The cells were seeded in soft agar and cultured for three weeks. The culture medium was refreshed every week.

### 2.7 Colonies counting

After 3 weeks of culture on soft agar, the wells were stained with 0.1% crystal violet solution (diluted in water) for 10 minutes at room temperature and then washed 3 times with PBS. After the last wash, 0.5 mL of PBS was again added to each well to prevent the agar from drying. The 6-well plates were sealed with parafilm and stored at 4°C before counting. The colonies observed were classified into three categories: small colonies (with diameters between 25 and 50 μm), medium colonies (with diameters between 50 and 100 μm) and large colonies (with diameters greater than 100 μm). For each test, three wells per condition were analysed. In addition, all the experiments were repeated a minimum of four times. The results are presented as the mean (± SEM) colony number per well (1000 seeded cells in soft agar).

### 2.8 RT-qPCR

Total RNA was isolated from Co and nF cells derived from the last trypsin step using the MiniRNeasy Kit (Qiagen, Hilden, Germany). RNA samples (1 μg) were then reverse-transcribed with the iScript™ Reverse Transcription Supermix (Bio-Rad) for real-time quantitative polymerase chain reaction (qPCR) analyses. The primers used for the SYBR Green assays are listed in S1 Table. Amplifications were performed on a ViiA 7 Real-Time PCR System (Applied Biosystems). The qPCR data were normalized to the level of hypoxanthine-guanine phosphoribosyltransferase (Hprt1) messenger RNA (mRNA) and analysed via LinRegPCR v.11 software. The data are expressed as the mean ± SEM (n = 3 assays in triplicate).

### 2.9 Western blot

Cell proteins were extracted in RIPA lysis buffer containing a cocktail of protease and phosphase inhibitors, as previously described [22]. Total proteins (20 μg per lane) were resolved via 10% SDS‒PAGE, transferred to a nitrocellulose membrane and incubated with primary antibodies against Cox2, cytokeratin 18 (Krt18), αSMA, Rad51, p21, Hsc70 or vinculin (S2 Table). All of the samples were incubated at 4°C overnight. Bound antibodies were detected by using the WesternBreeze Immunodetection Chemiluminescent System (Invitrogen, Fisher Scientific, Illkirch, France). The optical density of the bands was visualized with an imaging system (ChemiDoc, Bio-Rad), analysed with Quantity One-Image Analysis software (Bio-Rad Laboratories) and normalized with an internal control (HSC70 or vinculin).

### 2.10 Immunofluorescence (IF) labelling and analysis

At the end of the treatment phase, the different cell populations were trypsinized. Some cells were used for immunofluorescence assays on slides. To this end, Co or nF cells (2x10^4^ cells/well) were seeded on an 8-well slide (Millicell® slide, Merck) and allowed to incubate for 24 h. Afterwards, the cells were fixed with ice-cold 100% methanol (10 min) and washed with ice-cold PBS. Preparations were then blocked with 2.5% normal horse serum for 20 min. The primary antibody (Ab) (S2 Table) diluted with Animal-Free Blocker® and Diluent (Vector Laboratories, Burlingame, CA, United States) was then applied at room temperature for 2 h. After the PBS baths, slides were incubated for 15 min with Amplifier Antibody (VectaFluor™ Excel Amplified Kit, Vector Laboratories) and then with VectaFluor Reagent (30 min, DyLight® 594, Vector Laboratories). The cell preparations were counterstained with DAPI (Sigma Aldrich) for 1 min, and mounted. The specificity of the primary Ab was checked in negative controls in which only the primary Ab was omitted. Images were acquired (Leica Microsystems fluorescence microscope Leica DM2000 Ledand Leica DFC7000T camera using a 40x objective) and processed using Leica Advanced Application Suite software (version 2018.1, Leica Microsystems: Wetzlar, DEU). Five different zones in each well of the culture chamber were analysed (with an average of one hundred cells per zone), and an average value was calculated for each cell passage. The experiments were replicated a minimum of three times.

### 2.11 Statistical analysis

The data are presented as the mean ± SEM. The numbers of colonocytes were analysed with a t test. The data from the experiments with the SB4 agonist, the BMP type I receptor inhibitor K02288 and PGE_2_ were subjected to analysis of variance (ANOVA) followed by Tukey’s multiple comparisons test. For other data, two-way ANOVA was performed to assess the effects of 4-HNE treatment and the presence of fibroblasts as well as the interaction between these two factors. Multiple comparisons were performed with Tukey’s post hoc test. The significance level was set at *p*  =  0.05 (GraphPad Prism 10.0.3 software).

## 3 Results

### 3.1 Evolution of murine colonocytes and fibroblasts during repeated exposure to 4-HNE

#### Changes in cell numbers over time

**Fig 2:**
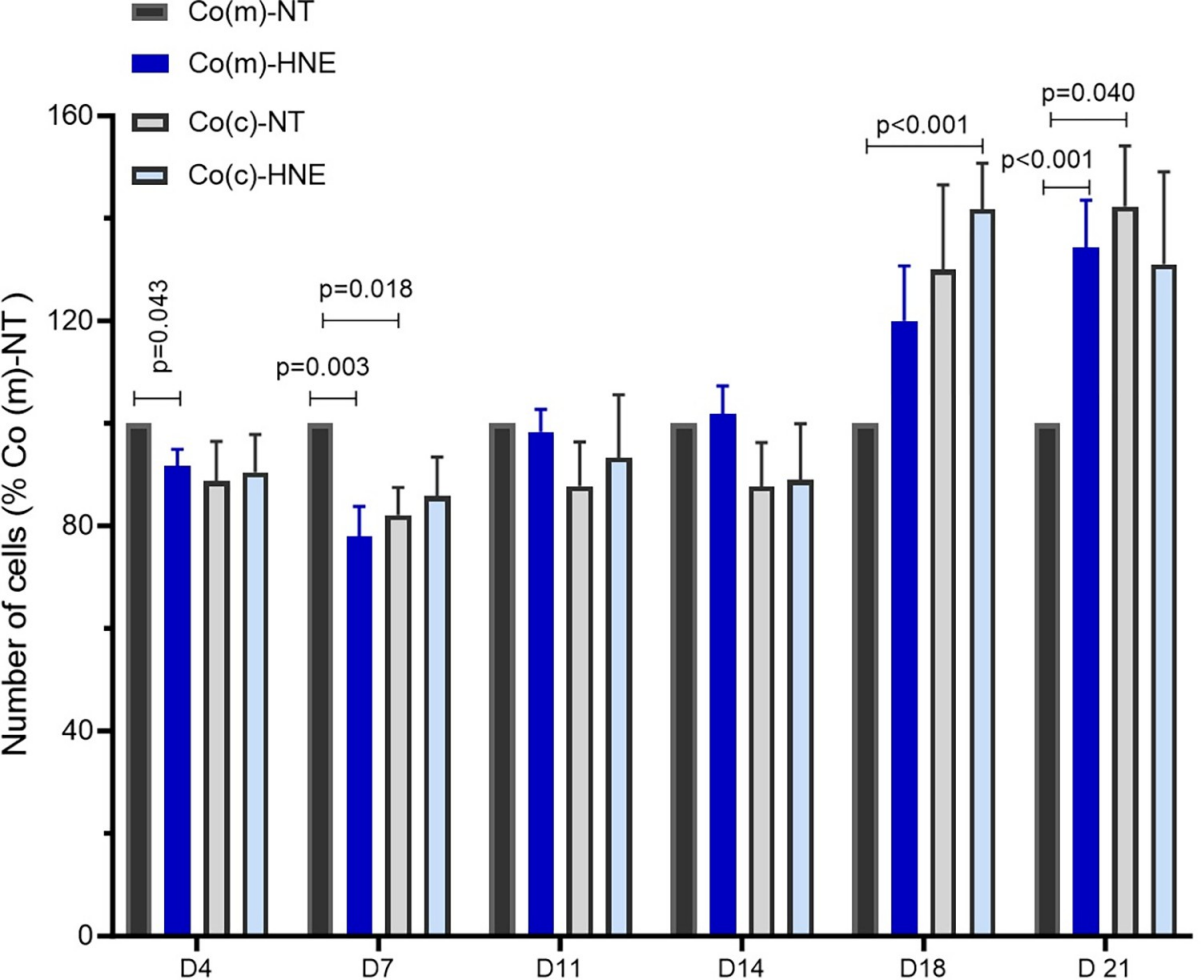
Changes in the number of colonocytes treated with 4-HNE in monoculture or coculture over time. On Monday of the first week, Co cells were seeded in 6-well plates in monoculture (m) or in coculture (c) with nF fibroblasts seeded on Thincert™ culture inserts (0.4 μm pores). Cells were exposed to 4-HNE (5 μM, 1 h) in SVF-free medium on the Tuesday morning. The control wells (NT) received SVF-free medium only. Co and nF-cultured cells were trypsinized on the Thursday Morning, counted, centrifuged and reseeded. The cells were treated again on Friday mornings with 4-HNE (5 μM, 1 h). In addition, so on for 3 weeks. The results were normalized to the number of Co(m)-NT cells. The data are expressed as the mean ± SEM of 8 experiments. A t-test was performed to compare the Co(m)-NT group with the other groups.

For Co(m) cells in monoculture, three phases could be observed (Fig 2). First, compared with no treatment, 4-HNE reduced the number of Co(m) cells obtained (week 1). This was followed by a second phase (week 2), during which no difference was observed between the number of Co(m)-NT and Co(m)-HNE cells. The third phase was characterized by greater growth of Co(m)-HNE cells than of Co(m)-NT cells (p<0.05). Note that a single treatment with 4-HNE at a concentration of 5 μM was sufficient to induce γH2AX in Co(m) cells, whereas no significant effect was observed in nF(m) cells, even when 4-HNE was tested at a concentration of 10 μM (S1 Fig).

For Co(c) cells cocultured with nF fibroblasts, a significant reduction in the growth of Co(c)-NT cells was observed compared to that of Co(m)-NT cells after 7 days. This result is in agreement with our previous study showing that the interaction between Co colonocytes and nF fibroblasts results in decreased Co cell growth [22]. This phenomenon was reversed during the 3rd week, with Co(c)-NT growing faster than Co(m)-NT cells. Under coculture conditions, 4-HNE did not alter the growth of treated Co(c) cells compared with Co(c)-NT control cells, at any time during the 3-week protocol.

#### Protein expression in Co and nF cells at D7 and D21

The Cox2/PGE_2_ pathway is involved in the various stages of colon carcinogenesis, and we were interested in the protein expression of this enzyme. Western blot analysis of Cox2 protein levels in Co cells at D21 revealed that the interaction between the cell culture protocol and 4-HNE treatment was significant (Table 1). The cell culture protocol (monoculture or coculture) also had a significant effect (P<0.001). Cox2 expression was greater in Co(m)-HNE cells than in Co(m)-NT cells, Co(c)-NT and Co(c)-HNE colonocytes (Table 1). IF labelling of Cox2 in colonocytes 24 hours after the last trypsin step confirmed this result (S2 Fig). This increase in Cox2 expression was not observed on day 7 (Table 1).

**Table 1:** Western blot analysis of Cox2 in Co and nF cells exposed to 4-HNE.

COX2	D7 Mean ±SEM	Two way Anova	D21 Mean ±SEM	Two way Anova
Co(m)-NT	100.0 ± 7.8	HNE treatment NS Monoculture vs coculture NS Interaction NS	100.0 ± 6.5 ^b^	HNE treatment NS **Monoculture vs coculture p<0.001** **Interaction p = 0.045**
Co(m)-HNE	78.9 ± 12.7		**137.5 ± 7.8** ^**a**^	
Co(c)-NT	99.7 ± 18.2		86.3 ± 11.1 ^b^	
Co(c)-HNE	100.2 ± 14.6		88.3 ± 15.7 ^b^	
nF(m)-NT	100.0 ± 9.1	HNE treatment NS Monoculture vs coculture NS Interaction NS	100.0 ±6.3 ^b^	HNE treatment NS **Monoculture vs coculture p = 0.0012** **Interaction p = 0.0198**
nF(m)-HNE	102.8 ± 10.1		90.6 ± 3.9 ^b^	
nF(c)-NT	84.9 ± 10.5		108.1 ± 7.0 ^b^	
nF(c)-HNE	83.4 ± 5.9		**133.1 ± 8.7** ^**a**^	

Co and nF cells were exposed to 4-HNE in monoculture (m) or in coculture (c). Analyses were performed at D7 and D21 of the repeated stimulation protocol. The results were normalized and expressed as the mean ± SEM of at least 3 experiments. Two-way ANOVA was performed followed by Tukey’s multiple comparisons test. The same letters indicate no significant difference between the groups.

In the case of nF fibroblasts, the results from two-way ANOVA showed that the cell culture protocol modulated the protein level of Cox2 at D21 (Table 1). A significant interaction effect between the cell culture protocol and 4-HNE treatment was also observed. Tukey’s post hoc test indicated that the Cox2 protein level was significantly greater in nF(c)-HNE cells than in nF(c)-NT, nF(m)-NT and nF(m)-HNE cells (Table 1). IF staining showed that Cox2-positive nF(c)-HNE fibroblasts were more numerous than nF(c)-NT fibroblasts (S2 Fig).

For cell type-specific markers, the interaction effect between the cell culture protocol and 4-HNE treatment was significant for cytokeratin 18 protein levels in Co cells at D21 but not at D7 (S3 Table). The coculture protocol significantly decreased the number of nF fibroblasts positive for αSMA at D21 (p<0.001) (S3 Fig). A significant interaction effect between the culture protocol and 4-HNE treatment was also observed (S3 Fig). The percentage of nF fibroblasts positive for αSMA was greater for nF(m)-NT cells than for nF(m)-HNE, nF(c)-NT and nF(c)-HNE cells. Western blot and RT–qPCR data corroborated these results (S3 Table). However, it should be noted that treatment of nF cells in SVF-free medium for one hour twice weekly resulted in increased protein and gene expression of αSMA in nF(m)-NT cells compared with that in nF cells cultured in complete medium (S3 Fig). Finally, Vimentin protein expression was significantly reduced in fibroblasts by the coculture protocol at D21 (S3 Table).

### 3.2 Expression of several genes in Co and nF cells after three weeks of treatment with 4-HNE

#### General analysis

Surprisingly, only two of the forty-two genes studied (*Tnfrsf1b* and *Bmp2*) responded to 4-HNE treatment in Co cells, whereas a very large number of genes (25/42) were significantly modulated by the presence of fibroblasts in a coculture system (Table 2). A significant interaction effect between 4-HNE treatment and culture method was observed for the expression of *Bmp4*, *smad1*, *Tnfrsf1a* and *Cd44*. In nF fibroblasts, 4-HNE treatment modulated fourteen of the forty-two genes studied, and the presence of colonocytes in coculture modified the mRNA level of twelve of the forty-two genes considered (Table 3). In particular, 4-HNE treatment increased *Nrf2* expression and decreased *Bach1* expression in fibroblasts (S4 Fig). A significant interaction was noted for *Acvr2b*, *Rock1* and *Egfr* mRNA expression (Table 3).

**Table 2:** Results of two-way ANOVA for Co cells.

Gene symbol	4-HNE treatment	Cell culture method	Interaction
APC	ns	p = 0.019	ns
Ctnnb1	ns	p = 0.0012	ns
Wnt2b	ns	p<0.001	ns
Wnt5a	***ne***	***ne***	***ne***
Prom1	ns	ns	ns
Cd44	ns	p<0.001	p = 0.037
Notch1	ns	ns	ns
Notch2	ns	p = 0.014	ns
Hes1	ns	p = 0.010	ns
Klf4	ns	p = 0.019	ns
Krt18	ns	p = 0.0029	ns
Nfe2l2	ns	ns	ns
Bach1	ns	ns	ns
Keap1	ns	p<0.001	ns
Gpx4	ns	p = 0.049	ns
Smad2	ns	p<0.001	ns
Tgfb1	ns	p<0.001	ns
Il6	ns	ns	ns
Egfr	ns	ns	ns
Tnfα	ns	p = 0.0322	ns
Tnfrsf1a	ns	ns	p = 0.0398
Tnfrsf1b	p<0.001	ns	ns
c-Myc	ns	ns	ns
Ccnd1	ns	p = 0.011	ns
Rock1	ns	ns	ns
RhoA	ns	ns	ns
Akt	ns	ns	ns
Bmp4	ns	p = 0.007	p = 0.021
Bmp2	p = 0.041	p = 0.040	ns
Grem1	***ne***	***ne***	***ne***
Smad1	ne	p = 0.049	p = 0.0246
Bmpr1	ns	p = 0.0051	ns
Bmpr2	ns	p = 0.0014	ns
Acvr2a	ns	p = 0.0053	ns
Acvr2b	ns	ns	ns
Rad51	ns	p = 0.0056	ns
Pten	ns	p = 0.001	ns
Cdkn1a (p21)	ns	p<0.001	ns
Acta2	ns	p = 0.0034	ns
Vim	ns	ns	ns
Col1a1	ns	p<0.001	ns
Col1a2	***ne***	***ne***	***ne***
	**2/42 genes**	**25/42 genes**	**4/40 genes**

ne = not expressed; ns = not significant. Two-way ANOVA was used to evaluate the RT‒qPCR data. The statistical analysis was performed with GraphPad Prism 10.1. Red and blue indicate high and low expression levels, respectively.

**Table 3:** Results of two-way ANOVA for nF cells.

Gene symbol	4-HNE treatment	Cell culture method	Interaction
Apc	ns	ns	ns
Ctnnb1	ns	ns	ns
Wnt2b	p = 0.0070	ns	ns
Wnt5a	ns	p = 0.0376	ns
Prom1	p = 0.0282	p = 0.0045	ns
Cd44	ns	p = 0.0189	ns
Notch1	p = 0.0085	ns	ns
Notch2	p = 0.0047	ns	ns
Hes1	ns	ns	ns
Klf4	ns	ns	ns
Krt18	*ne*	*ne*	*ne*
Nfe2l2	p = 0.049	ns	ns
Bach1	p = 0.0028	ns	ns
Keap1	ns	ns	ns
Gpx4	ns	ns	ns
Smad2	ns	ns	ns
Tgfb1	ns	ns	ns
Il6	ns	p<0.001	ns
Egfr	p = 0.044	ns	p = 0.040
Tnfa	*ne*	*ne*	*ne*
Tnfrsf1a	ns	p<0.001	ns
Tnfrsf1b	p<0.001	ns	ns
c-Myc	ns	p = 0.0041	ns
Ccnd1	ns	ns	ns
Rock1	ns	ns	p = 0.016
RhoA	ns	ns	ns
Akt	p = 0.030	ns	ns
Bmp4	p = 0.0136	ns	ns
Bmp2	p = 0.0028	ns	ns
Grem1	ns	ns	ns
Smad1	ns	p<0.001	ns
Bmpr1	ns	p = 0.047	ns
Bmpr2	ns	p = 0.0051	ns
Acvr2a	ns	p = 0.016	ns
Acvr2b	ns	ns	p = 0.0125
Rad51	ns	p = 0.0011	ns
Pten	ns	p = 0.0262	ns
Cdkn1a (p21)	ns	ns	ns
Acta2	p = 0.0182	ns	ns
Vim	p = 0.011	ns	ns
Col1a1	ns	ns	ns
Col1a2	p<0.001	ns	ns
	14/42 genes	12/42 genes	3/42 genes

ne = not expressed; ns = not significant. Two-way ANOVA was used to evaluate the RT‒qPCR data. The statistical analysis was performed with GraphPad Prism 10.1. Red and blue indicate high and low expression levels, respectively.

### BMP pathway

**Fig 3:**
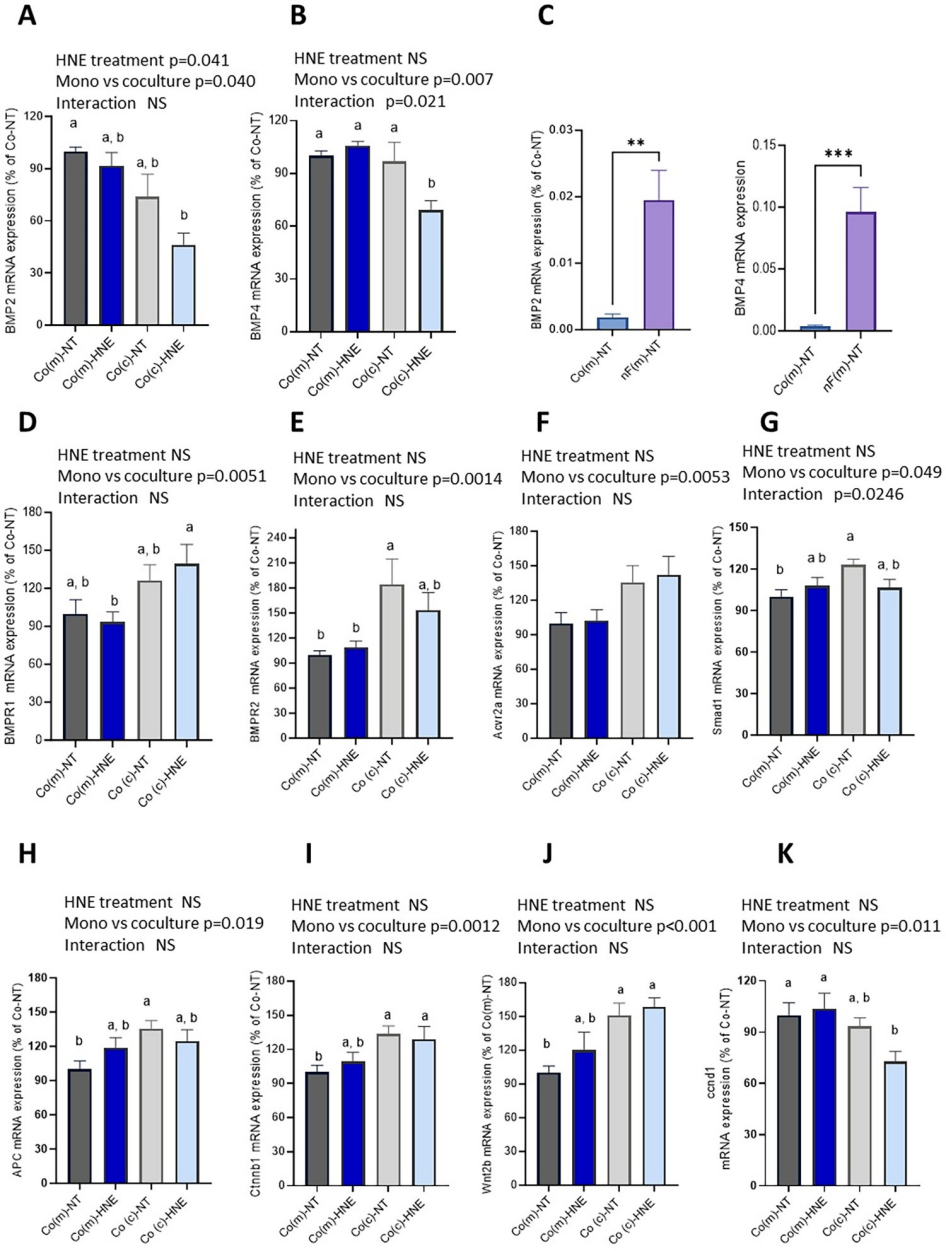
Expression of genes involved in the BMP and Wnt signaling pathways in Co cells (D21). Co cells exposed to 4-HNE in monoculture (m) or in coculture (c). The qPCR data were normalized to the level of hypoxanthine-guanine phosphoribosyltransferase (*Hprt1*) messenger RNA (mRNA) and analysed via LinRegPCR v.11 software. The data are presented as the mean ± SEM of 3 independent experiments (in triplicate) and are expressed as percentages relative to Co(m)-NT. Two-way ANOVA was performed, followed by Tukey’s multiple comparisons test. The same letters indicate no significant difference between the groups. **A-G**/ mRNA levels of BMP ligands and receptors in Co cells. **H-K/** mRNA levels of four genes implicated in the Wnt pathway (*Apc*, *Ctnb1*, *Wnt2b*, and *Ccnd1*).

We were also interested in the BMP signaling pathway, as it is strongly involved in the dialogue between subepithelial fibroblasts and colonocytes. Several genes involved in the BMP pathway were regulated in the two cell lines, either by 4-HNE treatment or by the culture protocol (six of the eight genes analysed for Co cells and nF cells) (Fig 3 and S5 Fig). In particular, the presence of fibroblasts increased the mRNA levels of BMP receptors (Bmpr1, Bmpr2, and Acvr2a) in Co cells (Fig 3). The Bmpr1 mRNA level in Co(c)-HNE cells was greater than that in Co(m)-HNE cells. In addition, Bmpr2 gene expression was greater in Co(c)-NT cells than in Co(m)-NT and Co(m)-HNE cells. A significant impact of the culture protocol on the mRNA level of the BMP ligands Bmp2 and Bmp4 was also observed in Co cells. The expression of these two ligands was lower in Co cells than in nF fibroblasts (ten and 24 times lower for Bmp2 and Bmp4, respectively) (Fig 3C). Finally, the expression of the intracellular effector Smad1 was modified by the culture protocol and was significantly greater in Co(c)-NT cells than in Co(m)-NT cells. The interaction effect between the cell culture protocol and 4-HNE treatment was also significant (Fig 3G). Regarding nF fibroblasts, Bmp2 and Bmp4 mRNA levels were significantly affected by 4-HNE treatment, whereas the presence of Co cells modified the expression of Bmpr1, Bmpr2 and Acvr2a (S5 Fig). In these cells, the Bmp4 expression level was greater than the Bmp2 expression level (S5 Fig). Interestingly, IF assays revealed that the cell culture protocol affected the percentage of Bmp4-positive nF fibroblasts (S6 Fig). We observed that nF(c)-NT fibroblasts had a higher percentage of Bmp4 positive cells than nF(m)-NT and nF(m)-HNE cells 24 hours after the last trypsin step. Dot blot assays also revealed the presence of Bmp2 in the supernatant of nF cells (S6 Fig).

### Other genes studied

**Fig 4:**
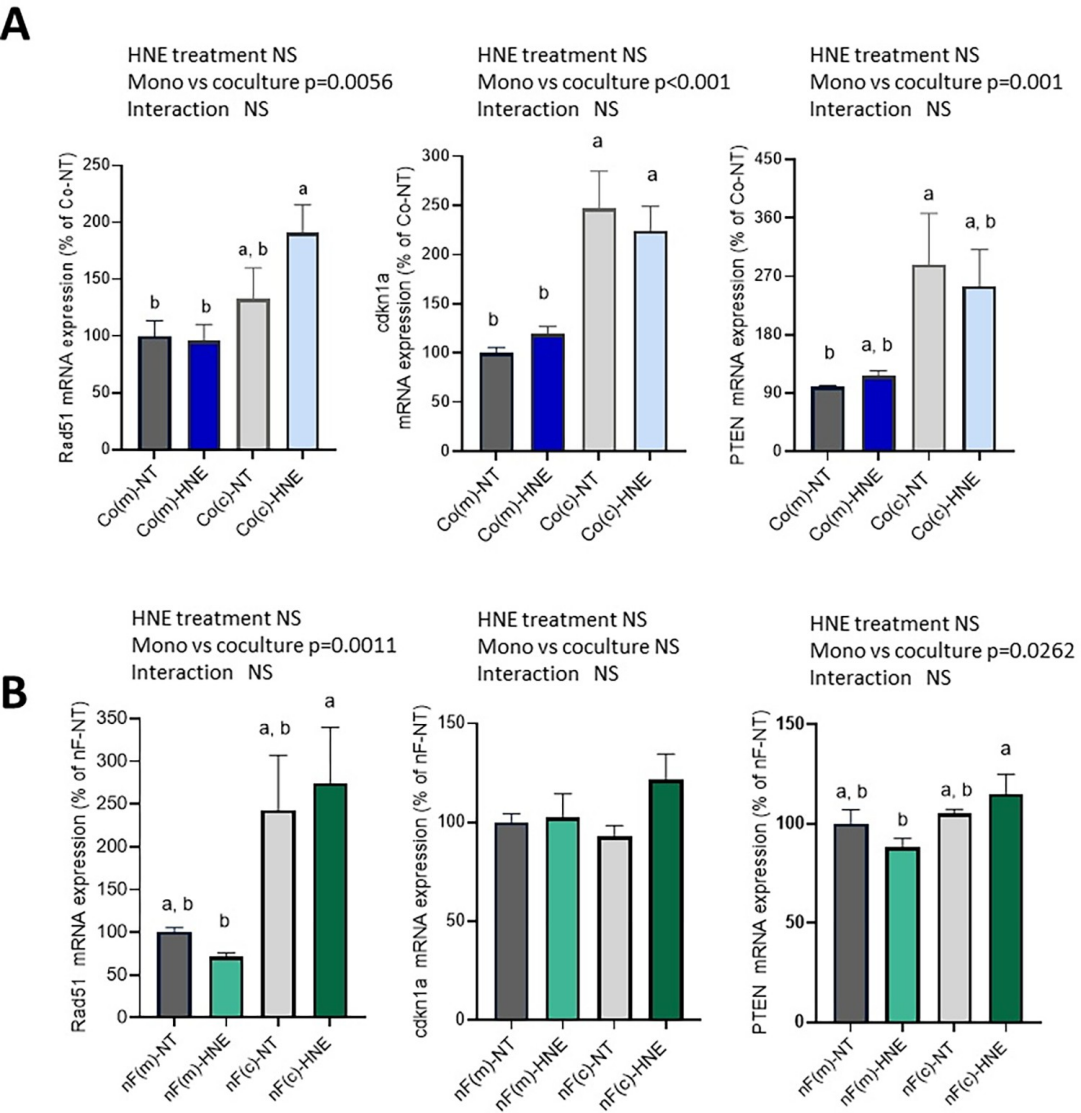
Expression of genes that can be considered DNA guardians in Co and nF cells. Relative mRNA expression of Rad51, Pten, and Cdkn1a in Co (A) and nF cells (B) after 3 weeks of repeated 4-HNE treatment. qPCR data were normalized to the Hprt1 mRNA level and analysed using LinRegPCR v.11 software. The data are presented as the mean ± SEM of 3 independent experiments (in triplicate) and are expressed as percentages relative to Co(m)-NT. Two-way ANOVA was performed followed by Tukey’s multiple comparisons test. The same letters indicate no significant difference between the groups.

In colonocytes, the expression of genes that can act as DNA guardians (Rad51, Pten, Cdkn1a) was increased by coculture with nF fibroblasts (Fig 4A). Tukey’s post hoc test showed that Rad51 and Cdkn1 mRNA levels were greater in Co(c)-HNE cells than in Co(m)-HNE cells. The Western blot data demonstrated that the coculture protocol significantly increased the protein levels of Rad51 and p21 in Co cells at both D7 and D21 (Table 4). In nF cells, Rad51 and Pten mRNA levels were also upregulated by coculture (Fig 4B). Furthermore, the expression of genes involved in the TNFα pathway (*Tnfα*, *Tnfrsf1a* and *Tnfrsf1b*) was modified in Co and nF cells by the culture protocol, by 4-HNE treatment or by the interaction between the two (S4 Table). The coculture protocol also induced an increase in the Il-6 mRNA level in nF cells (p<0.001) (S5 Table) and in the Tgfb1 mRNA level in Co cells (p<0.001).

**Table 4:** Western blot analysis of Rad51 and p21 in Co cells exposed to 4-HNE.

	Cell line	D7 Mean ±SEM	Two way Anova	D21 Mean ±SEM	Two way Anova
**Rad51**	Co(m)-NT	100.0 ± 8.4 ^**b**^	HNE treatment NS **Monoculture vs coculture p<0.001** Interaction NS	100.0 ± 4.8	HNE treatment NS **Monoculture vs coculture p = 0.022** Interaction NS
	Co(m)-HNE	93.5 ± 7.5 ^**b**^		91.7 ± 10.4	
	Co(c)-NT	152.1 ± 19.5 ^**a**^		108.9 ± 9.6	
	Co(c)-HNE	158.7 ± 12.3 ^**a**^		126.5 ± 9.2	
**p21**	Co(m)-NT	100.0 ± 2.0 ^**b**^	HNE treatment NS **Monoculture vs coculture p = 0.0035** Interaction NS	100.0 ± 8.6 ^b,c^	HNE treatment NS **Monoculture vs coculture p = 0.0046** Interaction NS
	Co(m)-HNE	102.3 ± 6.9 ^**a,b**^		89.9 ± 5.2 ^c^	
	Co(c)-NT	153.5 ± 19.3 ^**a,b**^		119.0 ± 8.6 ^a,b^	
	Co(c)-HNE	161.0 ± 17.1 ^**a**^		130.7 ± 8.5 ^**a**^	

Co colonocytes were treated in monoculture (m) or in coculture (c). Western blot analysis was performed at D7 and D21 of the protocol. The results were normalized and expressed as the mean ± SEM of at least 3 experiments. Two-way ANOVA was performed followed by Tukey’s multiple comparisons test. The same letters indicate no significant difference between the groups.

The Wnt signaling pathway is involved in the development of many types of cancer, including CRC. As shown in Fig 3H–3K, the coculture protocol modulated the mRNA levels of Apc, Ctnnb1, Wnt2b and Ccnd1 in Co cells. The mRNA level of the target Ccnd1 was significantly lower in Co(c)-HNE cells than in Co(m)-NT and Co(m)-HNE cells (Fig 3K). In fibroblasts, Wnt2b expression was modified by 4-HNE treatment, while Wnt5a expression was affected by the cell culture protocol (S7 Fig). The expression of Notch2 and its downstream target Hes1 was modulated in Co cells by the cell culture protocol (S8 Fig). Notch 2 and Hes1 expression was greater in Co(c)-NT cells than in Co(m)-NT colonocytes. Finally, a decrease in the mRNA level of prominin1 was observed in nF fibroblasts when these cells were cocultured with Co colonocytes (S9 Fig).

### 3.3 Impact of repeated 4-HNE exposure of murine colonocytes in monoculture or coculture on their ability to undergo anchorage-independent growth (Fig 1A)

Preliminary soft agar assays were performed under both restrictive (37°C, no IFγ) and permissive (33°C, with IFγ) culture conditions. As shown in S10 Fig, Co(m) cells previously exposed to 4-HNE had a greater ability to grow in an anchorage-independent manner, with a doubling of the total number of colonies observed compared to untreated Co(m) cells, regardless of whether the soft agar assays were performed under permissive or restrictive conditions. Subsequent trials were carried out at 33°C under permissive conditions to enable fibroblasts to be maintained in culture inserts.

**Fig 5:**
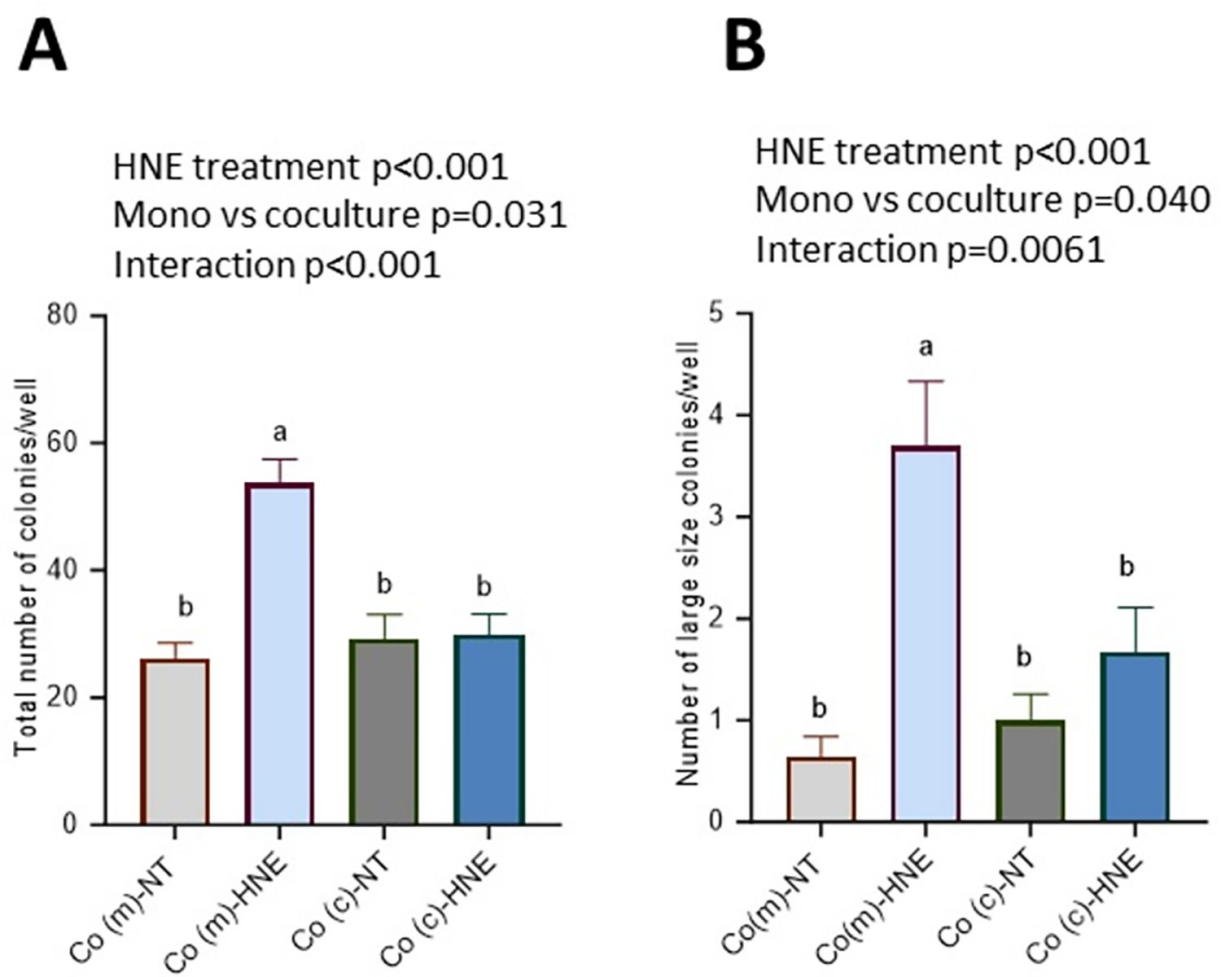
Epithelial–mesenchymal dialogue protects colonocytes from repeated exposure to 4-HNE. Co cells were grown in monoculture (m) or in coculture (c) with nF fibroblasts and exposed twice weekly to a noncytotoxic concentration of 4-HNE (5 μM, 1 h) in SVF-free medium or to an equivalent volume of SVF-free medium as a control (NT). After 3 weeks, the cells were resuspended in soft agar at a density of 10^3^ cells/ml and grown for 21 days. Cells were not exposed to 4-HNE during the soft agar assay. **A/** Total number of Co colonies observed per well and **B/** Number of large size colonies (diameter>100 μm) observed per well. The data are expressed as the means ± SEMs (n = 4 assays in triplicate). Two-way ANOVA was performed followed by Tukey’s multiple comparisons test. The same letters indicate no significant difference between the groups.

As shown in Fig 5, both repeated exposure to 4-HNE and the cell culture protocol (monoculture or coculture) affected the total number of colonies observed; the interaction between these two factors was also significant (p<0.001). Tukey’s post hoc test revealed that the total number of colonies and the number of large colonies were significantly greater for Co(m)-HNE cells than for Co(m)-NT cells, Co(c)-NT cells and Co(c)-HNE cells. No significant difference was observed between Co(c)-HNE and Co(c)-NT cells.

### 3.4 Culturing nF(m) fibroblasts on inserts during the soft agar assay increased the anchorage-independent growth ability of Co(m) cells previously exposed or not to 4-HNE

**Fig 6:**
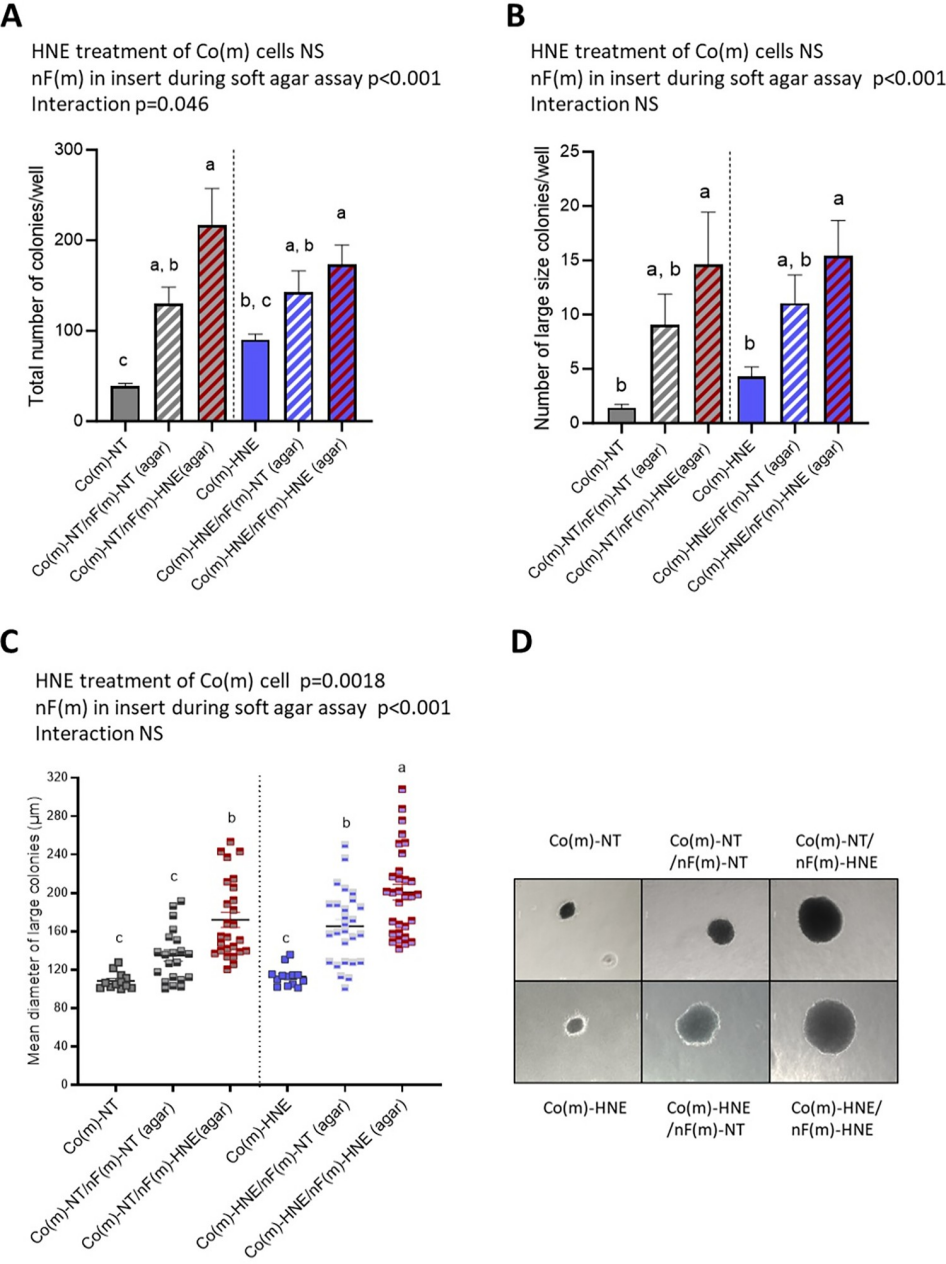
Effects induced by the presence of nF(m) fibroblasts during the soft agar assay. Co and nF cells were grown in monoculture (m) and exposed twice weekly to a noncytotoxic dose of 4-HNE (5 μM) in SVF-free medium or to an equivalent volume of SVF-free medium as a control (NT). At the end of the 3 weeks, the Co(m) cells were suspended in soft agar at a density of 10^3^ cells/ml. nF(m) fibroblasts that had also been exposed or not exposed to 4-HNE in monoculture were seeded in Thincert™ tissue culture inserts and placed on top of the agar wells. 4-HNE was not added during the soft agar assay. The data are expressed as the means ± SEMs (n = 4 assays in triplicate). Two-way ANOVA was performed followed by Tukey’s multiple comparisons test. The same letters indicate no significant difference between the groups. **A/** Total number of colonies per well. **B**/ Number of large size colonies (diameter>100 μm) per well. **C**/ Mean diameter of large colonies: At least 15 randomly selected structures in three independent experiments were measured. **D**/ Representative images of large colonies observed in soft agar.

In our protocol, fibroblasts were not seeded in soft agar but were deposited in culture inserts above the wells containing a mixture of soft agar and Co cells (Fig 1B). The Co(m) and nF(m) cells were not in contact and communicated only via the culture medium. The presence of nF(m)-HNE fibroblasts during the soft agar assay increased the ability of Co(m)-NT and Co(m)-HNE cells to grow without anchorage, with fivefold and twofold increases, respectively, in the total number of colonies observed compared to Co(m)-NT and Co(m)-HNE cells grown alone in soft agar (Fig 6A). The presence of nF(m)-HNE fibroblasts also significantly increased the number and mean diameter of large colonies (Fig 6B–6D and S11 Fig).

Surprisingly, nF(m)-NT fibroblasts also promoted anchorage-independent growth of Co(m)-NT (Fig 6A). However, they had no significant effect on the number of large Co(m)-NT colonies (Fig 6B). The presence of nF(m)-NT fibroblasts altered neither the total number of colonies nor the number of large colonies of Co(m)-HNE cells observed, but their presence led to an increase in the mean diameter of these colonies (Fig 6C and 6D).

### 3.5 nF(c)-HNE fibroblasts cultured on inserts during the soft agar assay increased anchorage-independent growth ability of Co(c)-HNE (Fig 1C)

**Fig 7:**
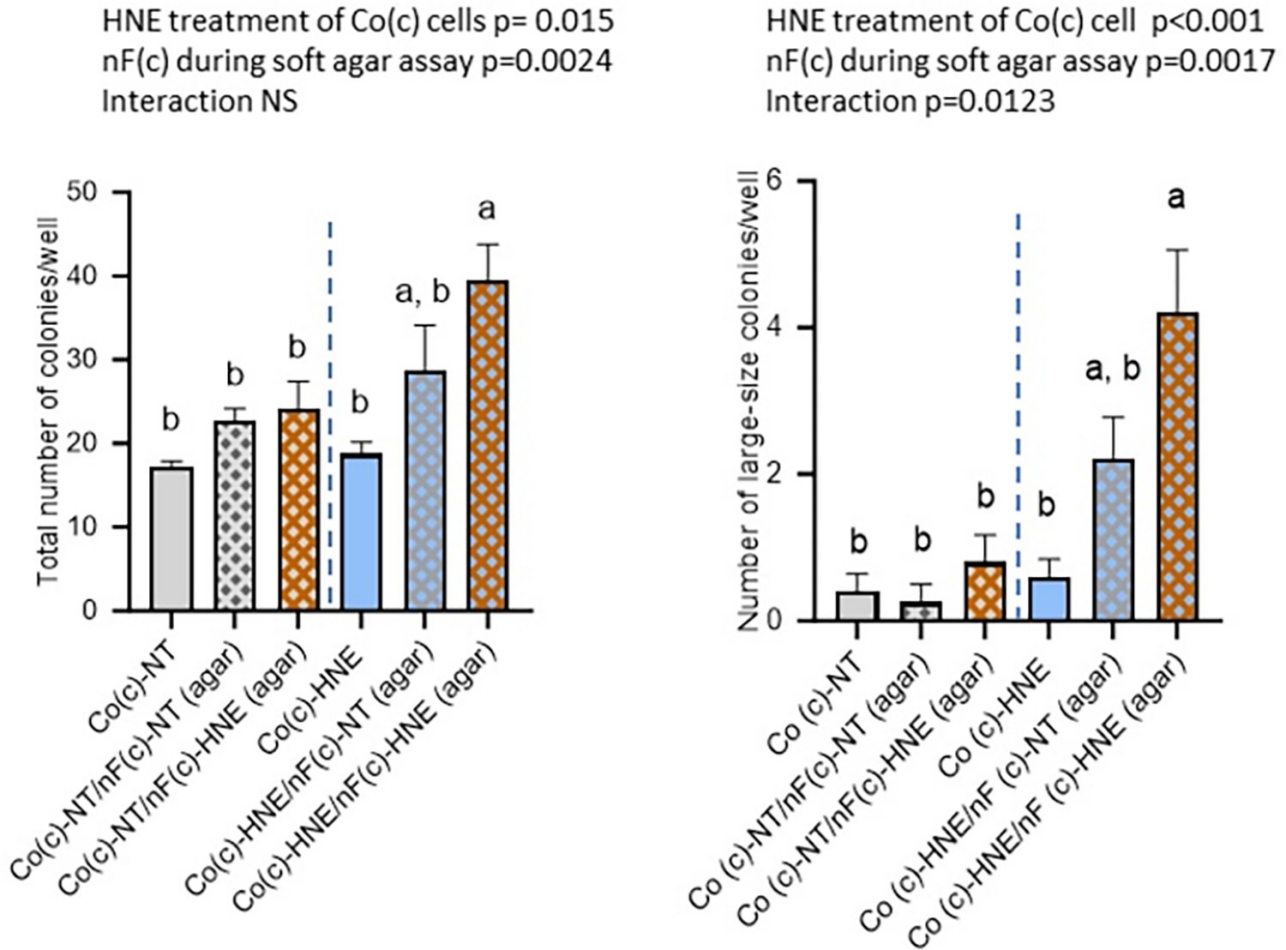
Effects induced by the presence of nF(c) fibroblasts during the soft agar assay. Co cells were cocultured with nF fibroblasts and exposed twice weekly to a noncytotoxic concentration of 4-HNE (5 μM) in SVF-free medium or to an equivalent volume of SVF-free medium as a control (NT). At the end of the 3 weeks, the Co(c) cells were resuspended in soft agar gels at a density of 10^3^ cells/ml. The cocultured fibroblasts nF(c) (exposed or not to 4-HNE) were seeded in Thincert™ culture inserts and placed (or not) on top of the wells. 4-HNE was not added during the soft agar assay. The data are presented as the means ± SEMs (n = 4 assays in triplicate). Two-way ANOVA was performed followed by Tukey’s multiple comparisons test. The same letters indicate no significant difference between the groups. **A**/ Total number of colonies per well. **B/** Number of large colonies per well (diameter>100 μm).

The results of the two-way ANOVA showed that the 4-HNE treatment phase of Co(c) cells but also the presence of nF(c) fibroblasts during the soft agar assay modulated the number of Co colonies observed (Fig 7). The presence of nF(c)-HNE fibroblasts during the soft agar assay increased the total number of Co(c)-HNE colonies, as well as the number of large and medium-sized colonies, compared with those of Co(c)-HNE cells without nF(c) fibroblasts in culture inserts (Fig 7). nF(c)-NT fibroblasts did not significantly alter the number or size of Co(c)-NT cell colonies (Fig 7). By interchanging fibroblasts during the soft agar assay, we showed that nF(c)-HNE fibroblasts did not increase the ability of Co(c)-NT cells to form colonies in soft agar. Similarly, nF(c)-NT fibroblasts only showed a tendency to increase the anchorage-independent growth ability of Co(c)-HNE cells.

### 3.6 The BMP pathway prevents the phenotypic transformation of normal colonocytes exposed to 4-HNE

**Fig 8:**
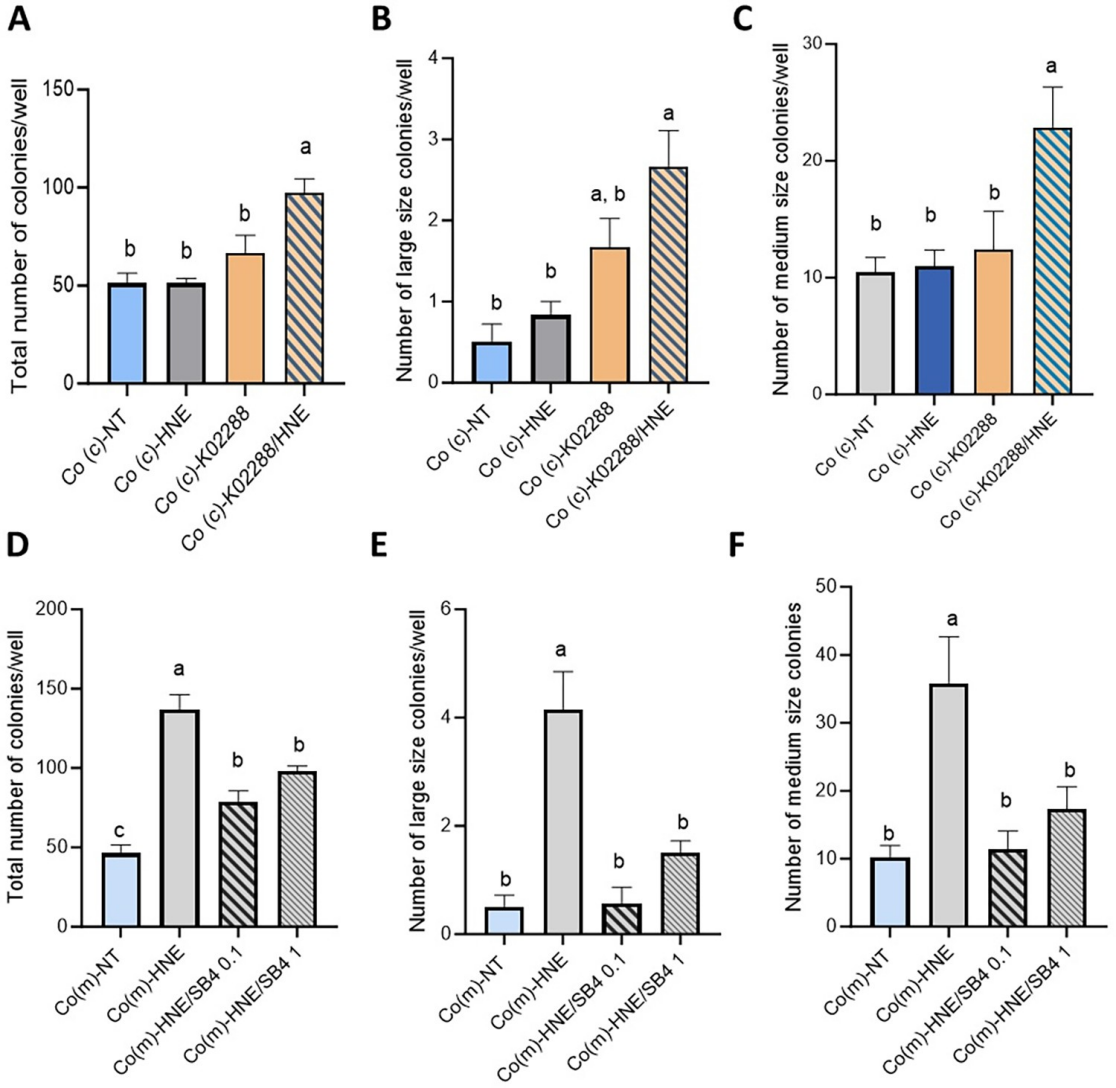
The BMP pathway decreases the ability of colonocytes to grow without anchoring. **A-C**/ Co(c) cells cocultured with nF(c) fibroblasts were treated with the BMP type I receptor inhibitor (K02288) and exposed twice weekly to a noncytotoxic concentration of 4-HNE (5 μM) in SVF-free medium. At the end of 3 weeks, the Co(c) cells were resuspended in soft agar at a density of 10^3^ cells/ml. Neither 4-HNE nor K02288 was added during the soft agar assay. **D-F**/ Co(m) cells cultured in the presence (or absence) of a Bmp4 agonist (SB4) were exposed twice weekly to a noncytotoxic dose of 4-HNE in SVF-free medium. After 3 weeks, the Co(m) cells were resuspended in soft agar at a density of 10^3^ cells/ml. Neither 4-HNE nor SB4 was added during the soft agar assay. The data are presented as the means ± SEMs (n = 4 assays in triplicate). One-way ANOVA was performed followed by Tukey’s multiple comparisons test. The same letters indicate no significant difference between the groups.

Our RT–qPCR results showed that several genes involved in BMP signaling were modulated by coculture or 4-HNE treatment and we wanted to determine the involvement of this regulatory pathway. For this purpose, we continuously exposed Co(c)-HNE cells to the BMP type I receptor inhibitor K02288 during the three weeks of repeated 4-HNE treatment. As shown in Fig 8A–8C, K02288 induced an increase in the total number of Co(c)-HNE colonies observed and in the number of large and medium-sized colonies compared with those of Co(c)-HNE cells that received only the vehicle of the receptor inhibitor for 3 weeks. These effects were not observed when Co(c) cells were treated only with K02288. Furthermore, continuous exposure of Co(m)-HNE cells to SB4, a potent Bmp4 agonist, significantly decreased the total number of colonies observed, as well as the number of large and medium-sized colonies, compared with those of Co(m)-HNE cells that received only the agonist vehicle during the 3 weeks of treatment (Fig 8D–8F).

### 3.7 Exogenous PGE_2_ promoted the phenotypic transformation of normal Co colonocytes

**Fig 9:**
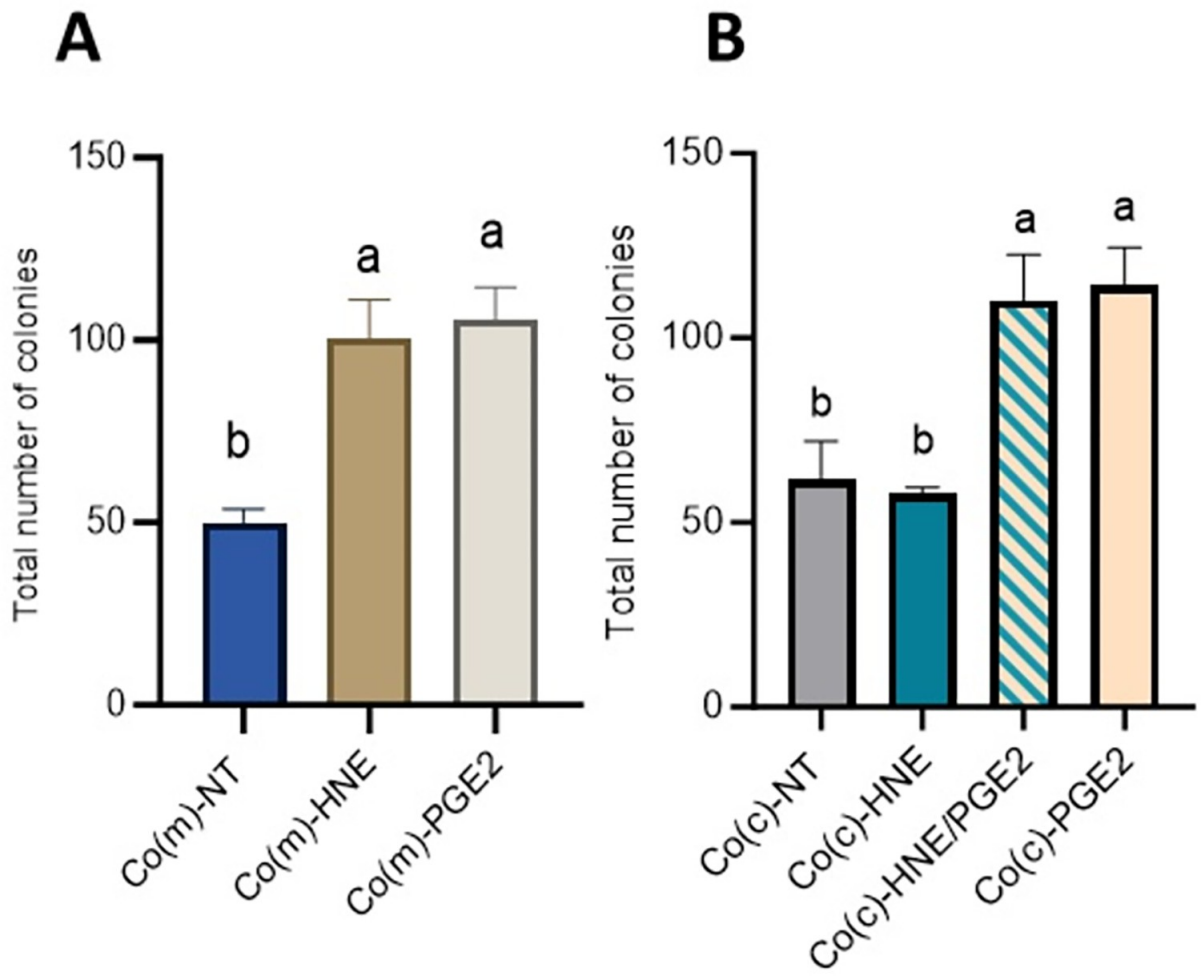
PGE_2_ increased the ability of normal Co cells to form colonies in soft agar. Co cells were grown (**A**) in monoculture or (**B**) in coculture with nF fibroblasts that were treated or not treated with PGE_2_ (1 μM) and exposed twice weekly to a noncytotoxic concentration of 4-HNE (5 μM, 1 h) in SVF-free medium. After 3 weeks, the cells were resuspended in soft agar at a density of 10^3^ cells/ml and grown for 21 days. The data are presented as the means ± SEMs (n = 4 assays in triplicate). One-way ANOVA was performed followed by Tukey’s multiple comparisons test. The same letters indicate no significant difference between the groups.

Repeated exposure of Co(m) cells to PGE_2_ (1 μM) improved their ability to grow without anchoring. The total number of colonies observed was similar to that obtained with Co(m) cells previously treated with 4-HNE (Fig 9A). When Co cells were cocultured with nF fibroblasts, the presence of PGE_2_ in the culture medium led to the formation of colonies in the soft agar assay compared with that in the case of Co(c)-NT or Co(c)-HNE. No difference was observed between the Co(c)-HNE/PGE_2_ and Co(c)-PGE_2_ cells (Fig 9B).

## 4 Discussion

Our study confirmed that highly reactive lipid peroxidation-inducing alkenals have tumorigenic transformation potential on normal colonocytes, since repeated exposure of normal Co cells to a noncytotoxic concentration of 4-HNE for three weeks significantly increased their ability to grow in an anchorage-independent manner in soft agar. The key finding of our study was that when the treatment phase was carried out in the presence of colonic fibroblasts, this deleterious effect of 4-HNE was no longer observed, suggesting that paracrine exchanges between Co and nF cells protected colonocytes. The high interaction between Co and nF during coculture was also reinforced by gene expression analysis. In colonocytes, dialogue with fibroblasts had an impact on two-thirds of the genes studied, whereas less than five percent of these genes were affected by 4-HNE. In particular, cellular crosstalk led to increased expression of not only three BMP receptors (Bmpr1, Bmpr2 and Acvr2a) but also the intracellular effector Smad1, indicating the potential involvement of BMP signaling in this dialogue. The BMP pathway is known to play a crucial role in the control of epithelial stem cell proliferation and differentiation [28]. To this end, BMP ligands trigger an intracellular signaling cascade after binding to membrane receptors of type 1 (BMPR1A, BMPR1B) and type 2 (BMPR-II, Acvr2, etc.) on epithelial cells [29]. Activated BMP receptor complexes then transduce signals via Smad1/5/8, which in turn forms a complex with Smad4, ultimately downregulating target genes such as Ccnd1 [29, 30]. In the intestine, mesenchymal cells, and in particular subepithelial myofibroblasts, are the main BMP ligand-producing cells [16, 17]. BMP ligands can also be produced by other cell populations, particularly intestinal epithelial cells. In our study, Bmp2 and Bmp4 were much less expressed in Co colonocytes than in nF fibroblasts. Moreover, Bmp2 and Bmp4 mRNA levels in Co cells were negatively affected by coculture with fibroblasts. Taken together, these results suggest that when the 4-HNE treatment phase was performed on cocultured cells, Bmp ligands were primarily produced by fibroblasts. In a previous study, we showed that Bmp4 ligands released by nF fibroblasts cocultured with Co cells for 48 h acted on the latter to reduce their proliferation [22]. In line with this, we observed a reduction in Co(c) cell growth during the first two weeks of coculture. More importantly, exposing Co(m) cells to SB4, a potent agonist of Bmp4 signaling, throughout the treatment protocol prevented 4-HNE-induced phenotypic transformation of colonocytes, while exposing Co(c)-HNE cells to a potent BMP type I receptor inhibitor promoted colony formation. All these data establish for the first time that the presence of BMP ligands in the environment of normal colonocytes, either by the presence of fibroblasts or by an exogenous supply of BMP ligands, may inhibit their phenotypic transformation. They also expand the findings of Vishunubalaji et al., who showed that BMP2, but this time throughout the soft agar assay, inhibited the colony-forming ability of HCT116 human colon cancer cells [31]. These results are also consistent with the data showing that a deficiency in the BMP signaling pathway may be strongly involved in sporadic CRC development. Thus, loss of the BMP5 ligand was observed at early stages in the development of sporadic CRC [32], whereas Smad4 mutation was detected at a more advanced stage [33]. Interestingly, inhibition of the BMP pathway combined with simultaneous production of PGE_2_ led to the development of gastric tumors in mice, demonstrating the importance of both pathways in gastrointestinal tumorigenesis [34].

Notably, Co(c) colonocytes in coculture were also characterized by a significant increase in the expression of *Rad51*, *Cdkn1a* (*p21)* and *Pten*, three genes directly or indirectly involved in the repair of DNA damage, particularly in the repair of double-strand breaks [35–37]. As the DNA repair capacity of a cell is vital for maintaining genome stability, this effect of epithelial–mesenchymal dialogue could protect Co(c) cells from the potential genotoxic effects of 4-HNE and could explain the absence of phenotypic transformation. Interestingly, the BMP signaling pathway may be involved in increasing the expression of these genes. Indeed, Vattulainen-Collanus et al highlighted the role of BMP signaling in RAD51 expression [38]. Their work notably showed that loss-of-function mutations in the BMPR2 receptor led to RAD51 depletion, followed by genome instability and mutation. Other studies have revealed that the BMP pathway increases PTEN expression in CRC stem cells [39] and in pulmonary artery smooth muscle cells [40].

Gene expression in fibroblasts was also affected by coculture but to a lesser extent than that in colonocytes (twenty-eight percent of genes studied). In particular, coculture induced in fibroblasts an increase in the expression of Il-6, a microenvironmental factor known to promote cell proliferation [41]. This upregulation may play a role in the faster Co(c) cell growth observed at the end of the repeated treatment protocol (during week 3). Furthermore, numerous genes, including genes related to increased expression of *Nrf2* and decreased expression of the transcriptional repressor *Bach1*, were also affected by 4-HNE treatment in these cell lines. These results suggest that the Nrf2 pathway, which is involved in the adaptive response to 4-HNE, was still activated in fibroblasts at D21.

While epithelial–mesenchymal interaction preserved normal colonocytes from the phenotypic transformation induced by 4-HNE exposure, it clearly appears that the presence of fibroblasts during the soft agar assay provided a favorable environment for the unanchored growth of Co cells and could therefore contribute to the development and progression of CRC. This deleterious effect of fibroblasts was much more pronounced for nF(m) fibroblasts than for nF(c) fibroblasts from coculture, with an increase of both the total number and size of Co(m)-NT and Co(m)-HNE cell colonies. Moreover, this phenomenon was amplified with nF(m) fibroblasts previously exposed to 4-HNE. In the case of cells derived from coculture treatment, only the Co(c)-HNE/nF(c)-HNE combination in the soft agar assay resulted in a small but significant increase in colony number, suggesting that the epithelial–mesenchymal dialogue during the initial treatment phase led to the emergence of a fibroblastic phenotype less favorable for tumor progression. It should also be noted that nF(c)-HNE fibroblasts were characterized by increased Cox2 expression at the end of the treatment protocol. This could explain why their presence during the soft agar assay favored the unanchored growth ability of Co(c)-HNE cells. This hypothesis is consistent with the findings of an elegant study by Roulis et al., who reported that Cox2-expressing pericryptal fibroblasts play a role in CRC initiation by activating the protumorigenic EP4-YAP1 prostaglandin receptor pathway in epithelial stem cells [42].

Interestingly, Cox2 protein expression was also enhanced in monoculture-treated Co(m)-HNE colonocytes. 4-HNE is known to covalently modify numerous target cellular proteins, which may affect their biological function and activate numerous signaling pathways [8–12]. 4-HNE is notably a powerful inducer of the inflammatory mediator Cox2 in various cell types, including rat YPEN endothelial cells, rat RL34 liver cells, mouse 3T3 adipocytes, mouse RAW264.7 macrophages and human chondrocytes [43–47]. The increased Cox2 level observed in Co(m)-HNE cells agrees with the findings of these studies and could explain, at least in part, their phenotypic transformation. In line with this assumption, we showed that chronic exposure of normal Co(m) cells to PGE_2_ augmented their anchorage-independent growth ability. Conversely, the Cox2 protein was not upregulated in Co(c) colonocytes exposed to 4-HNE in coculture, and the ability of these cells to form colonies was not increased as long as the soft agar assay was performed without nF(c)-HNE fibroblasts. Overall, our results suggest a major role for Cox2 in the 4-HNE-induced transformation of normal colonocytes, which could add to the potential mutagenic effects of 4-HNE through the formation of 4-HNE-dG adducts [48].

In addition, the Cox2/PGE_2_ pathway may also be involved in the ability of Co(m)-HNE cells to proliferate and survive in the absence of anchorage. In support of the latter hypothesis, Xu et al. showed that glioma cells overexpressing Cox2 exhibited increased colony growth in soft agar [49]. The importance of this enzyme was also highlighted by another study, which revealed that resveratrol, a polyphenol present in our diet, drastically reduced the capacity of colon cancer HT-29 cells to form colonies in soft agar by targeting Cox2 [50]. Note that extensive in vivo evidence strongly implicates Cox2 in the development and progression of CRC through mechanisms promoting epithelial cell proliferation, resistance to apoptosis and angiogenesis [51, 52]. It is overexpressed in approximately 85% of CRCs and is associated with poor survival [53]. The use of Cox2 inhibitors in animal models of CRC (ApcMin/+ mouse model, AOM-treated mouse) also induces a reduction in the number and size of preneoplastic tumors [54, 55].

The limitations of our study are as follows. First, to maintain the fibroblastic population during the three weeks of the soft agar assay, the cells were cultured under permissive conditions (33°C, with IFγ). Under these culture conditions, nF cells could not possess all the properties of resting fibroblasts. This could explain the large number of colonies observed in the presence of nF(m)-NT fibroblasts. Second, studies have highlighted the possibility of information exchange by direct contact between fibroblasts and epithelial cells [56]. In our experiments, the fibroblasts were seeded in culture inserts during the 4-HNE exposure phase or during the soft agar assay, which allows only paracrine exchanges and cuts us off from some of the information. Finally, our study focused on the interactions between colonocytes and fibroblasts. However, the microenvironment of healthy crypts, like that of tumors, is much more complex and includes not only fibroblasts but also endothelial cells, mesenchymal stem cells and immune cells, which can interact with each other as well as with epithelial cells. However, our cellular model will need to be more complex to consider these different interactions.

## 5 Conclusion

Our study revealed a major role for fibroblasts and paracrine exchanges with colonocytes in the initial stages of epithelial cell transformation toward a tumor phenotype. First, epithelial–mesenchymal interactions appear to protect normal colonocytes from 4-HNE-induced phenotypic transformation through a mechanism involving the BMP signaling pathway. On the other hand, however, fibroblasts provide a favorable environment for unanchored growth of epithelial cells and could therefore contribute to tumor development and progression (Table 5). This effect was enhanced in fibroblasts previously exposed to 4-HNE, suggesting that microenvironment-induced changes in fibroblasts could contribute to epithelial transformation and tumor development. Finally, our study suggested that fibroblasts are much more responsive to external factors than colonocytes are (28% 4-HNE-modulated genes vs. 5% for colonocytes) and may therefore play a driving role in tumor progression.

**Table 5:** Summary of main results.

Co Colonocytes		Impact of repeated exposure to 4-HNE according to culture method (mono or co-culture)		Soft agar without nF fibroblasts	Soft agar with nF Fibroblasts	
					nF-NT	nF-HNE
		Impact of the culture cell method	Impact of 4-HNE	Total number of colonies observed in soft agar compared with colonies of Co(m)-NT		
**Co(m) cells**	NT	Co cells in coculture with nF fibroblasts had an increased expression of 1/ DNA guardians (Rad51, Pten, Cdkn1a) and 2/ BMP receptors.	4-HNE increased Cox2 protein level in Co(m) cells	Reference value	↑↑	↑↑↑↑
	HNE			↑	↑↑	↑↑↑
				Total number of colonies observed in soft agar compared with colonies of Co(c)-NT		
**Co(c) cells**	NT		HNE increased Cox2 protein level in nF(c) cells	Reference value	ns	ns
	HNE			ns	ns	↑

The arrows indicate significant increases. Ns = no significant

## Supporting information

S1 Fig Effect of 4-HNE on cellular cytotoxicity and genotoxicity. A/ Effect of 4-HNE on cytotoxicity on Co cells. Data are expressed as the mean ± SEM (n = 3). Cell viability assay was done by using cell proliferation reagent WST-1 (Roche Life Science) according to manufacturer’s protocol. Briefly, cells were seeded at the density of 1.3 × 10^4^ cells/ well in 96-well plates and cultured under permissive conditions (33°, IFN-γ) until sub confluence occurred, and then transferred to non-permissive conditions (37°C, without IFN-γ) for 24 h. When cells were confluent, the culture media were replaced with 4-HNE (10–80 μM) in DMEM. Following 24-hour treatments with 4-HNE at 37°C, media were removed and cells were incubated in WST-1 reagent for 1 h in the dark. The absorbance of each well was measured by colorimetric at measurement and reference wavelengths of 440 nm and 690 nm respectively (TECAN, Infinite 200). B-C/Cellular genotoxicity. Genotoxic effects of increasing concentrations of 4-HNE was measured by γH2AX in-cell Western assay according to Khoury et al (S1 Reference). Cells were seeded into 96-well plates at 4×10^3^ cells per well in permissive conditions. After reached sub confluence, cells were transferred to 37°C and treated with 4-HNE (0, 1, 5 or 10 μM) for 24h. The results are expressed as γH2AX fold induction. **(B**) Co cells and (**C**) nF cells. Data are expressed as the mean ± SEM (n = 3).
(TIF)

S2 Fig Immunofluorescence detection of Cox2 in Co and nF cells. Percentage of Cox2 positive Co cells (A) and nF cells (B). At the end of the treatment phase, the different cell populations were trypsinized. Some cells were used for immunofluorescence assays on slides. Alexa Fluor 594 goat anti-rabbit were used for secondary antibody and DAPI staining for nucleus. Data were expressed as the mean ± SEM of 4 experiments (in duplicate). Two-way ANOVA was performed, followed by Tukey’s multiple comparisons test. The same letters indicate no significant difference between the groups. (C) Representative images of Cox2 positive nF cells were shown. Scale bar shows 50 μm.
(TIF)

S3 Fig αSMA in nF cells exposed to 4-HNE in monoculture (m) or in coculture (c). **(A)** Percentage of αSMA positive nF cells at D21. Data were expressed as the mean ± SEM of 4 experiments (in duplicate). **(B)** Representative images of αSMA positive nF cells at time D21. Alexa Fluor 594 goat anti-rabbit were used for secondary antibody and DAPI staining for nucleus. (**C and D**) Relative expression of Acta2 mRNA and percent of nF(m) cells positive for αSMA in nF(m) cells (not subjected to repeated serum deprivation), nF(m)-NT and nF(m)-HNE cells. The qPCR data were normalized to the level of the hypoxanthine-guanine phosphoribosyltransferase (Hprt1) messenger RNA (mRNA) and analysed via LinRegPCR v.11 software. Data are expressed as the mean ± SEM (n = 3 in triplicate). The same letters indicate no significant difference between the groups.
(TIF)

S4 Fig Relative mRNA expression of Nfe2l2, Bach1, Keap1 and Gpx4 in Co and nF cells at the end of 3 weeks of repeated 4-HNE treatment. qPCR data were normalized to the level of Hprt1 mRNA and analysed via LinRegPCR v.11 software. Data are expressed as the mean ± SEM (n = 3 in triplicate). Two-way ANOVA was performed, followed by Tukey’s multiple comparisons test. The same letters indicate no significant difference between the groups.
(TIF)

S5 Fig Expression of genes involved in the BMP signaling pathway in nF cells. Relative mRNA expression of Bmp2, Bmp4, Grem1, Bmpr1, Bmpr2, Acvr2a, Acvr2b and Smad1 in nF cells at the end of 3 weeks of 4-HNE treatment. qPCR data were normalized to the level of Hprt1 mRNA and analyzed via LinRegPCR v.11 software. Data are expressed as the mean ± SEM (n = 3 in triplicate). Two-way ANOVA was performed, followed by Tukey’s multiple comparisons test. The same letters indicate no significant difference between the groups.
(TIF)

S6 Fig Detection of Bmp4 and Bmp2. **A and B: Immunofluorescence detection of Bmp4 nF cells:** At the end of the three weeks of treatment, nF cells were trypsinized, seeded on an 8-well slide and allowed to incubate for 24 h before being fixed. Alexa Fluor 594 goat anti-rabbit were used for secondary antibody and DAPI staining for nucleus. (A) Percentage of Bmp4 positive nF cells. Data were expressed as the means ± SEMs. Two-way ANOVA was performed, followed by Tukey’s multiple comparisons test. The same letters indicate no significant difference between the groups. (B) Representative images of Bmp4 positive nF cells were shown. Scale bar shows 50 μm. **C/ Dot blot assay with anti-Bmp2 antibody**. The cell culture supernatant was collected before the trypsin phase and then frozen until use. Twenty μl of each supernatant was placed on a nitrocellulose membrane and allowed to dry under a lamp. After this phase, the membranes were treated with the same protocol as for the Western blot.
(TIF)

S7 Fig Expression of genes involved in the Wnt/β-catenin pathway in nF fibroblasts. Relative mRNA expression of Apc, Ctnnb1, Wnt2b, Wnt5a and Ccnd1 in nF cells at the end of 3 weeks of repeated 4-HNE treatment. qPCR data were normalized to the level of Hprt1 mRNA and analysed via LinRegPCR v.11 software. Data are expressed as the mean ± SEM (n = 3 in triplicate). Two-way ANOVA was performed, followed by Tukey’s multiple comparisons test. The same letters indicate no significant difference between the groups.
(TIF)

S8 Fig Expression of genes involved in the Notch pathway in Co colonocytes and nF fibroblasts. Relative mRNA expression of Notch1, Notch2, Hes1, Klf4, Col1a1 and Col1a2 in Co and/or nF cells at the end of 3 weeks of repeated 4-HNE treatment. qPCR data were normalized to the level of Hprt1 mRNA and analysed via LinRegPCR v.11 software. Data are expressed as the mean ± SEM (n = 3 in triplicate). Two-way ANOVA was performed, followed by Tukey’s multiple comparisons test. The same letters indicate no significant difference between the groups.
(TIF)

S9 Fig Expression of CD44 and Prom1 in Co and nF cells. Relative mRNA expression of CD44 and Prom1 in Co colonocytes (A) and nF fibroblasts (B) at the end of 3 weeks of repeated 4-HNE treatment. qPCR data were normalized to the level of Hprt1 mRNA and analyzed via LinRegPCR v.11 software. Data are expressed as the mean ± SEM (n = 3 in triplicate). Two-way ANOVA was performed followed by Tukey’s multiple comparisons test. The same letters indicate no significant difference between the groups.
(TIF)

S10 Fig Effect of culture conditions (permissive vs restrictive) during the soft agar assay. Co cells were grown in monoculture (m) and exposed twice weekly to a non-cytotoxic dose of 4-HNE (5 μM, 1h) in SVF-free medium or to an equivalent volume of SVF-free medium as a control (NT). At the end of the 3 weeks, cells were resuspended in soft agar at a density of 10^3^ cells/ml and grown for 21 days in permissive condition (33°C, IFNγ) or restrictive condition (37°C, without IFNγ). Cells were not exposed to 4-HNE during the soft agar assay. Data are expressed as the means ± SEM (n = 4 assays in triplicate). A one ANOVA was performed, followed by Tukey’s multiple comparisons test. The same letters indicate no significant difference between the groups.
(TIF)

S11 Fig Example of Co cell colonies in soft agar. Co cells in a top layer of 0.35% agar medium were grown for 3 weeks without (A) or with (B and C) fibroblasts seeded onto Thincert™-Tissue culture inserts and placed on top of the agar wells. The wells were then stained with 0.1% crystal violet solution for 10 minutes at room temperature. The largest Co cell colonies were visible to the naked eye.
(TIF)

S1 Raw images Raw images of western blot. Raw blot data related to Tables 1 and 4 and S3 Table.
(PDF)

S1 Table Primer sequences for the quantitative PCR. (DOCX)

S2 Table List of antibodies for western blot and immunofluorescence. (DOCX)

S3 Table Western blot analysis and Relative mRNA expression of cytokeratin 18, Vimentin and αSMA in Co or nF exposed to 4-HNE in monoculture (m) or in coculture (c) at time D7 and D21 of the protocol. Western blot results were normalized and expressed as the mean ± SEM of at least 3 experiments. A two-way ANOVA was performed followed by Tukey’s multiple comparisons test. The same letters indicate no significant difference between the groups. qPCR data were normalized to the level of Hprt1 mRNA and analyzed via LinRegPCR v.11 software. Data are expressed as the mean ± SEM (n = 3 in triplicate). Two-way ANOVA was performed, followed by Tukey’s multiple comparisons test. The same letters indicate no significant difference between the groups.
(DOCX)

S4 Table Relative mRNA expression of Tnfα, Tnfrsf1a and Tnfrsf1b in Co and nF cells at the end of 3 weeks of repeated 4-HNE treatment. qPCR data were normalized to the level of Hprt1 mRNA and analyzed via LinRegPCR v.11 software. Data are expressed as the mean ± SEM (n = 3 in triplicate). A two-way ANOVA was performed followed by Tukey’s multiple comparisons test. Same letters indicate no significant difference between the groups. ne: not expressed.
(DOCX)

S5 Table Relative mRNA expression of Egfr, Il6, Tgfb1 and intracellular effector Smad2 in Co colonocytes and nF fibroblasts at the end of 3 weeks of 4-HNE treatment. qPCR data were normalized to the level of Hprt1 mRNA and analyzed using LinRegPCR v.11 software. Data are expressed as the mean ± SEM (n = 3 in triplicate). Two-way ANOVA was performed followed by Tukey’s multiple comparisons test. The same letters indicate no significant difference between the groups.
(DOCX)

S1 File (DOCX)
