# Supplementary figures and images for: Epithelial-mesenchymal interaction protects normal colonocytes from 4-HNE-induced phenotypic transformation
Source: PLoS One. 2024 Apr 26;19(4):e0302932. doi: 10.1371/journal.pone.0302932 (PMC11051638; doi:10.1371/journal.pone.0302932)

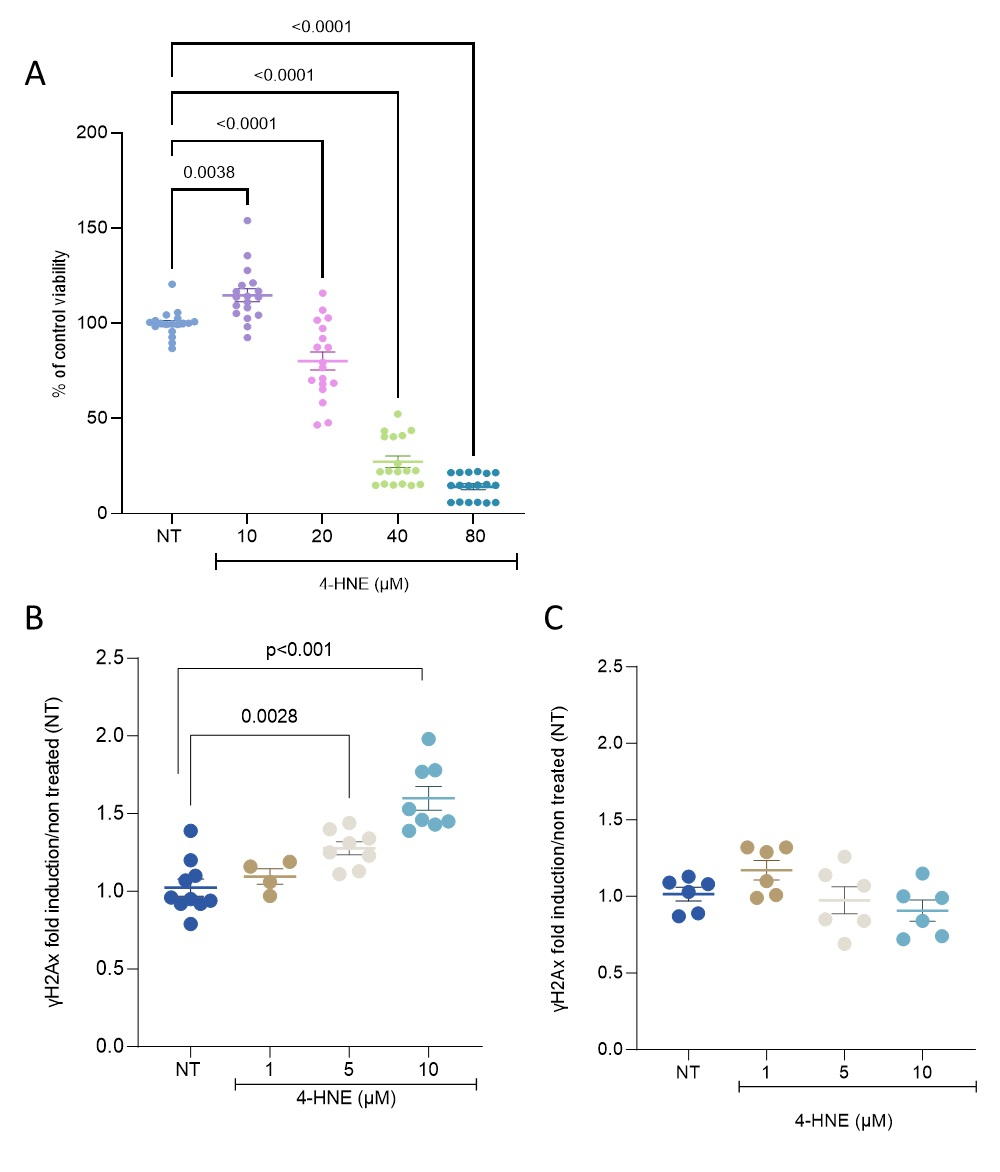

Supplement: S1 Fig — A/ Effect of 4-HNE on cytotoxicity on Co cells. Data are expressed as the mean ± SEM (n = 3). Cell viability assay was done by using cell proliferation reagent WST-1 (Roche Life Science) according to manufacturer’s protocol. Briefly, cells were seeded at the density of 1.3 × 10^4^ cells/ well in 96-well plates and cultured under permissive conditions (33°, IFN-γ) until sub confluence occurred, and then transferred to non-permissive conditions (37°C, without IFN-γ) for 24 h. When cells were confluent, the culture media were replaced with 4-HNE (10–80 μM) in DMEM. Following 24-hour treatments with 4-HNE at 37°C, media were removed and cells were incubated in WST-1 reagent for 1 h in the dark. The absorbance of each well was measured by colorimetric at measurement and reference wavelengths of 440 nm and 690 nm respectively (TECAN, Infinite 200). B-C/Cellular genotoxicity. Genotoxic effects of increasing concentrations of 4-HNE was measured by γH2AX in-cell Western assay according to Khoury et al (S1 Reference). Cells were seeded into 96-well plates at 4×10^3^ cells per well in permissive conditions. After reached sub confluence, cells were transferred to 37°C and treated with 4-HNE (0, 1, 5 or 10 μM) for 24h. The results are expressed as γH2AX fold induction. (B) Co cells and (C) nF cells. Data are expressed as the mean ± SEM (n = 3). (TIF) [file pone.0302932.s001.tif]

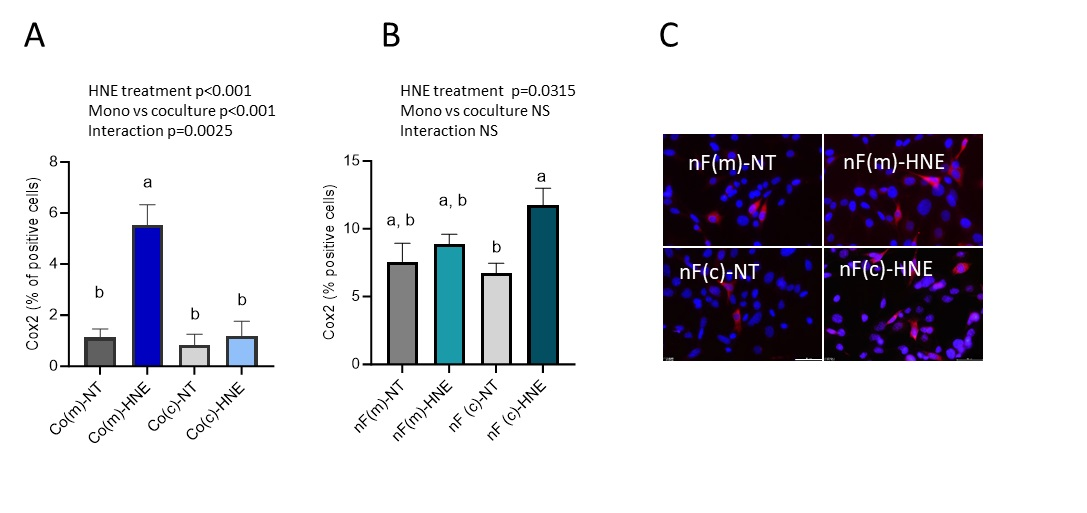

Supplement: S2 Fig — Percentage of Cox2 positive Co cells (A) and nF cells (B). At the end of the treatment phase, the different cell populations were trypsinized. Some cells were used for immunofluorescence assays on slides. Alexa Fluor 594 goat anti-rabbit were used for secondary antibody and DAPI staining for nucleus. Data were expressed as the mean ± SEM of 4 experiments (in duplicate). Two-way ANOVA was performed, followed by Tukey’s multiple comparisons test. The same letters indicate no significant difference between the groups. (C) Representative images of Cox2 positive nF cells were shown. Scale bar shows 50 μm. (TIF) [file pone.0302932.s002.tif]

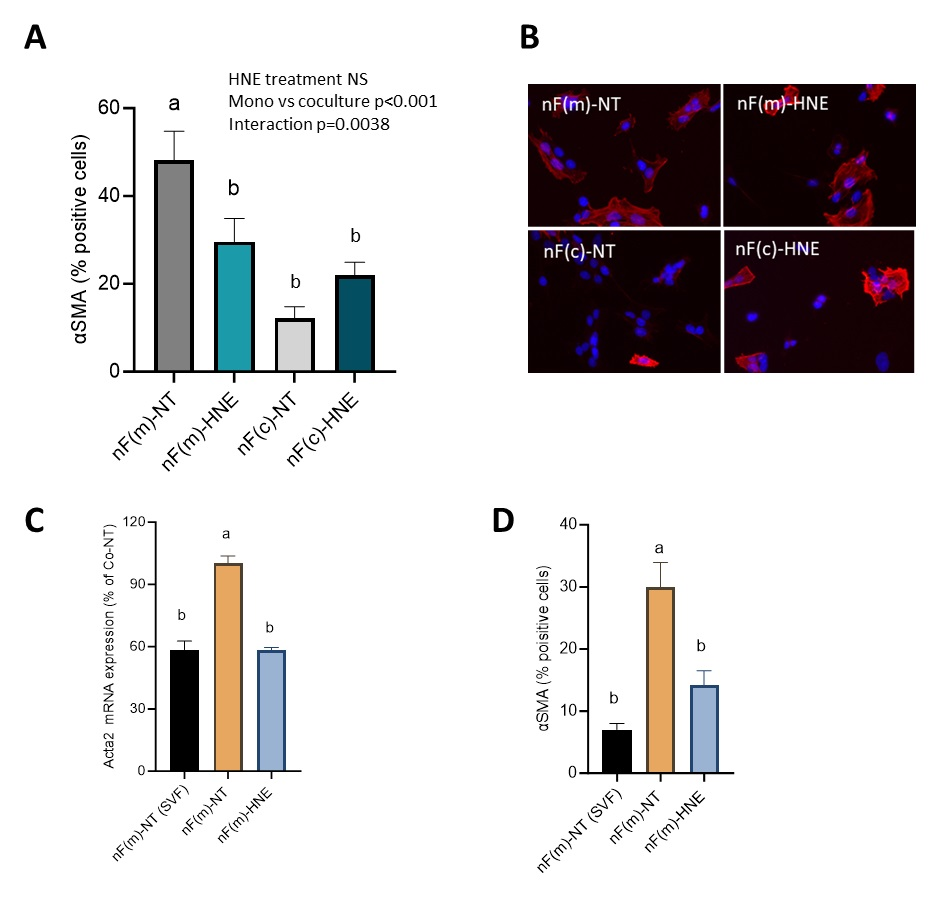

Supplement: S3 Fig — (A) Percentage of αSMA positive nF cells at D21. Data were expressed as the mean ± SEM of 4 experiments (in duplicate). (B) Representative images of αSMA positive nF cells at time D21. Alexa Fluor 594 goat anti-rabbit were used for secondary antibody and DAPI staining for nucleus. (C and D) Relative expression of Acta2 mRNA and percent of nF(m) cells positive for αSMA in nF(m) cells (not subjected to repeated serum deprivation), nF(m)-NT and nF(m)-HNE cells. The qPCR data were normalized to the level of the hypoxanthine-guanine phosphoribosyltransferase (Hprt1) messenger RNA (mRNA) and analysed via LinRegPCR v.11 software. Data are expressed as the mean ± SEM (n = 3 in triplicate). The same letters indicate no significant difference between the groups. (TIF) [file pone.0302932.s003.tif]

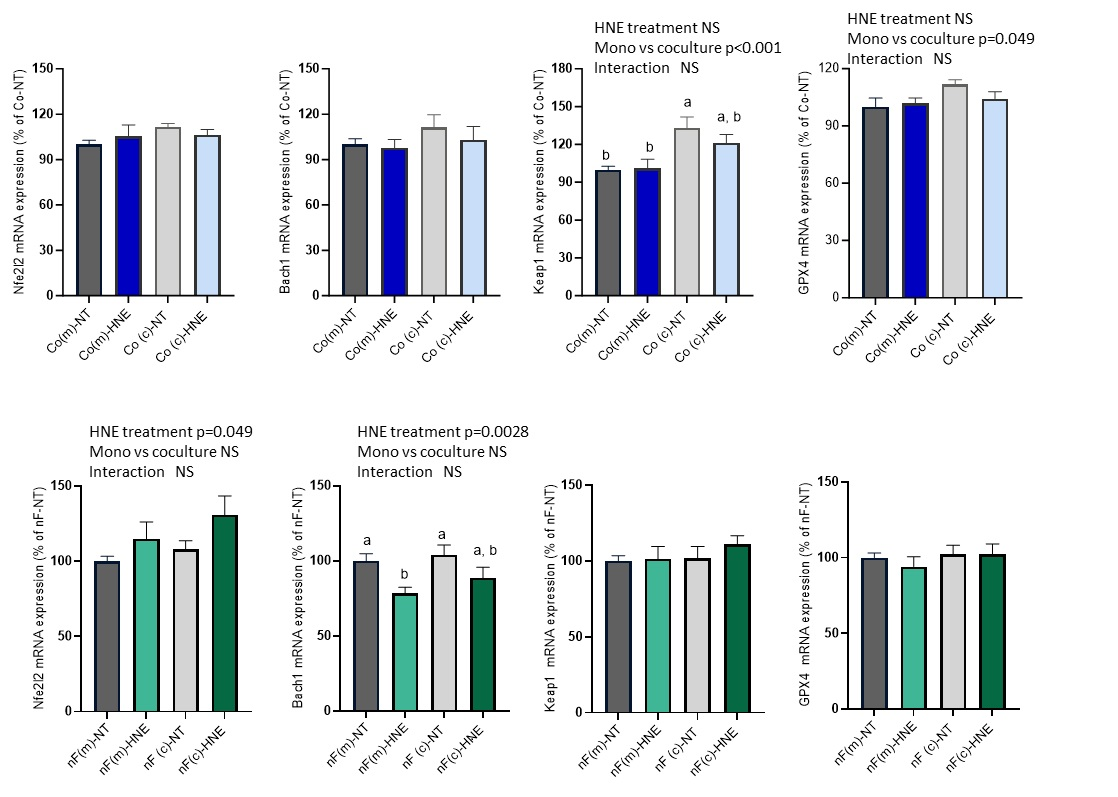

Supplement: S4 Fig — qPCR data were normalized to the level of Hprt1 mRNA and analysed via LinRegPCR v.11 software. Data are expressed as the mean ± SEM (n = 3 in triplicate). Two-way ANOVA was performed, followed by Tukey’s multiple comparisons test. The same letters indicate no significant difference between the groups. (TIF) [file pone.0302932.s004.tif]

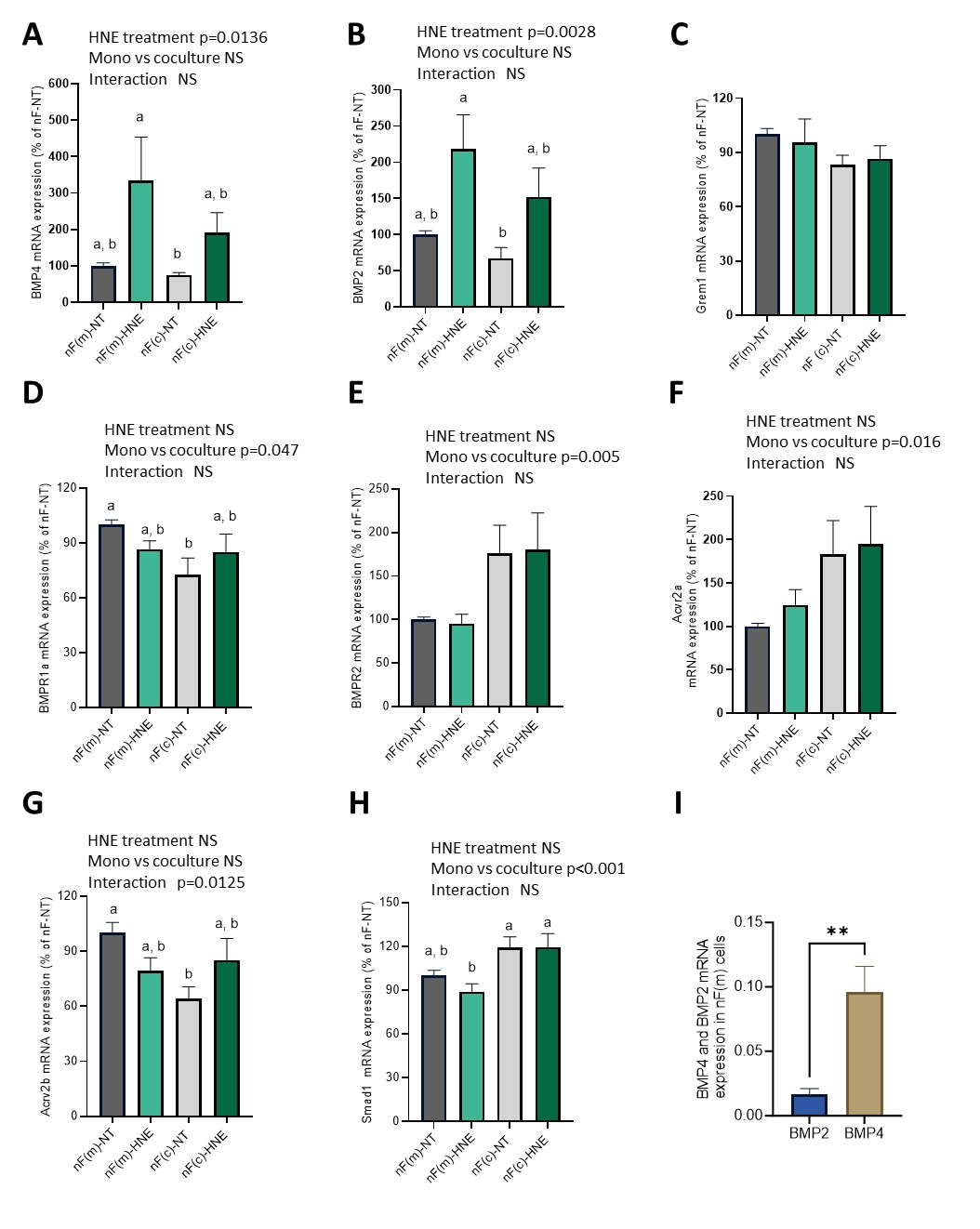

Supplement: S5 Fig — Relative mRNA expression of Bmp2, Bmp4, Grem1, Bmpr1, Bmpr2, Acvr2a, Acvr2b and Smad1 in nF cells at the end of 3 weeks of 4-HNE treatment. qPCR data were normalized to the level of Hprt1 mRNA and analyzed via LinRegPCR v.11 software. Data are expressed as the mean ± SEM (n = 3 in triplicate). Two-way ANOVA was performed, followed by Tukey’s multiple comparisons test. The same letters indicate no significant difference between the groups. (TIF) [file pone.0302932.s005.tif]

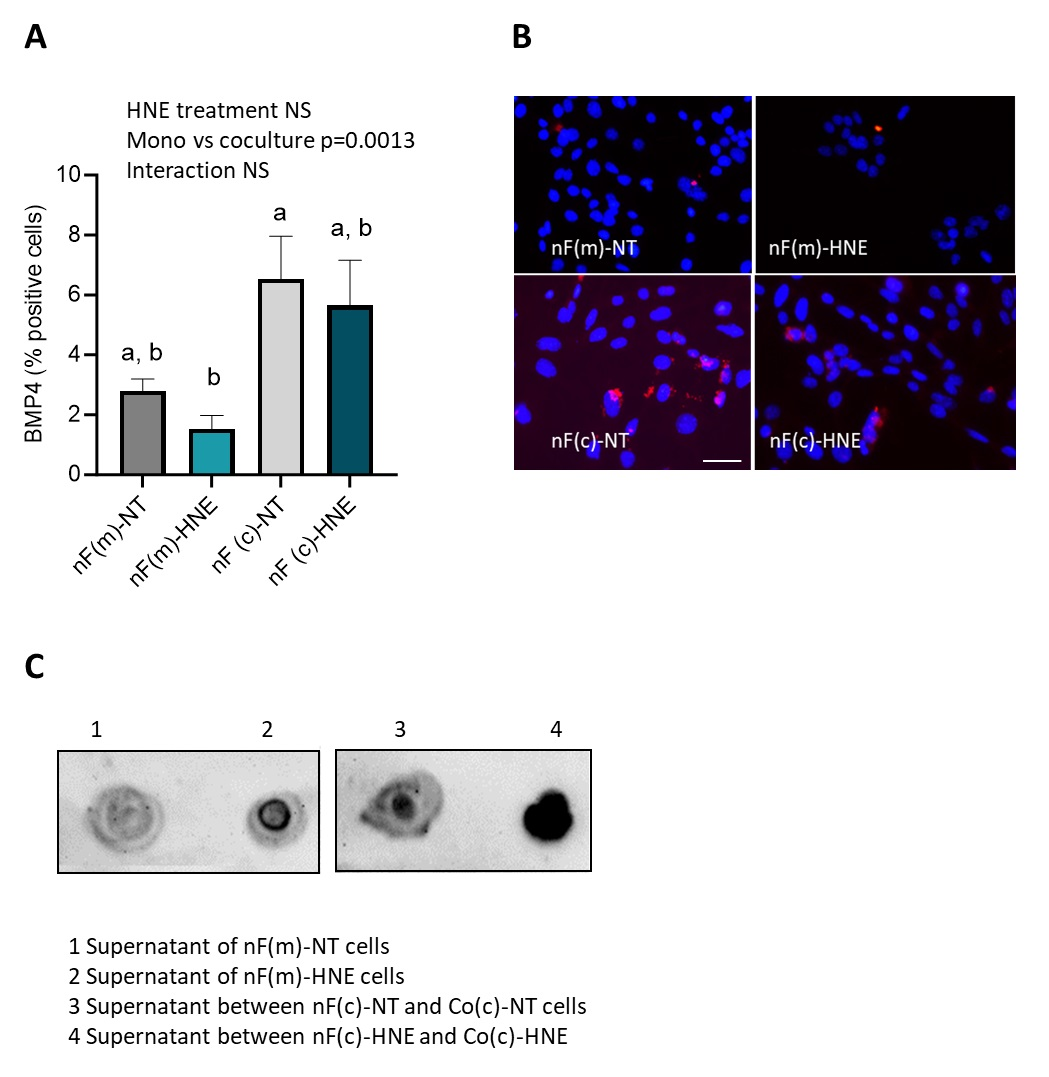

Supplement: S6 Fig — A and B: Immunofluorescence detection of Bmp4 nF cells: At the end of the three weeks of treatment, nF cells were trypsinized, seeded on an 8-well slide and allowed to incubate for 24 h before being fixed. Alexa Fluor 594 goat anti-rabbit were used for secondary antibody and DAPI staining for nucleus. (A) Percentage of Bmp4 positive nF cells. Data were expressed as the means ± SEMs. Two-way ANOVA was performed, followed by Tukey’s multiple comparisons test. The same letters indicate no significant difference between the groups. (B) Representative images of Bmp4 positive nF cells were shown. Scale bar shows 50 μm. C/ Dot blot assay with anti-Bmp2 antibody. The cell culture supernatant was collected before the trypsin phase and then frozen until use. Twenty μl of each supernatant was placed on a nitrocellulose membrane and allowed to dry under a lamp. After this phase, the membranes were treated with the same protocol as for the Western blot. (TIF) [file pone.0302932.s006.tif]

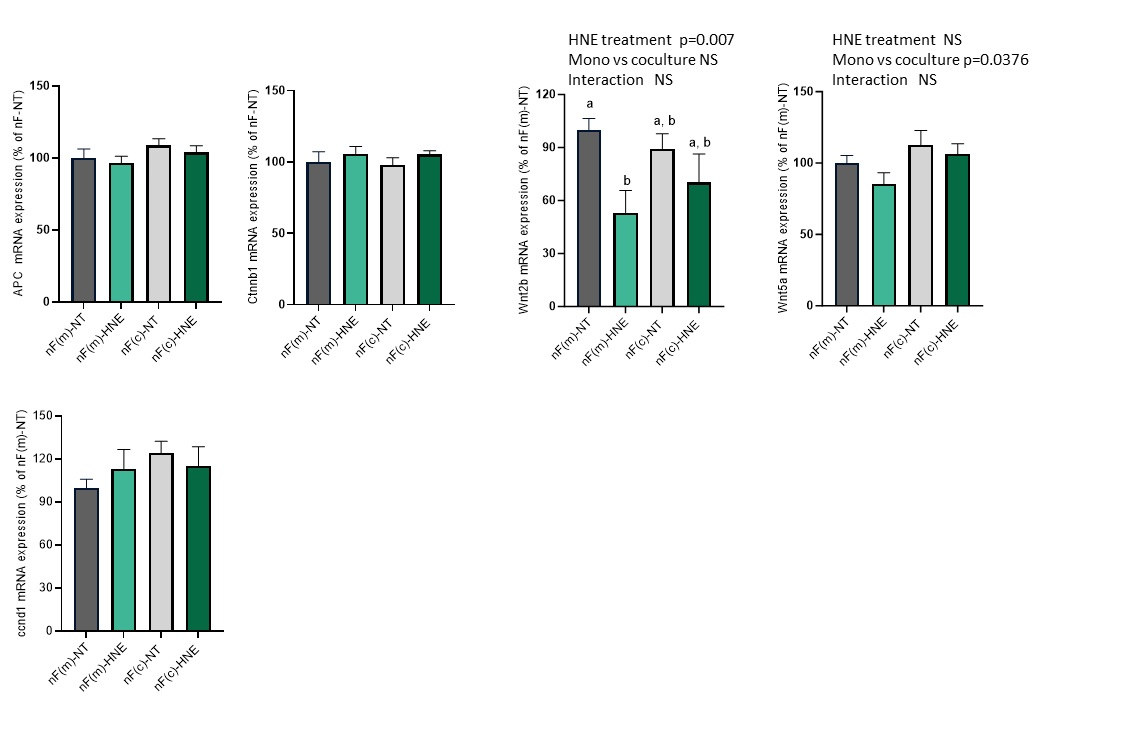

Supplement: S7 Fig — Relative mRNA expression of Apc, Ctnnb1, Wnt2b, Wnt5a and Ccnd1 in nF cells at the end of 3 weeks of repeated 4-HNE treatment. qPCR data were normalized to the level of Hprt1 mRNA and analysed via LinRegPCR v.11 software. Data are expressed as the mean ± SEM (n = 3 in triplicate). Two-way ANOVA was performed, followed by Tukey’s multiple comparisons test. The same letters indicate no significant difference between the groups. (TIF) [file pone.0302932.s007.tif]

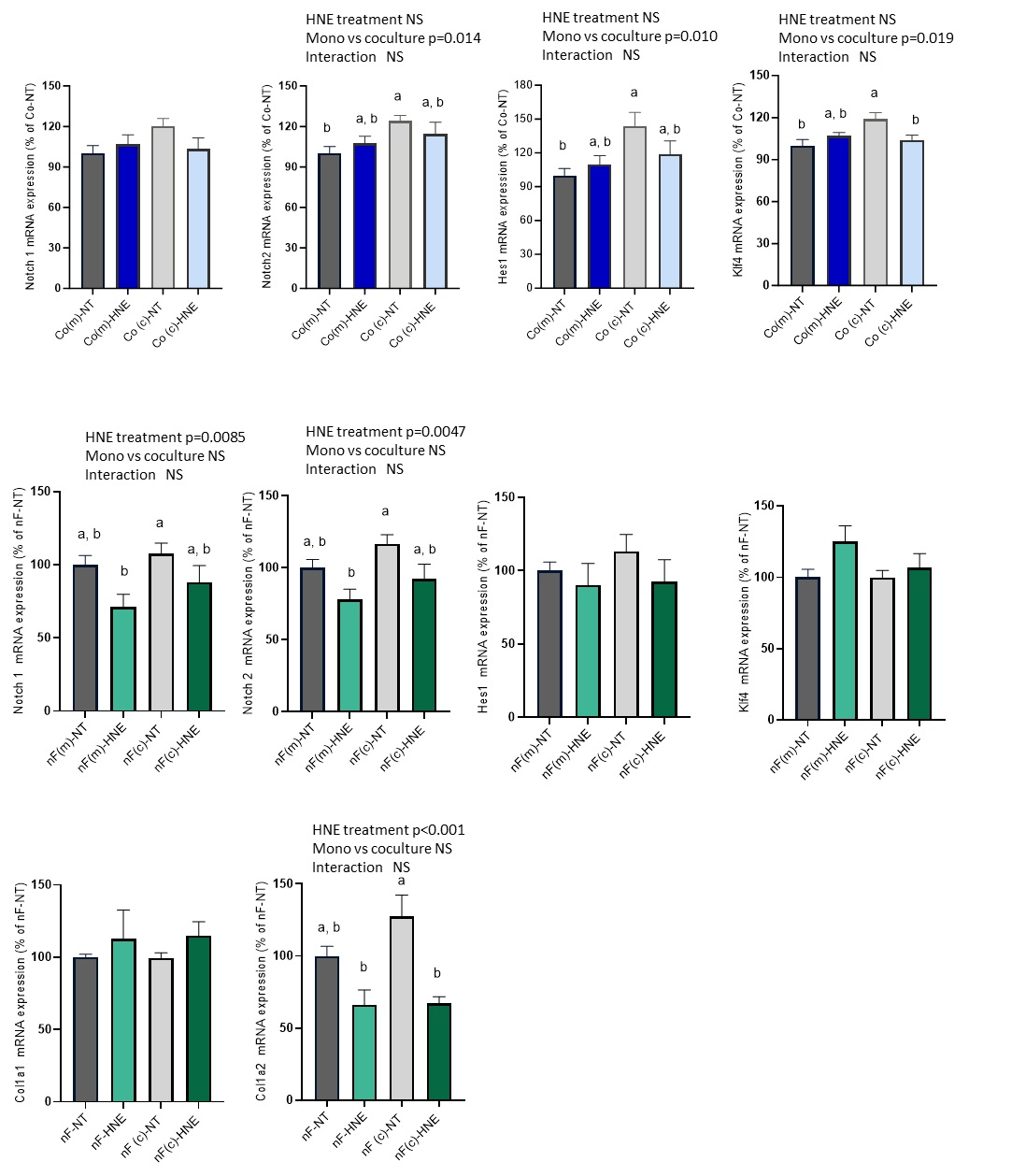

Supplement: S8 Fig — Relative mRNA expression of Notch1, Notch2, Hes1, Klf4, Col1a1 and Col1a2 in Co and/or nF cells at the end of 3 weeks of repeated 4-HNE treatment. qPCR data were normalized to the level of Hprt1 mRNA and analysed via LinRegPCR v.11 software. Data are expressed as the mean ± SEM (n = 3 in triplicate). Two-way ANOVA was performed, followed by Tukey’s multiple comparisons test. The same letters indicate no significant difference between the groups. (TIF) [file pone.0302932.s008.tif]

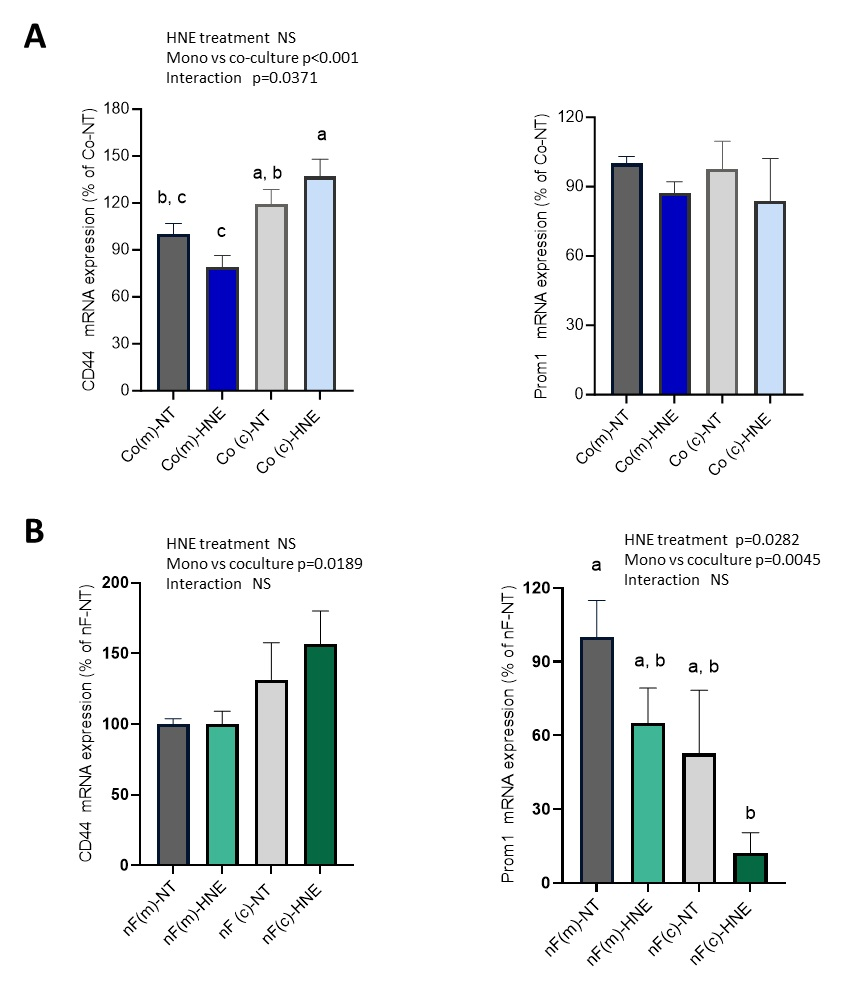

Supplement: S9 Fig — Relative mRNA expression of CD44 and Prom1 in Co colonocytes (A) and nF fibroblasts (B) at the end of 3 weeks of repeated 4-HNE treatment. qPCR data were normalized to the level of Hprt1 mRNA and analyzed via LinRegPCR v.11 software. Data are expressed as the mean ± SEM (n = 3 in triplicate). Two-way ANOVA was performed followed by Tukey’s multiple comparisons test. The same letters indicate no significant difference between the groups. (TIF) [file pone.0302932.s009.tif]

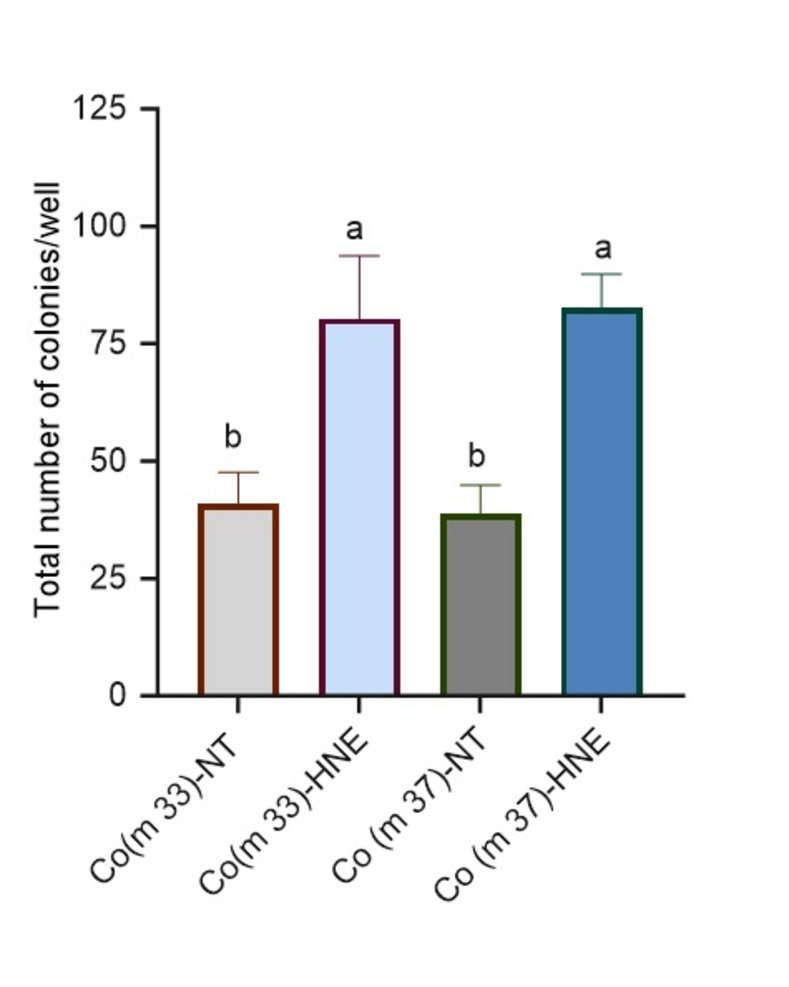

Supplement: S10 Fig — Co cells were grown in monoculture (m) and exposed twice weekly to a non-cytotoxic dose of 4-HNE (5 μM, 1h) in SVF-free medium or to an equivalent volume of SVF-free medium as a control (NT). At the end of the 3 weeks, cells were resuspended in soft agar at a density of 103 cells/ml and grown for 21 days in permissive condition (33°C, IFNγ) or restrictive condition (37°C, without IFNγ). Cells were not exposed to 4-HNE during the soft agar assay. Data are expressed as the means ± SEM (n = 4 assays in triplicate). A one ANOVA was performed, followed by Tukey’s multiple comparisons test. The same letters indicate no significant difference between the groups. (TIF) [file pone.0302932.s010.tif]

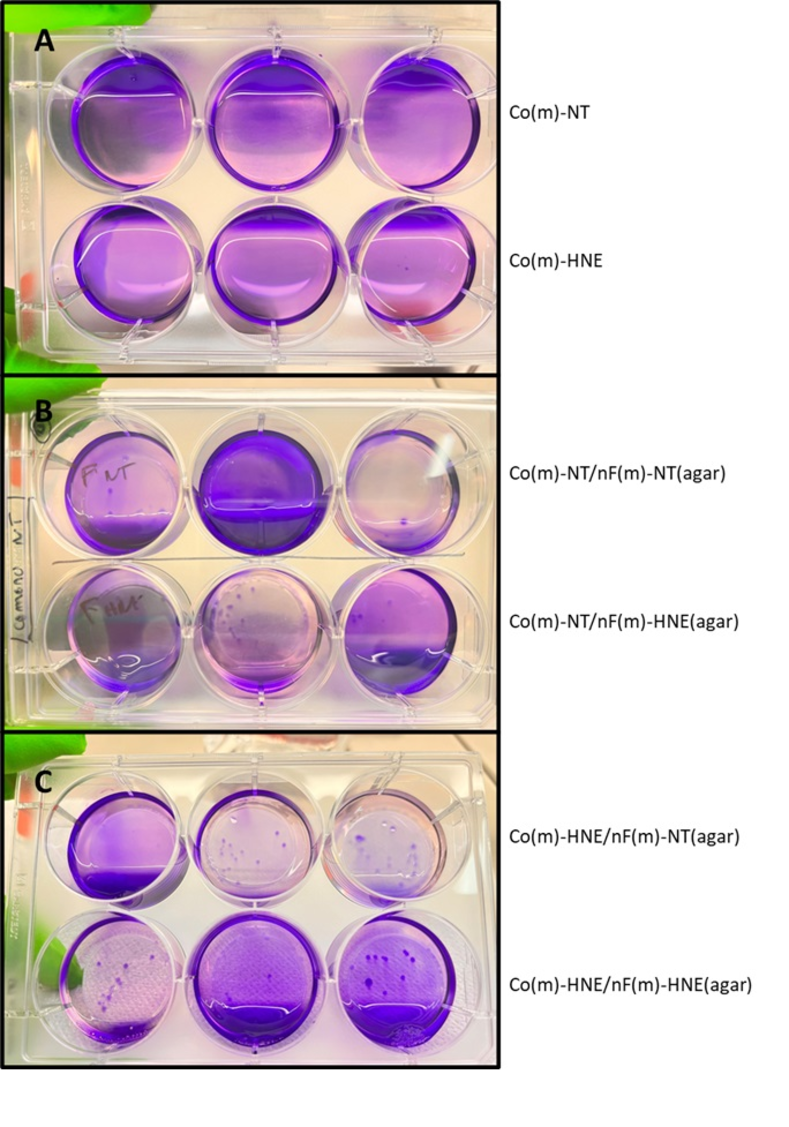

Supplement: S11 Fig — Co cells in a top layer of 0.35% agar medium were grown for 3 weeks without (A) or with (B and C) fibroblasts seeded onto Thincert™-Tissue culture inserts and placed on top of the agar wells. The wells were then stained with 0.1% crystal violet solution for 10 minutes at room temperature. The largest Co cell colonies were visible to the naked eye. (TIF) [file pone.0302932.s011.tif]
