# Supplementary material for: Epithelial-mesenchymal interaction protects normal colonocytes from 4-HNE-induced phenotypic transformation
Source: PLoS One. 2024 Apr 26;19(4):e0302932. doi: 10.1371/journal.pone.0302932 (PMC11051638; doi:10.1371/journal.pone.0302932)
Supplement: S1 Raw images — Raw blot data related to Tables 1 and 4 and S3 Table. (PDF) [file pone.0302932.s012.pdf]

Western blot Files

RAD51 (37 kDa)

Co cells

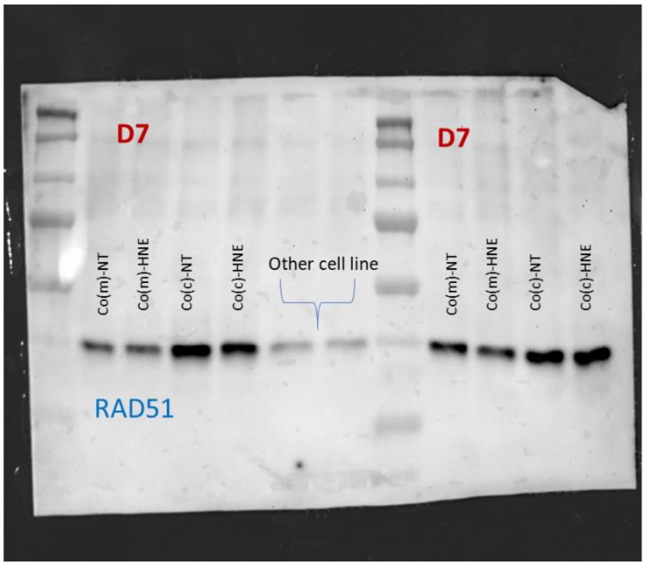

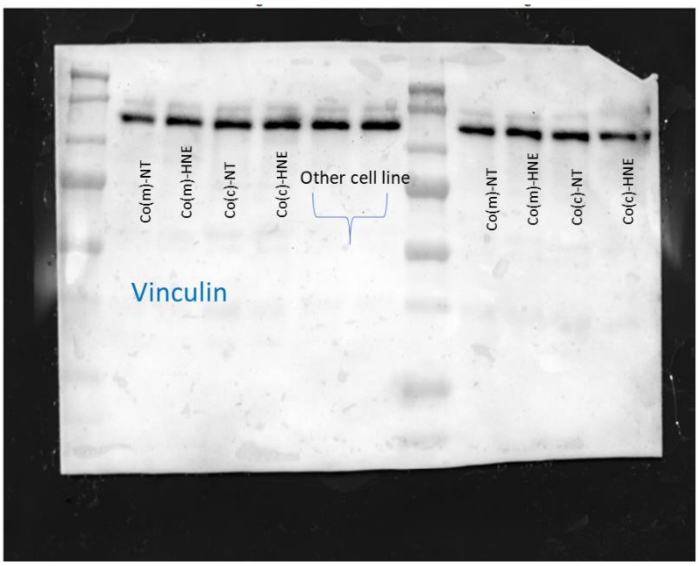

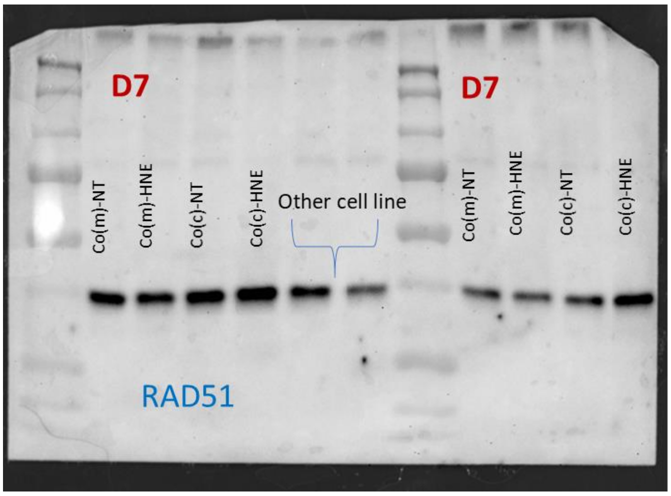

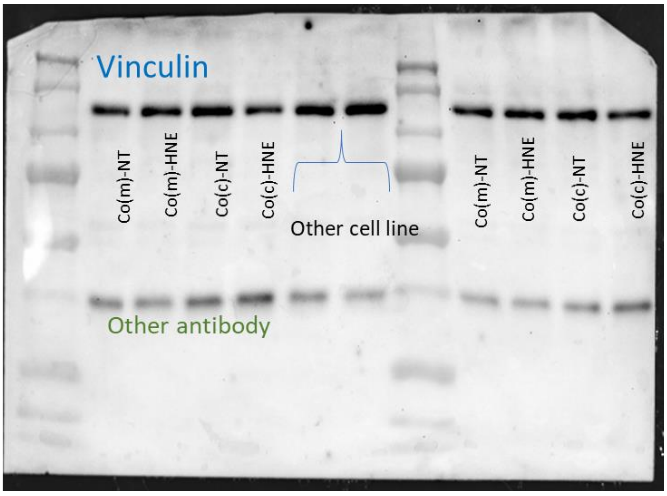

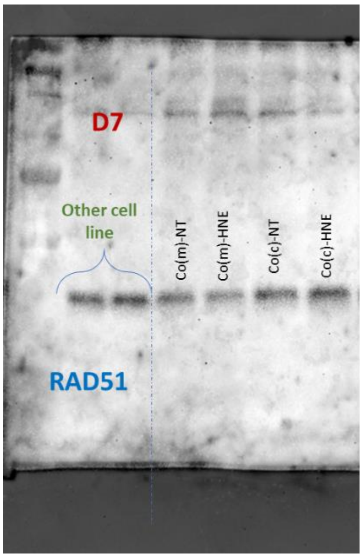
D7
Other cell line
Co(m)-NT
Co(m)-HNE
Co(c)-NT
Co(c)-HNE
RAD51

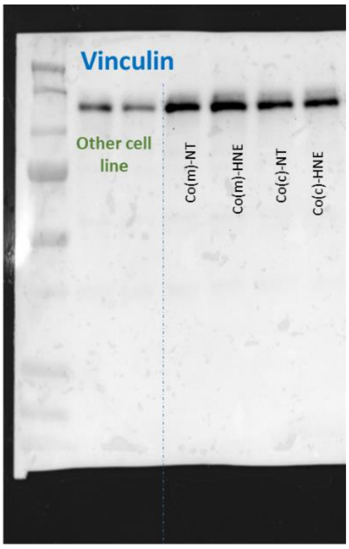
Vinculin
Other cell line
Co(m)-NT
Co(m)-HNE
Co(c)-NT
Co(c)-HNE

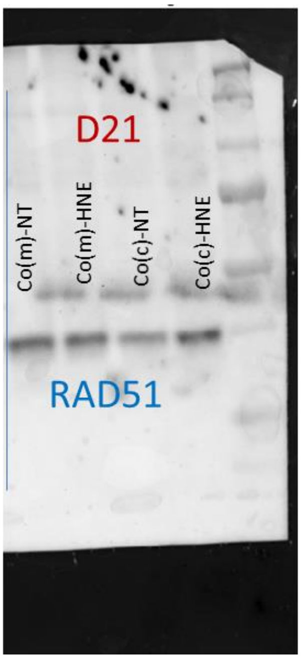
D21
Co(m)-NT
Co(m)-HNE
Co(c)-NT
Co(c)-HNE
RAD51

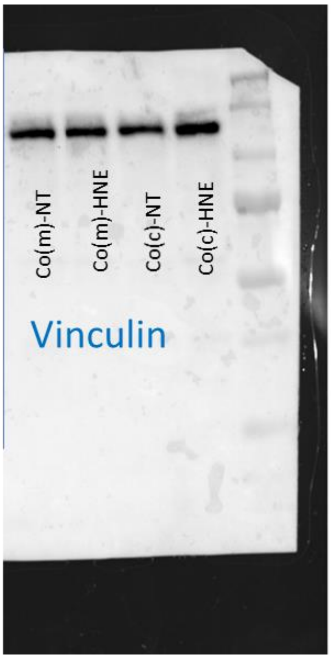
Co(m)-NT
Co(m)-HNE
Co(c)-NT
Co(c)-HNE
Vinculin

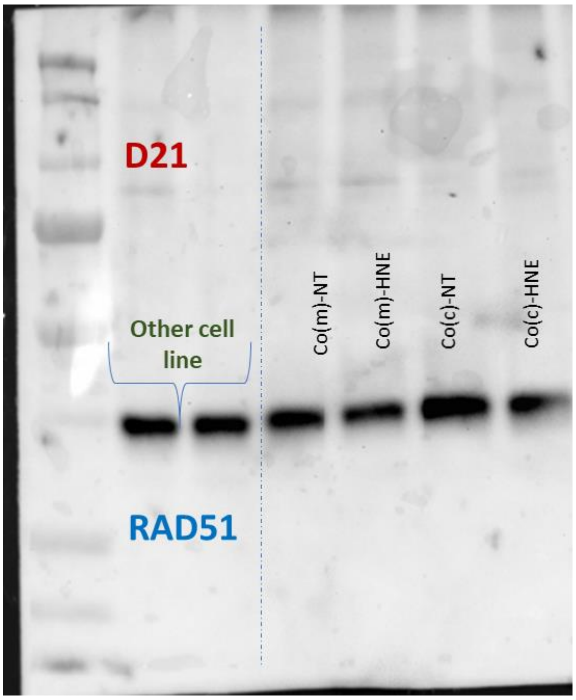
D21
Other cell line
Co(m)-NT
Co(m)-HNE
Co(c)-NT
Co(c)-HNE
RAD51

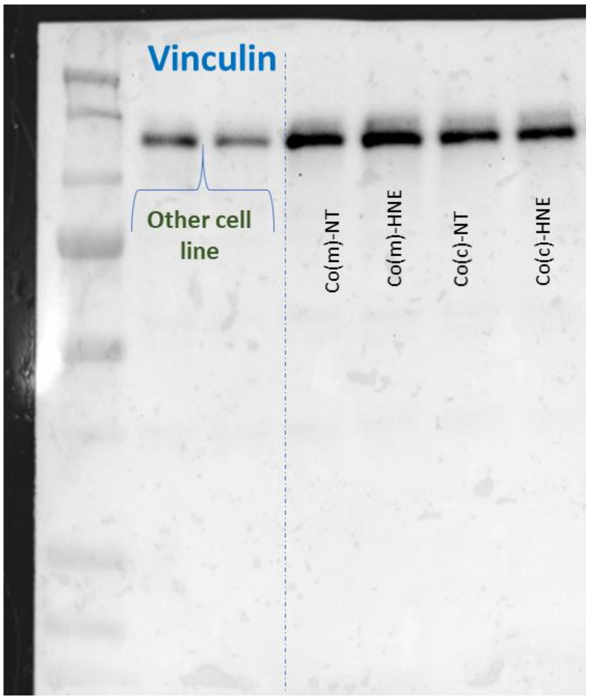
Vinculin
Other cell line
Co(m)-NT
Co(m)-HNE
Co(c)-NT
Co(c)-HNE

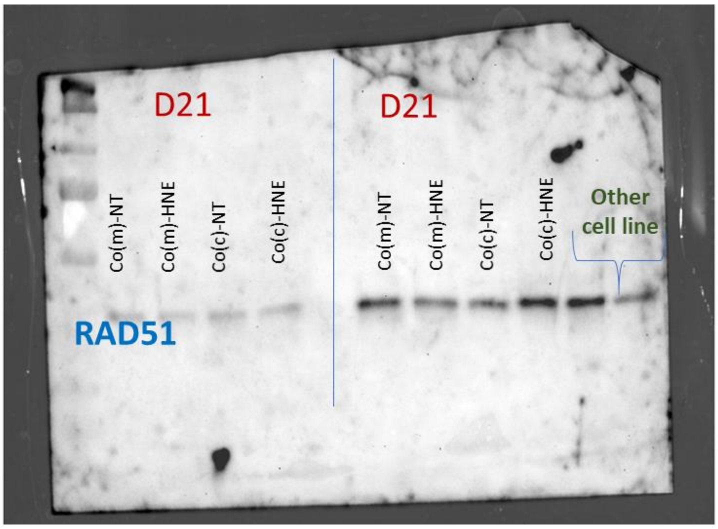
D21
D21
Co(m)-NT
Co(m)-HNE
Co(c)-NT
Co(c)-HNE
Co(m)-NT
Co(m)-HNE
Co(c)-NT
Co(c)-HNE
Other cell line
RAD51

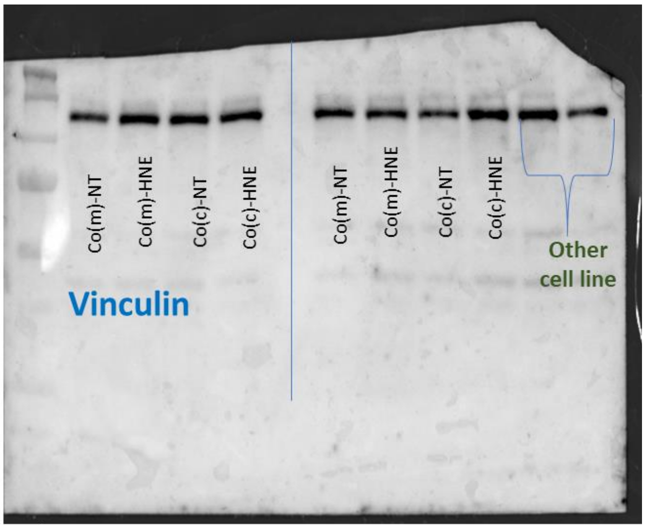
Co(m)-NT
Co(m)-HNE
Co(c)-NT
Co(c)-HNE
Co(m)-NT
Co(m)-HNE
Co(c)-NT
Co(c)-HNE
Other cell line
Vinculin

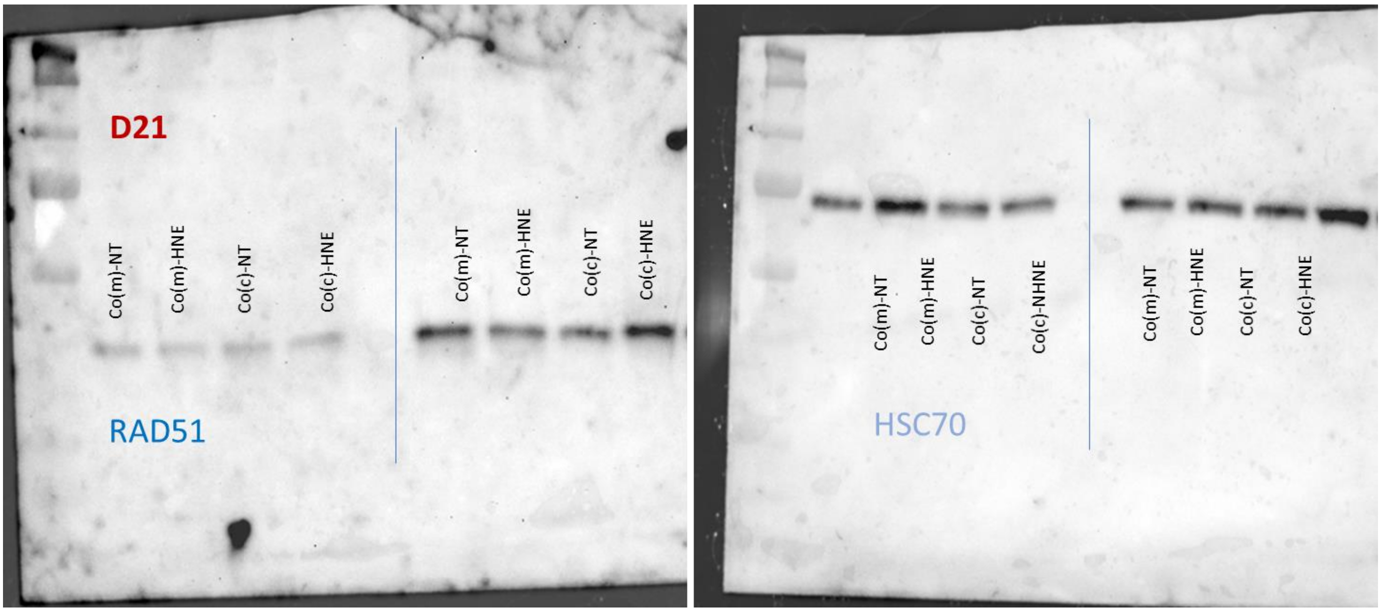
D21
Co(m)-NT
Co(m)-HNE
Co(c)-NT
Co(c)-HNE
Co(m)-NT
Co(m)-HNE
Co(c)-NT
Co(c)-HNE
RAD51
Co(m)-NT
Co(m)-HNE
Co(c)-NT
Co(c)-NHNE
Co(m)-NT
Co(m)-HNE
Co(c)-NT
Co(c)-HNE
HSC70

P21 (18 kDa)

Co cells

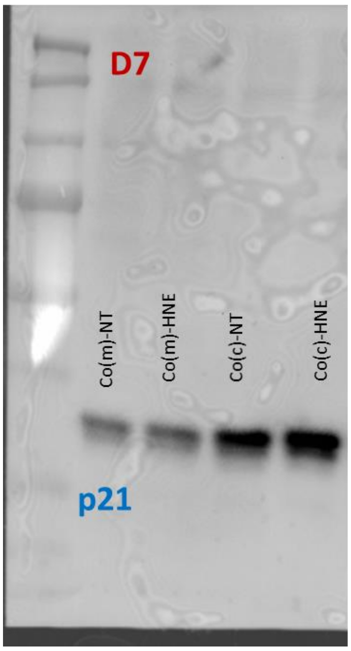

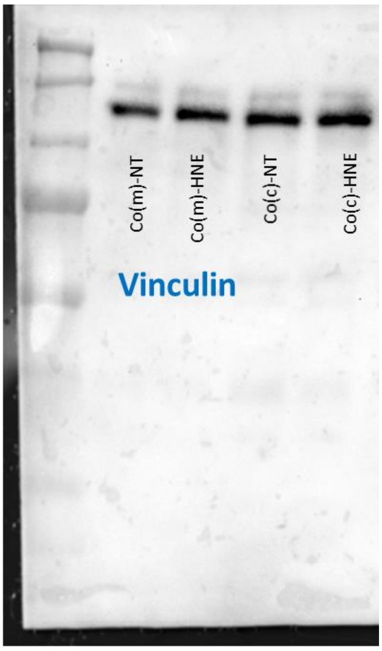

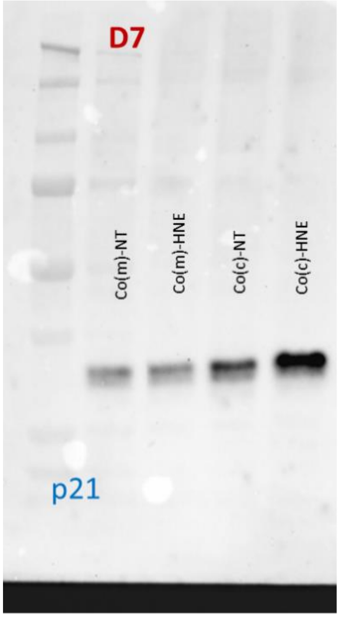

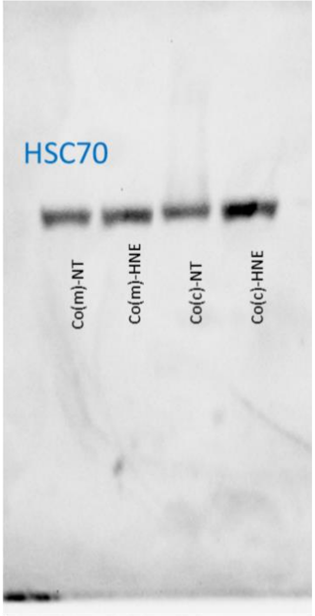

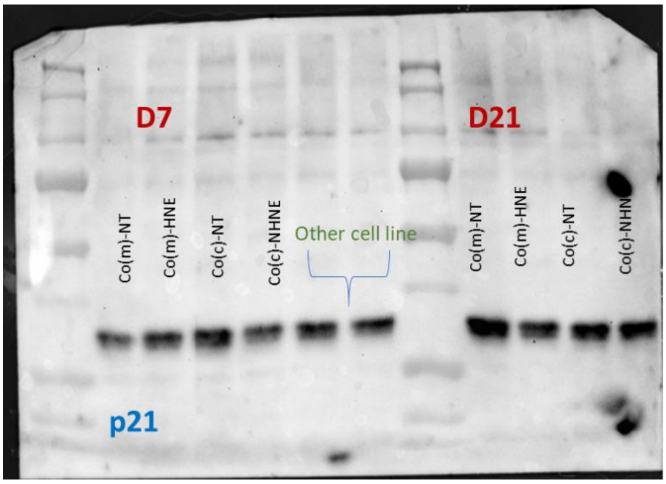
D7
D21
Co(m)-NT
Co(m)-HNE
Co(c)-NT
Co(c)-NHNE
Other cell line
Co(m)-NT
Co(m)-HNE
Co(c)-NT
Co(c)-NHNE
p21

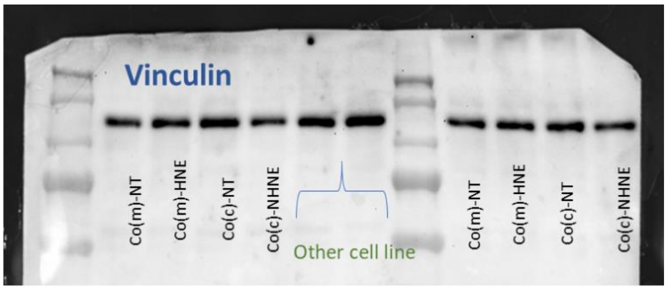
Vinculin
Co(m)-NT
Co(m)-HNE
Co(c)-NT
Co(c)-NHNE
Other cell line
Co(m)-NT
Co(m)-HNE
Co(c)-NT
Co(c)-NHNE

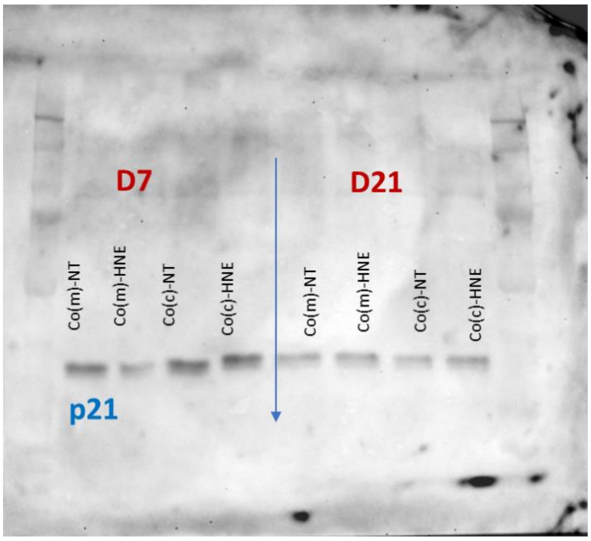
D7
D21
Co(m)-NT
Co(m)-HNE
Co(c)-NT
Co(c)-HNE
Co(m)-NT
Co(m)-HNE
Co(c)-NT
Co(c)-HNE
p21

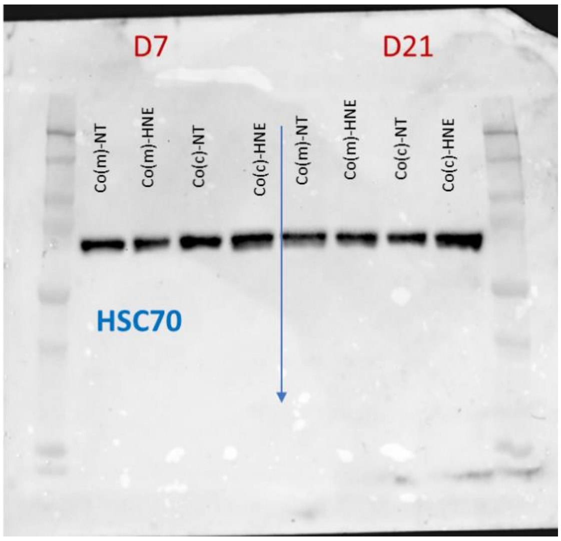
D7
D21
Co(m)-NT
Co(m)-HNE
Co(c)-NT
Co(c)-HNE
Co(m)-NT
Co(m)-HNE
Co(c)-NT
Co(c)-HNE
HSC70

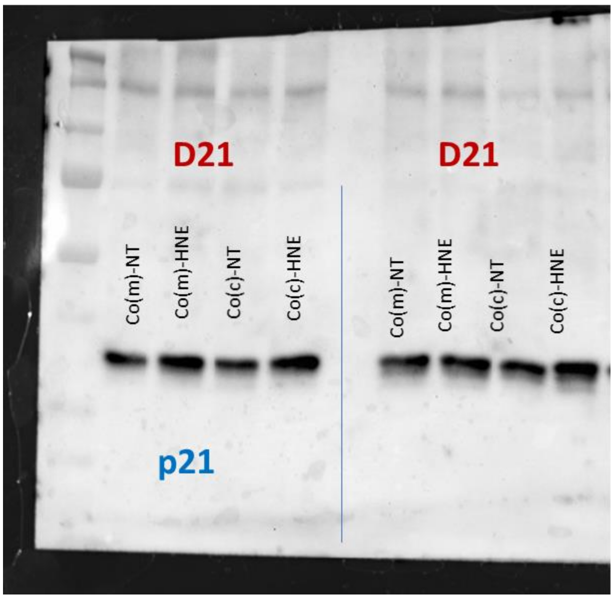
D21
D21
Co(m)-NT
Co(m)-HNE
Co(c)-NT
Co(c)-HNE
Co(m)-NT
Co(m)-HNE
Co(c)-NT
Co(c)-HNE
p21

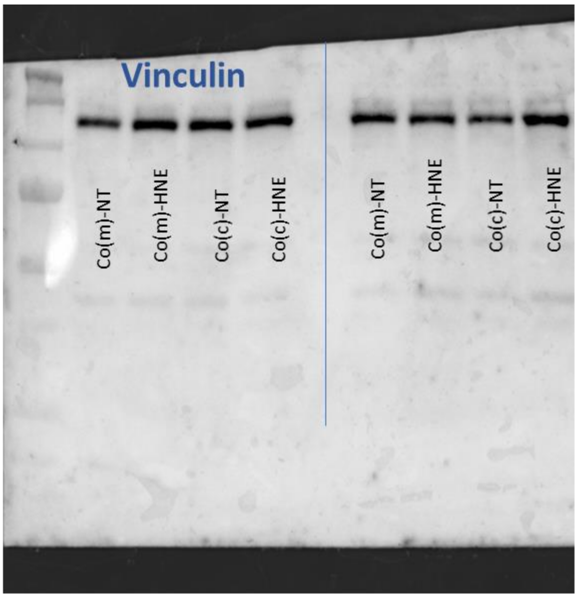
Vinculin
Co(m)-NT
Co(m)-HNE
Co(c)-NT
Co(c)-HNE
Co(m)-NT
Co(m)-HNE
Co(c)-NT
Co(c)-HNE

CYTOKERATIN 18 (CK18, 48 kDa)

Co cells

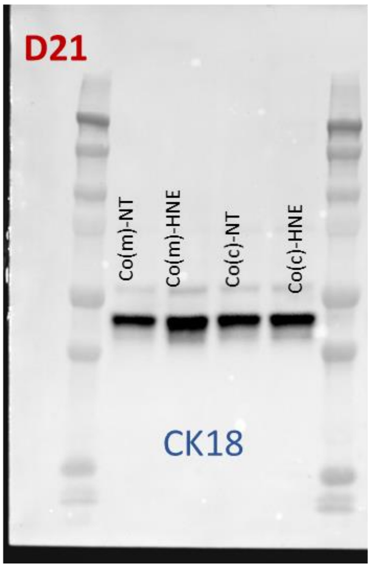

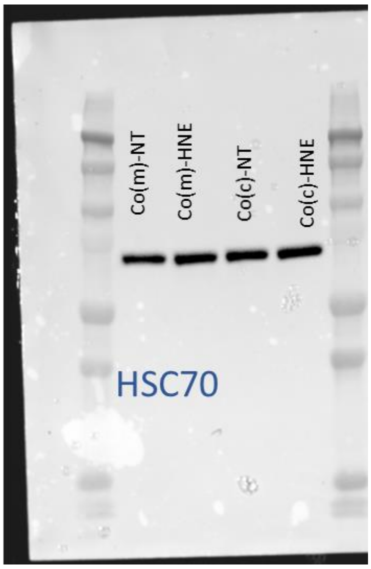

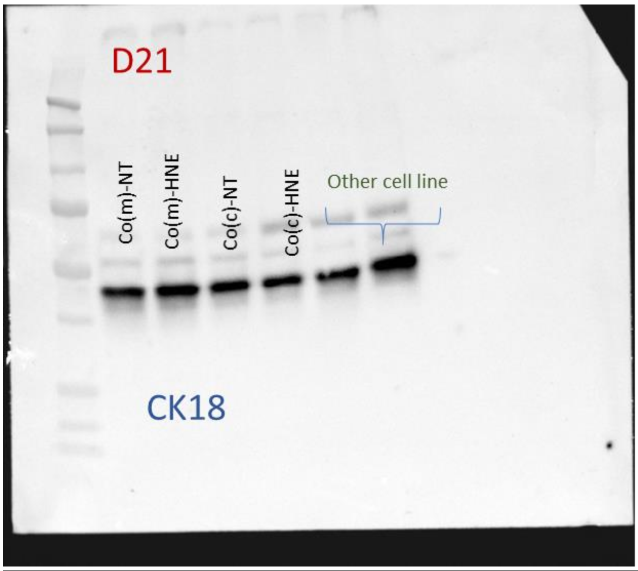

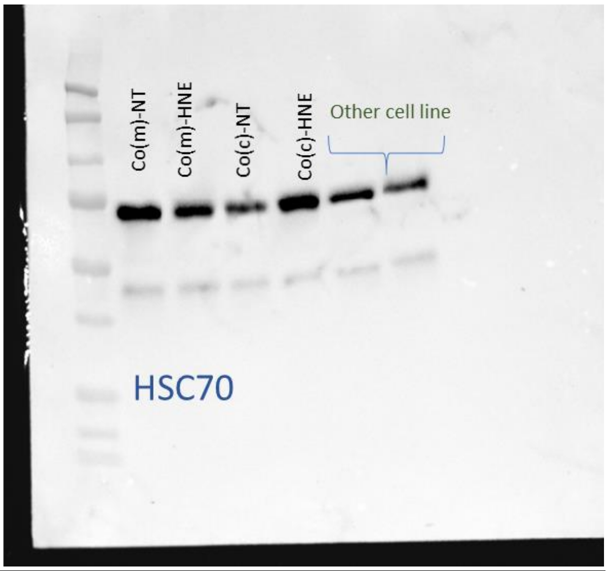

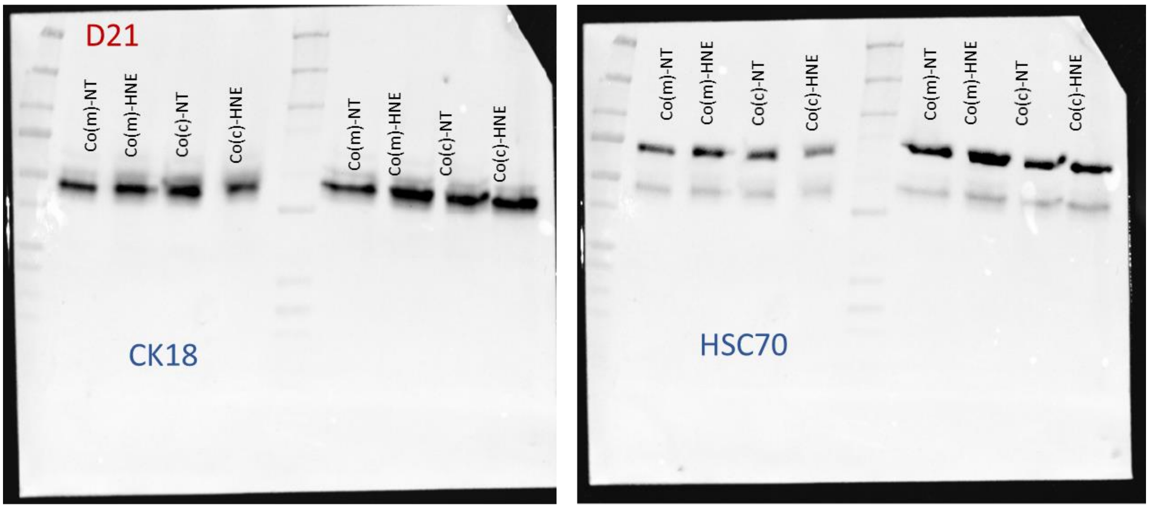
D21
Co(m)-NT
Co(m)-HNE
Co(c)-NT
Co(c)-HNE
Co(m)-NT
Co(m)-HNE
Co(c)-NT
Co(c)-HNE
CK18
Co(m)-NT
Co(m)-HNE
Co(c)-NT
Co(c)-HNE
Co(m)-NT
Co(m)-HNE
Co(c)-NT
Co(c)-HNE
HSC70

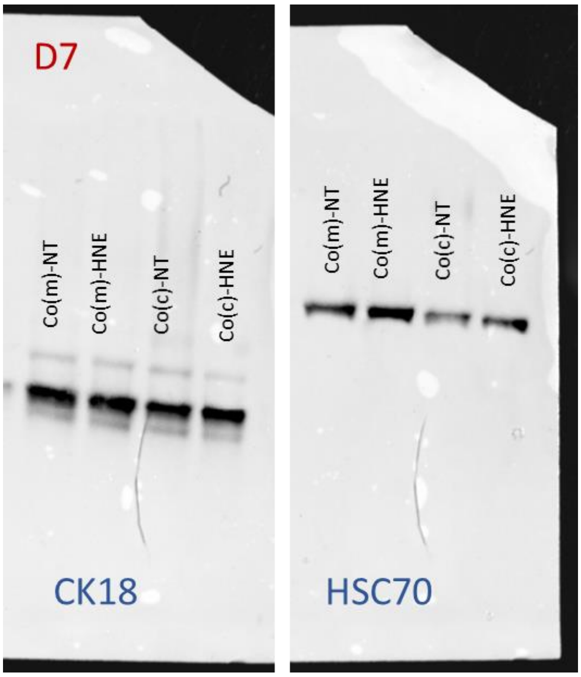
D7
Co(m)-NT
Co(m)-HNE
Co(c)-NT
Co(c)-HNE
CK18
Co(m)-NT
Co(m)-HNE
Co(c)-NT
Co(c)-HNE
HSC70

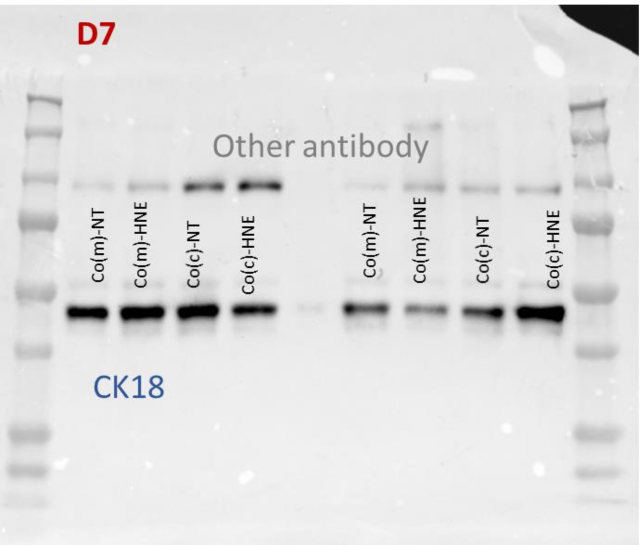
D7
Other antibody
Co(m)-NT
Co(m)-HNE
Co(c)-NT
Co(c)-HNE
Co(m)-NT
Co(m)-HNE
Co(c)-NT
Co(c)-HNE
CK18

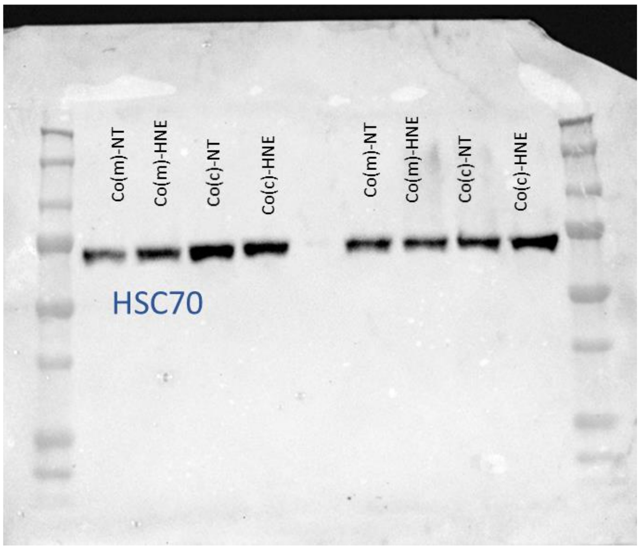
Co(m)-NT
Co(m)-HNE
Co(c)-NT
Co(c)-HNE
Co(m)-NT
Co(m)-HNE
Co(c)-NT
Co(c)-HNE
HSC70

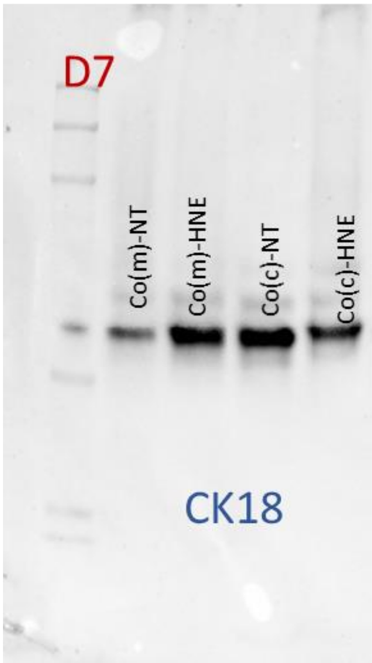
D7
Co(m)-NT
Co(m)-HNE
Co(c)-NT
Co(c)-HNE
CK18

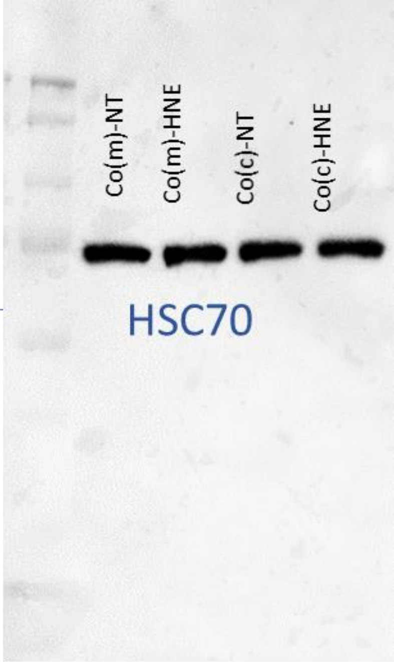
Co(m)-NT
Co(m)-HNE
Co(c)-NT
Co(c)-HNE
HSC70

Cox2 (74 kDa)

1/ Co cells

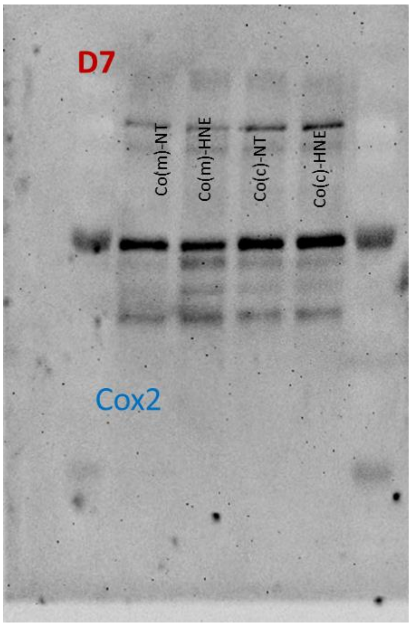

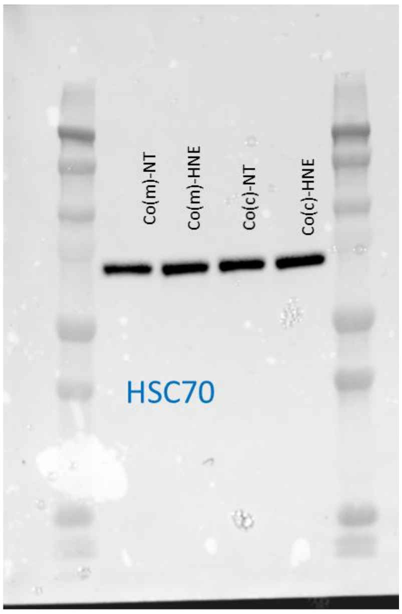

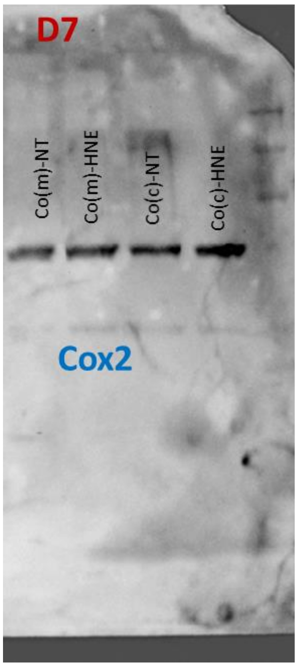

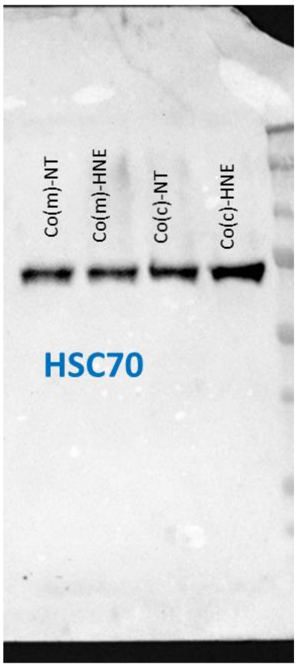

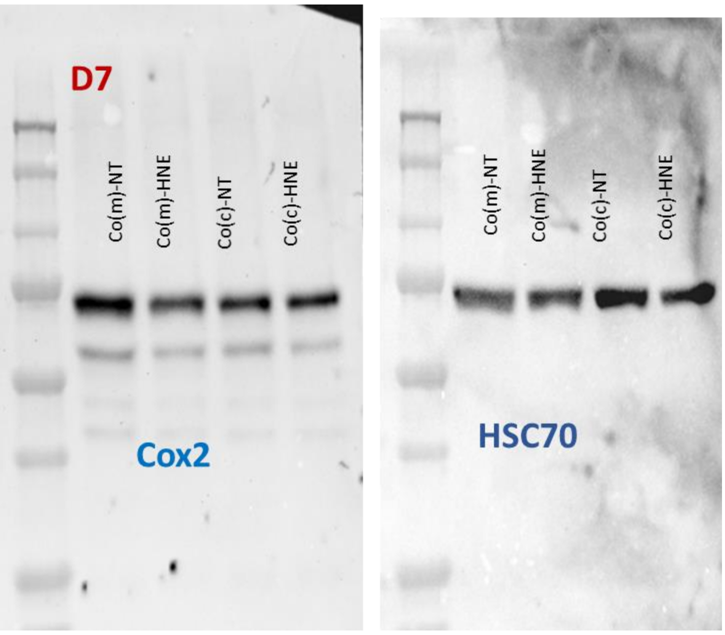
D7
Co(m)-NT
Co(m)-HNE
Co(c)-NT
Co(c)-HNE
Cox2
Co(m)-NT
Co(m)-HNE
Co(c)-NT
Co(c)-HNE
HSC70

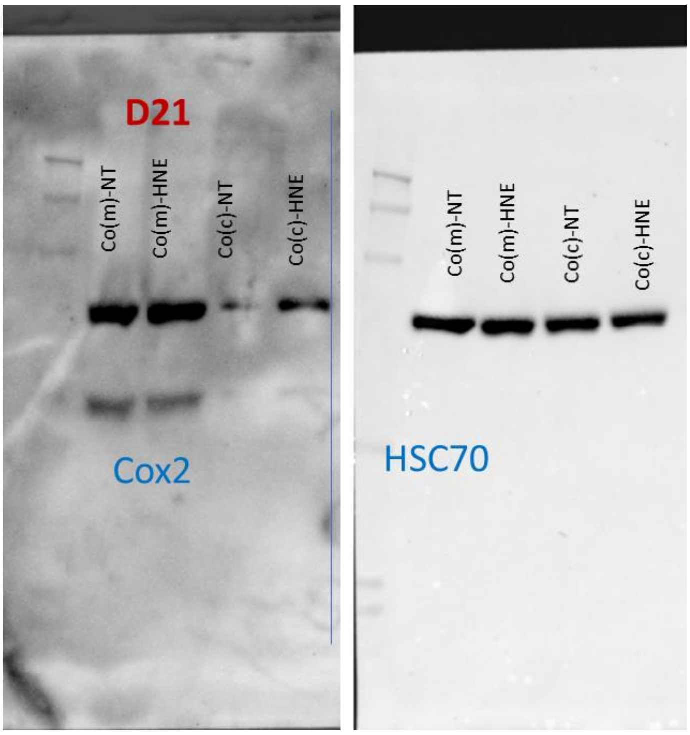
D21
Co(m)-NT
Co(m)-HNE
Co(c)-NT
Co(c)-HNE
Cox2
Co(m)-NT
Co(m)-HNE
Co(c)-NT
Co(c)-HNE
HSC70

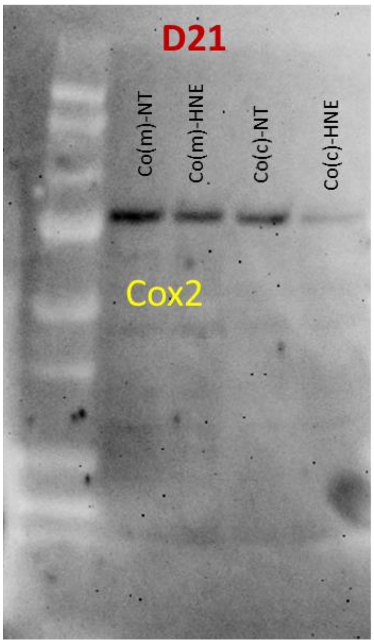
D21
Co(m)-NT
Co(m)-HNE
Co(c)-NT
Co(c)-HNE
Cox2

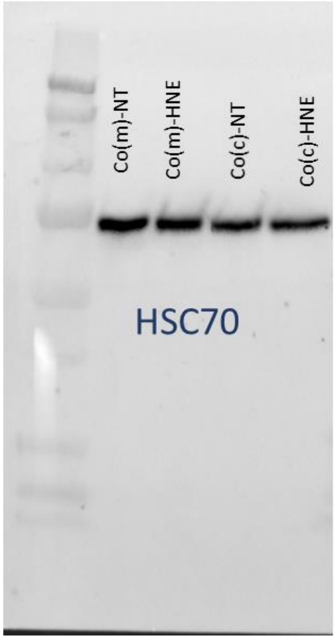
Co(m)-NT
Co(m)-HNE
Co(c)-NT
Co(c)-HNE
HSC70

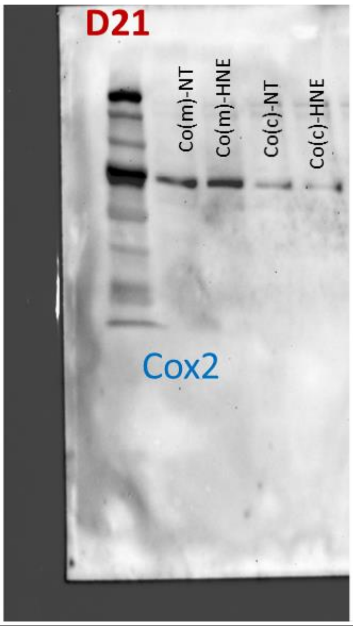
D21
Co(m)-NT
Co(m)-HNE
Co(c)-NT
Co(c)-HNE
Cox2

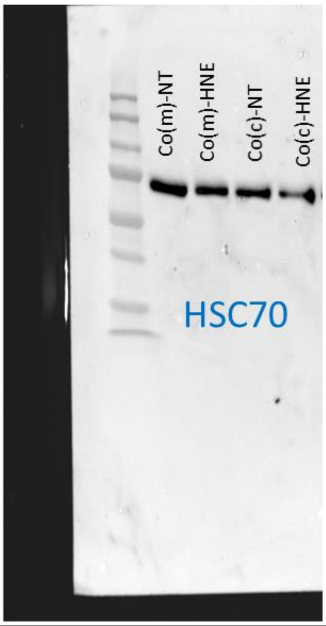
Co(m)-NT
Co(m)-HNE
Co(c)-NT
Co(c)-HNE
HSC70

2/ nF Cells

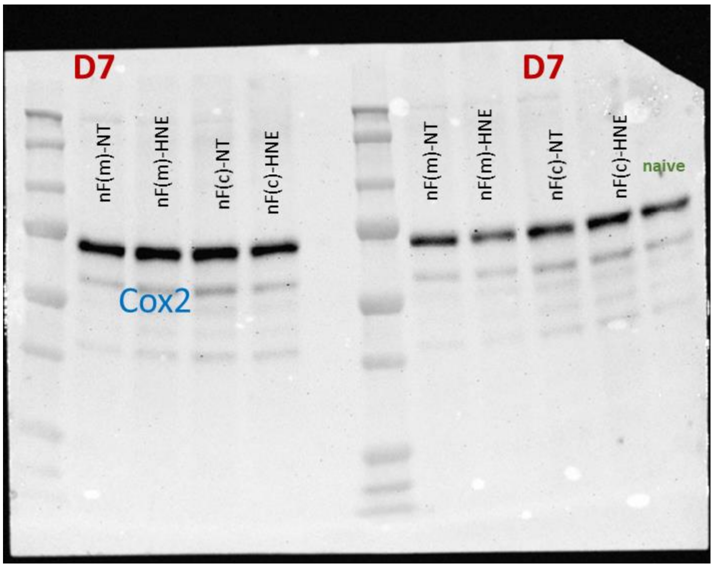
D7
nF(m)-NT
nF(m)-HNE
nF(c)-NT
nF(c)-HNE
Cox2
D7
nF(m)-NT
nF(m)-HNE
nF(c)-NT
nF(c)-HNE
naive

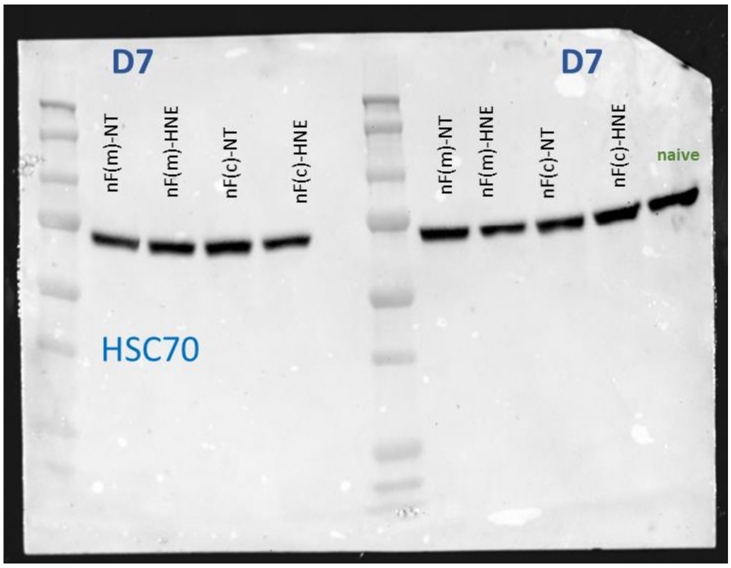
D7
nF(m)-NT
nF(m)-HNE
nF(c)-NT
nF(c)-HNE
HSC70
D7
nF(m)-NT
nF(m)-HNE
nF(c)-NT
nF(c)-HNE
naive

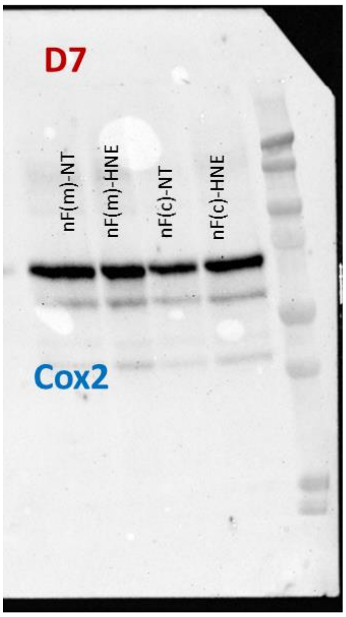
D7
nF(m)-NT
nF(m)-HNE
nF(c)-NT
nF(c)-HNE
Cox2

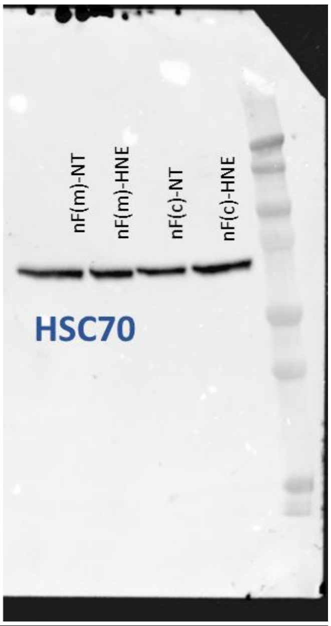
nF(m)-NT
nF(m)-HNE
nF(c)-NT
nF(c)-HNE
HSC70

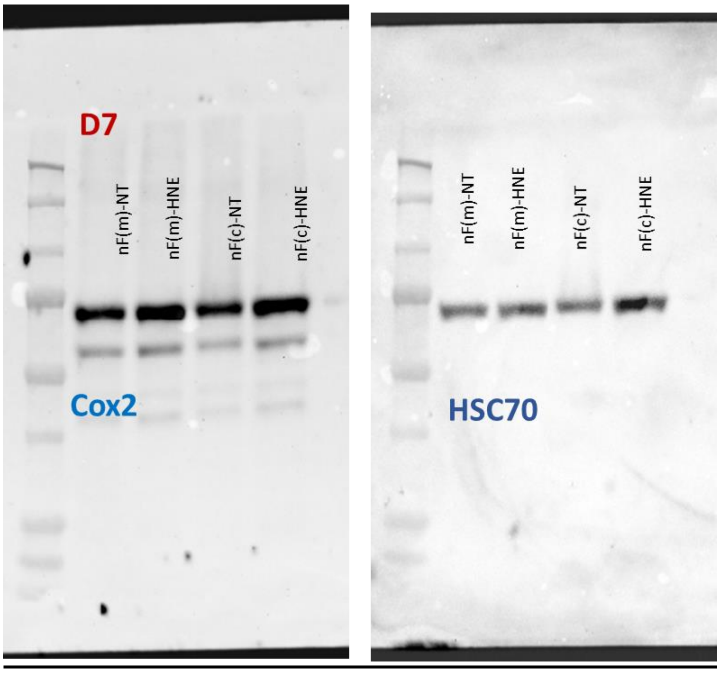
D7
nF(m)-NT
nF(m)-HNE
nF(c)-NT
nF(c)-HNE
Cox2
nF(m)-NT
nF(m)-HNE
nF(c)-NT
nF(c)-HNE
HSC70

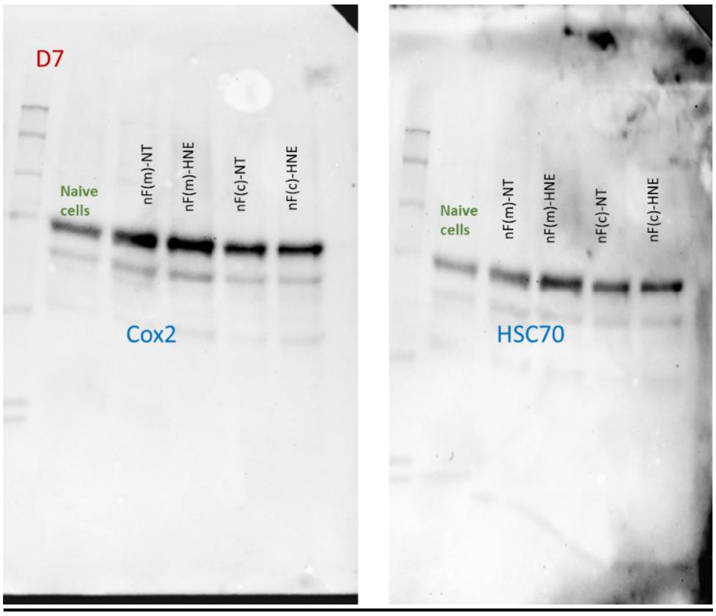
D7
Naive cells
nF(m)-NT
nF(m)-HNE
nF(c)-NT
nF(c)-HNE
Cox2
Naive cells
nF(m)-NT
nF(m)-HNE
nF(c)-NT
nF(c)-HNE
HSC70

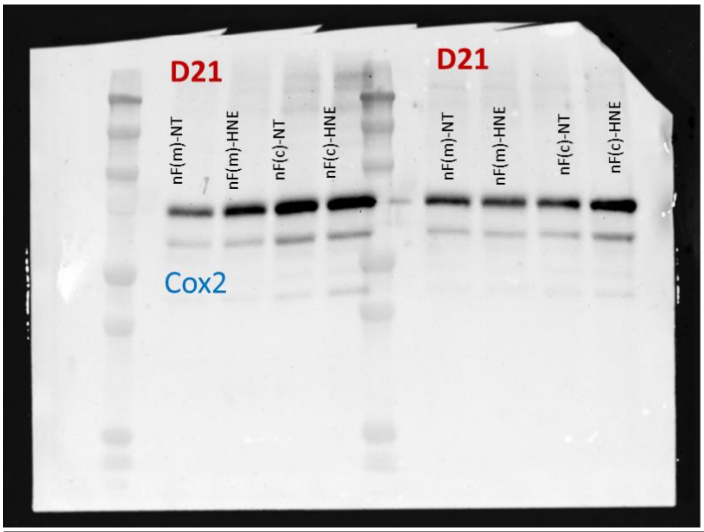
D21
nF(m)-NT
nF(m)-HNE
nF(c)-NT
nF(c)-HNE
D21
nF(m)-NT
nF(m)-HNE
nF(c)-NT
nF(c)-HNE
Cox2

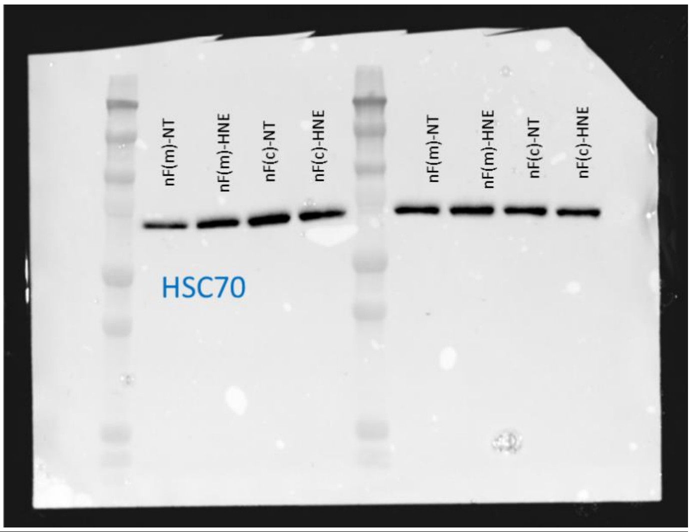
nF(m)-NT
nF(m)-HNE
nF(c)-NT
nF(c)-HNE
nF(m)-NT
nF(m)-HNE
nF(c)-NT
nF(c)-HNE
HSC70

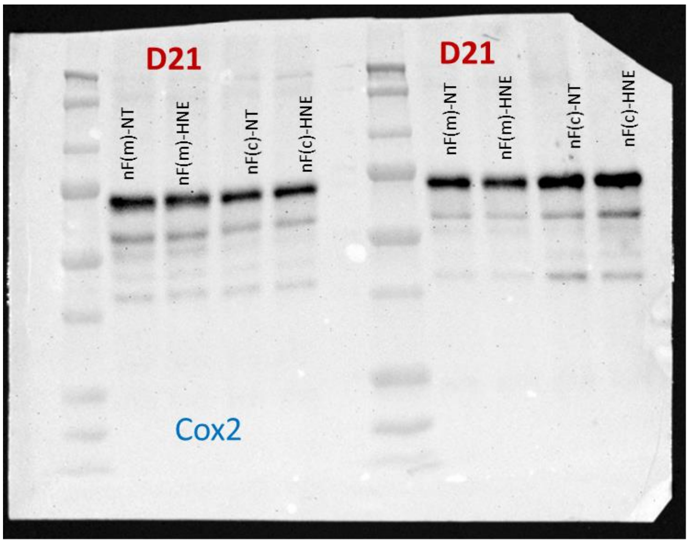
D21
nF(m)-NT
nF(m)-HNE
nF(c)-NT
nF(c)-HNE
D21
nF(m)-NT
nF(m)-HNE
nF(c)-NT
nF(c)-HNE
Cox2

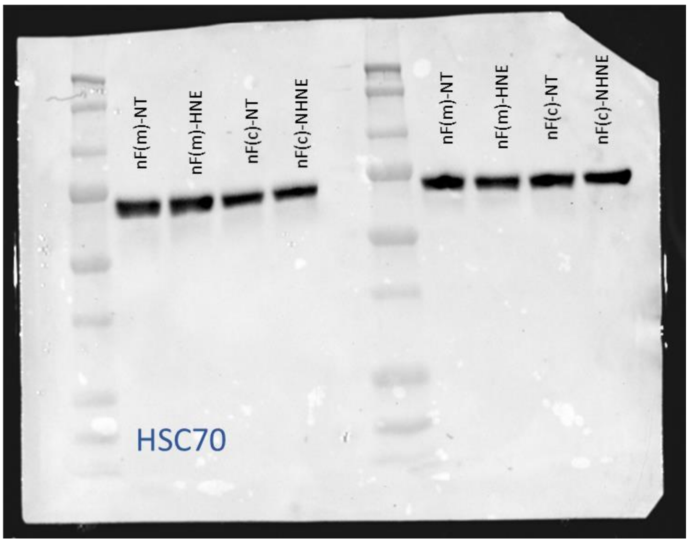
nF(m)-NT
nF(m)-HNE
nF(c)-NT
nF(c)-NHNE
nF(m)-NT
nF(m)-HNE
nF(c)-NT
nF(c)-NHNE
HSC70

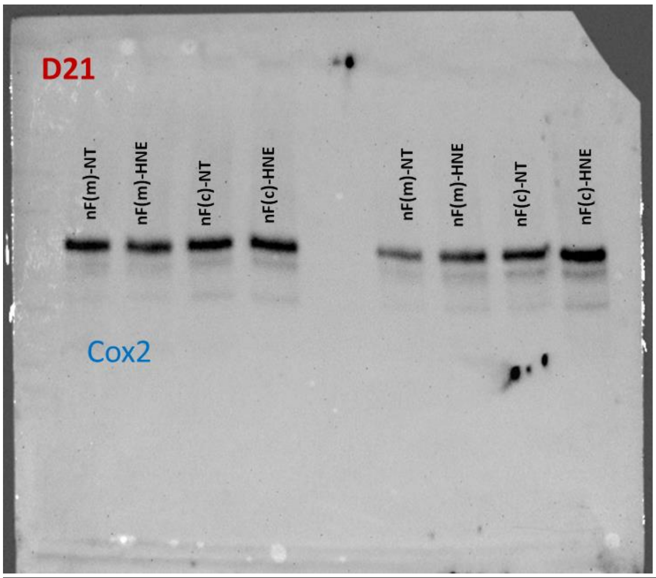
D21
nF(m)-NT
nF(m)-HNE
nF(c)-NT
nF(c)-HNE
nF(m)-NT
nF(m)-HNE
nF(c)-NT
nF(c)-HNE
Cox2

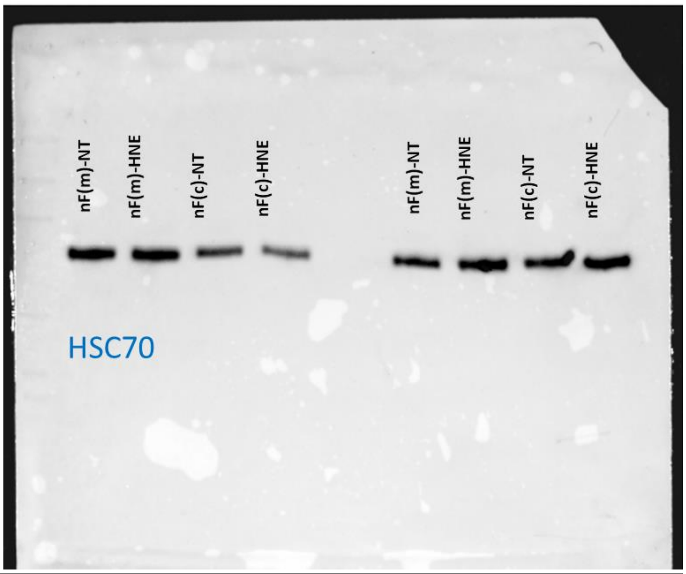
nF(m)-NT
nF(m)-HNE
nF(c)-NT
nF(c)-HNE
nF(m)-NT
nF(m)-HNE
nF(c)-NT
nF(c)-HNE
HSC70

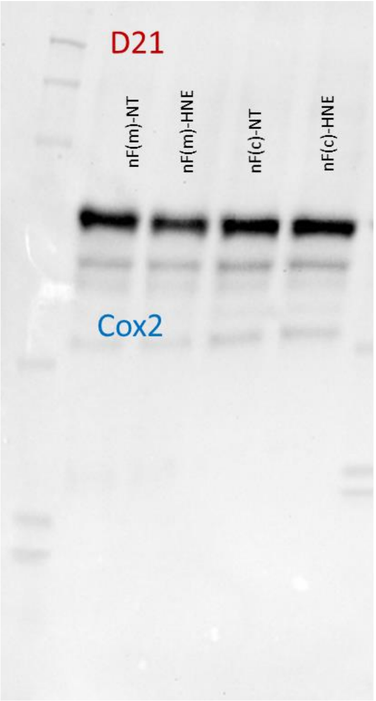
D21
nF(m)-NT
nF(m)-HNE
nF(c)-NT
nF(c)-HNE
Cox2

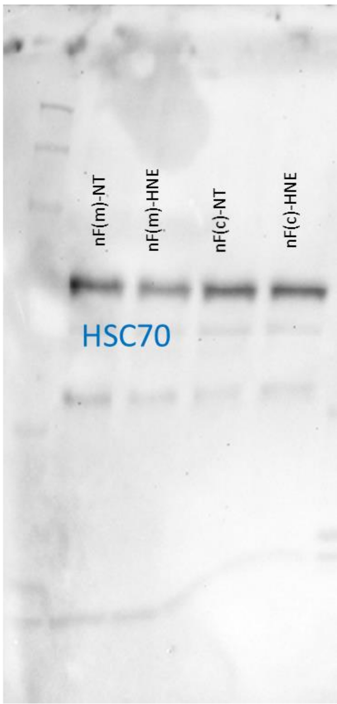
nF(m)-NT
nF(m)-HNE
nF(c)-NT
nF(c)-HNE
HSC70

αSMA (42 kDa)

<u>**nF cells**</u>

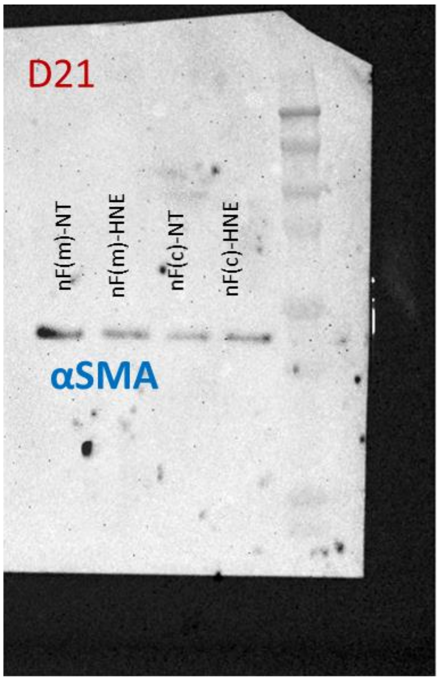

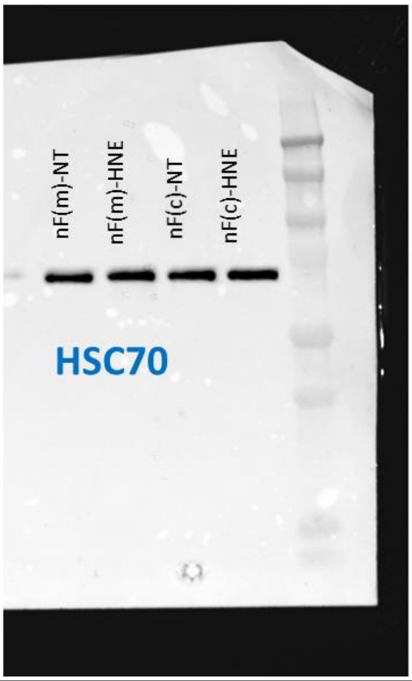

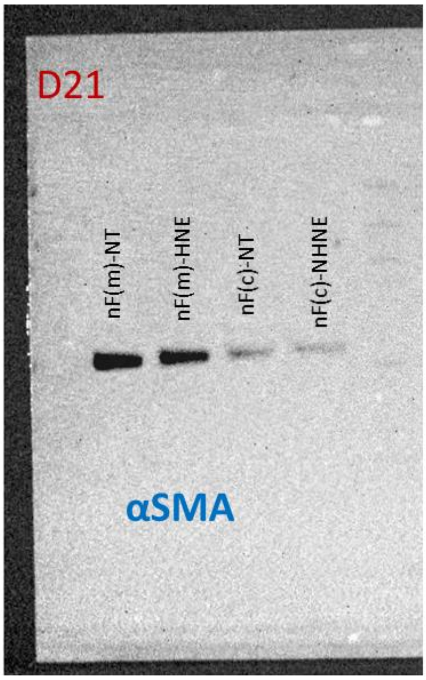

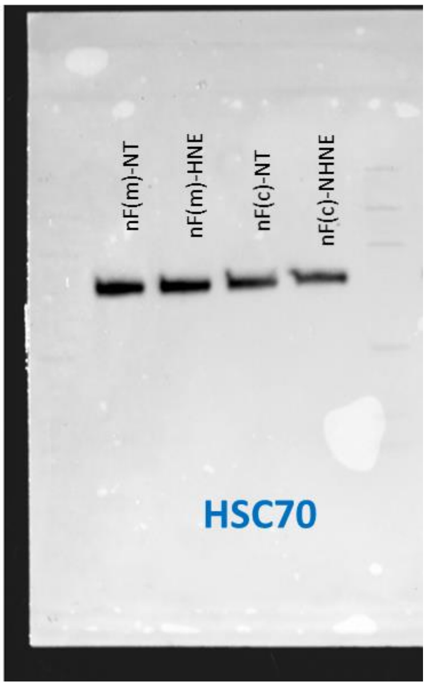

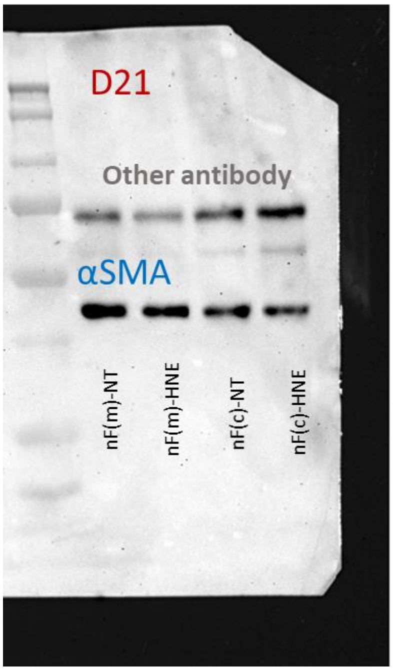
D21
Other antibody
αSMA
nF(m)-NT
nF(m)-HNE
nF(c)-NT
nF(c)-HNE

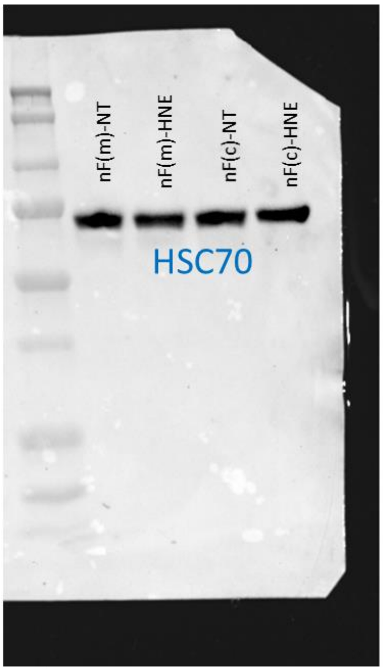
nF(m)-NT
nF(m)-HNE
nF(c)-NT
nF(c)-HNE
HSC70

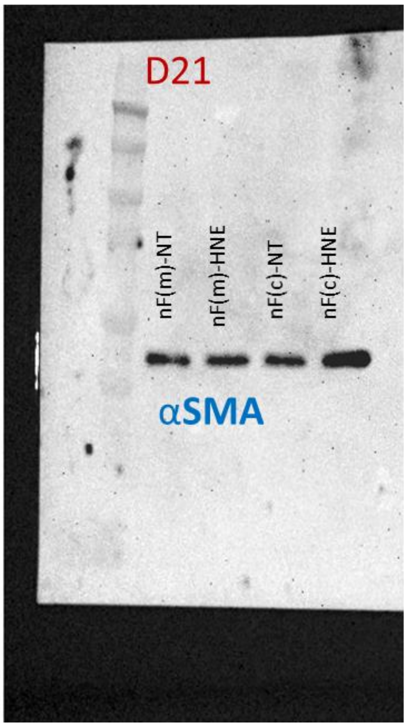
D21
nF(m)-NT
nF(m)-HNE
nF(c)-NT
nF(c)-HNE
αSMA

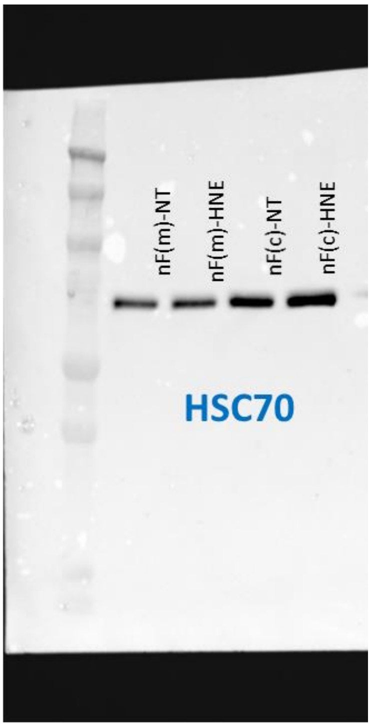
nF(m)-NT
nF(m)-HNE
nF(c)-NT
nF(c)-HNE
HSC70

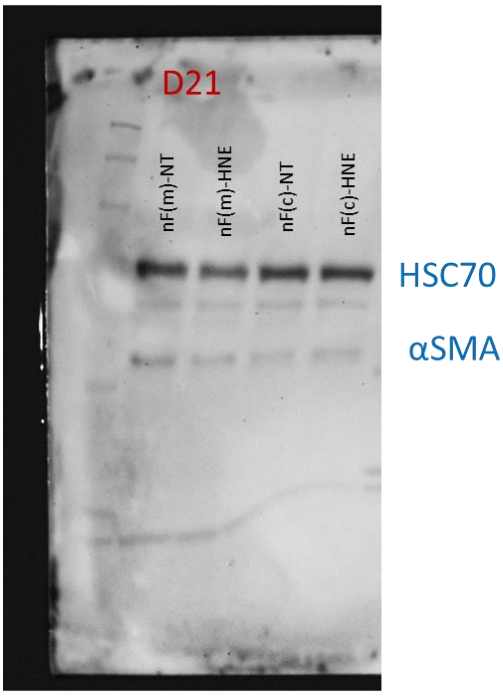
D21
nF(m)-NT
nF(m)-HNE
nF(c)-NT
nF(c)-HNE
HSC70
αSMA

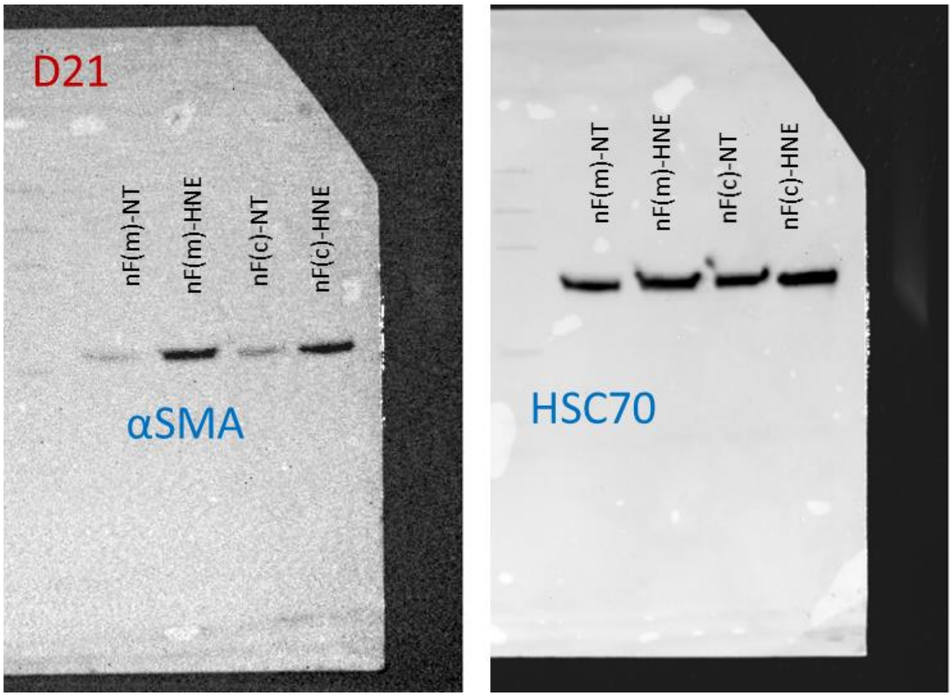
D21
nF(m)-NT
nF(m)-HNE
nF(c)-NT
nF(c)-HNE
αSMA
nF(m)-NT
nF(m)-HNE
nF(c)-NT
nF(c)-HNE
HSC70

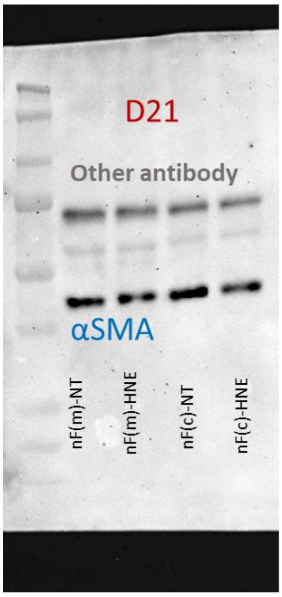
D21
Other antibody
αSMA
nF(m)-NT
nF(m)-HNE
nF(c)-NT
nF(c)-HNE

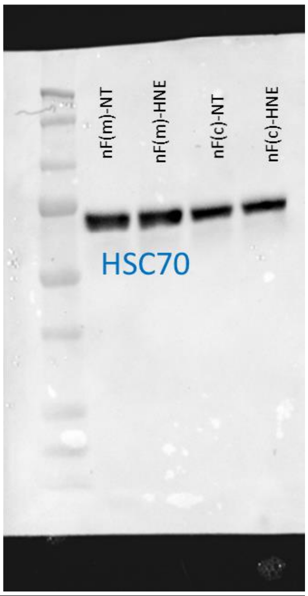
nF(m)-NT
nF(m)-HNE
nF(c)-NT
nF(c)-HNE
HSC70

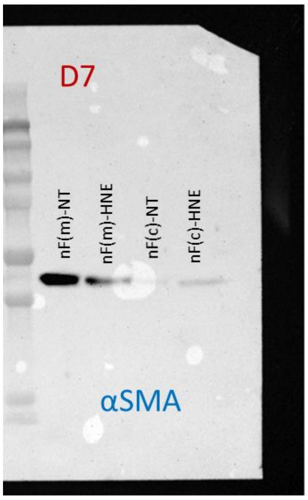
D7
nF(m)-NT
nF(m)-HNE
nF(c)-NT
nF(c)-HNE
αSMA

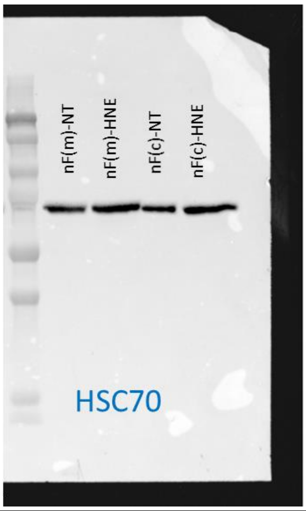
nF(m)-NT
nF(m)-HNE
nF(c)-NT
nF(c)-HNE
HSC70

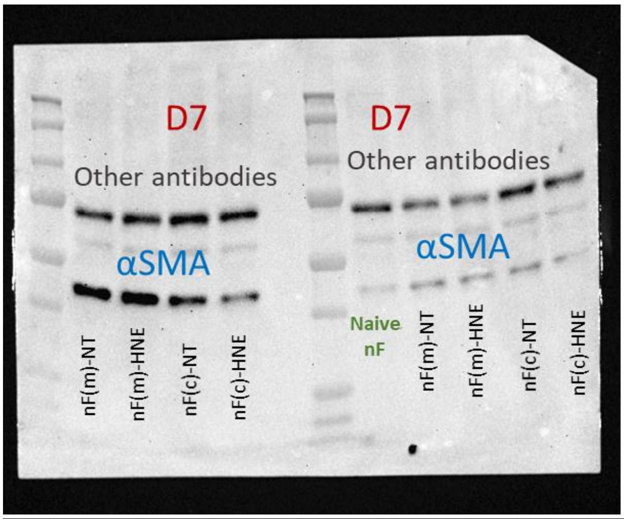
D7
Other antibodies
αSMA
nF(m)-NT
nF(m)-HNE
nF(c)-NT
nF(c)-HNE
D7
Other antibodies
αSMA
Naive nF
nF(m)-NT
nF(m)-HNE
nF(c)-NT
nF(c)-HNE

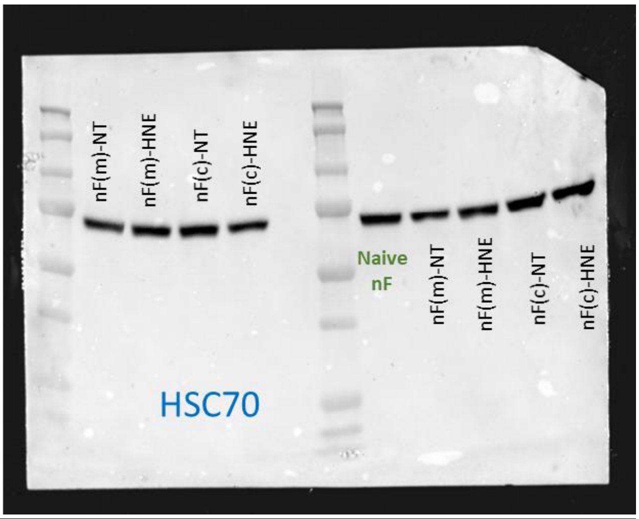
nF(m)-NT
nF(m)-HNE
nF(c)-NT
nF(c)-HNE
Naive nF
nF(m)-NT
nF(m)-HNE
nF(c)-NT
nF(c)-HNE
HSC70

Vimentin (55 kDa)

nF cells

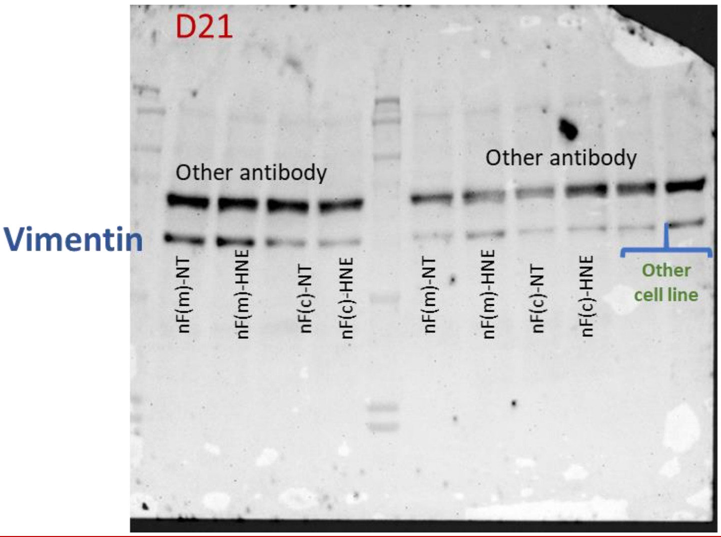

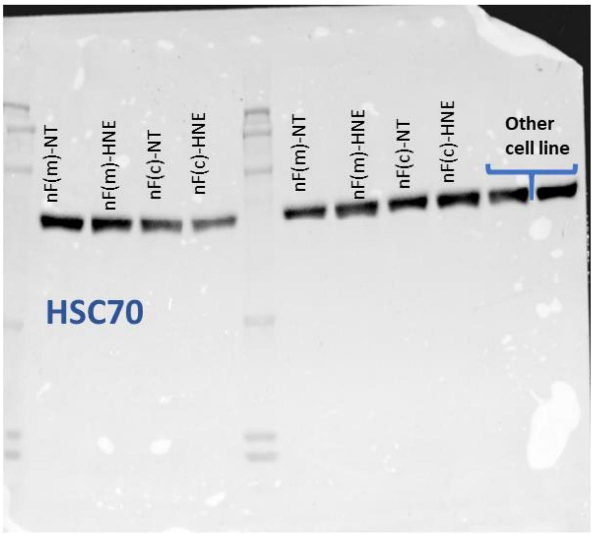

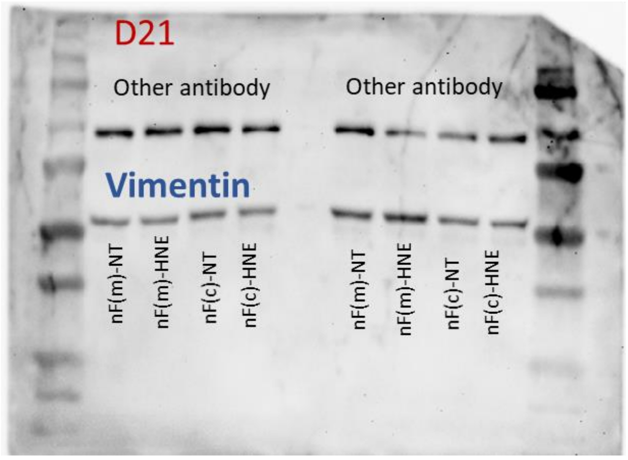

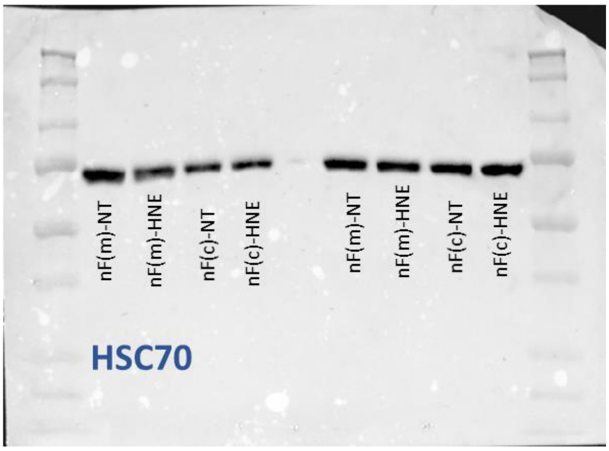

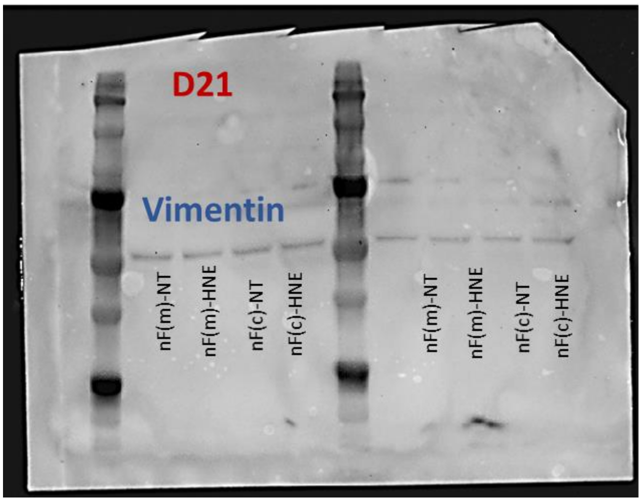
D21
Vimentin
nF(m)-NT
nF(m)-HNE
nF(c)-NT
nF(c)-HNE
nF(m)-NT
nF(m)-HNE
nF(c)-NT
nF(c)-HNE

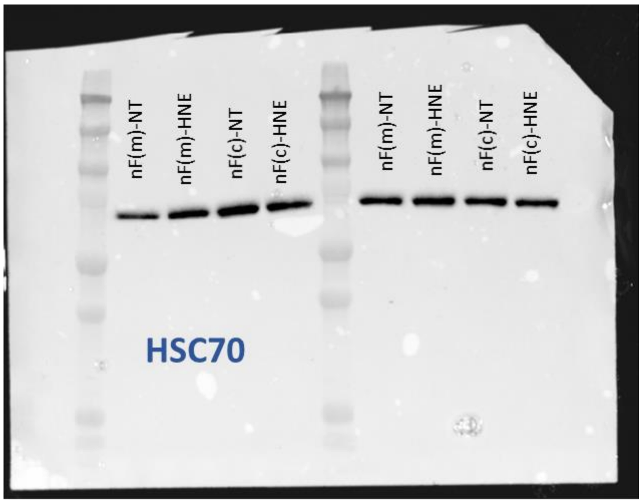
nF(m)-NT
nF(m)-HNE
nF(c)-NT
nF(c)-HNE
nF(m)-NT
nF(m)-HNE
nF(c)-NT
nF(c)-HNE
HSC70

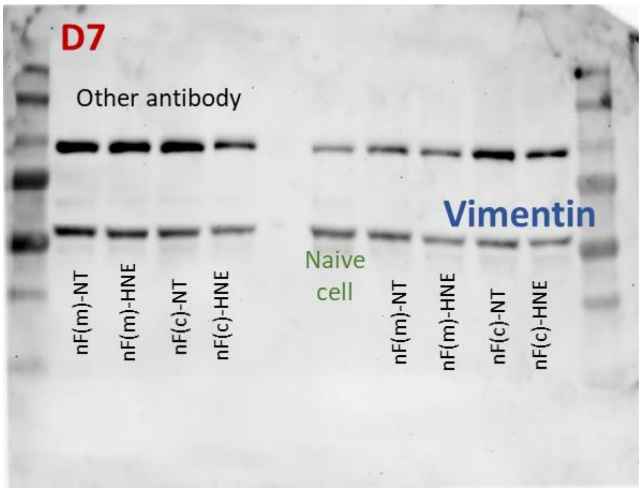
D7
Other antibody
Vimentin
Naive cell
nF(m)-NT
nF(m)-HNE
nF(c)-NT
nF(c)-HNE
nF(m)-NT
nF(m)-HNE
nF(c)-NT
nF(c)-HNE

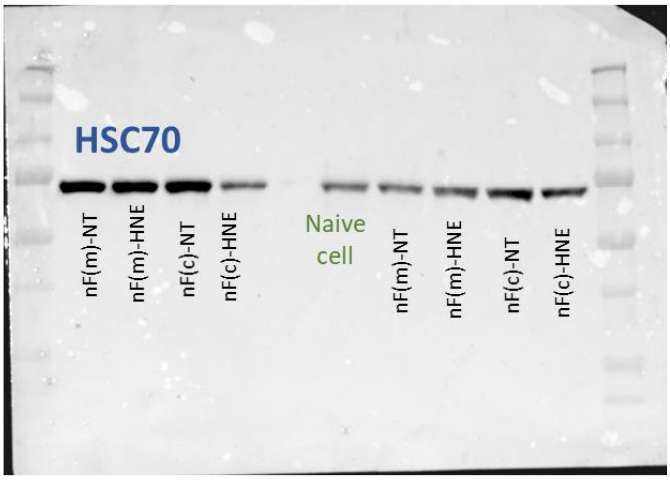
HSC70
Naive cell
nF(m)-NT
nF(m)-HNE
nF(c)-NT
nF(c)-HNE
nF(m)-NT
nF(m)-HNE
nF(c)-NT
nF(c)-HNE

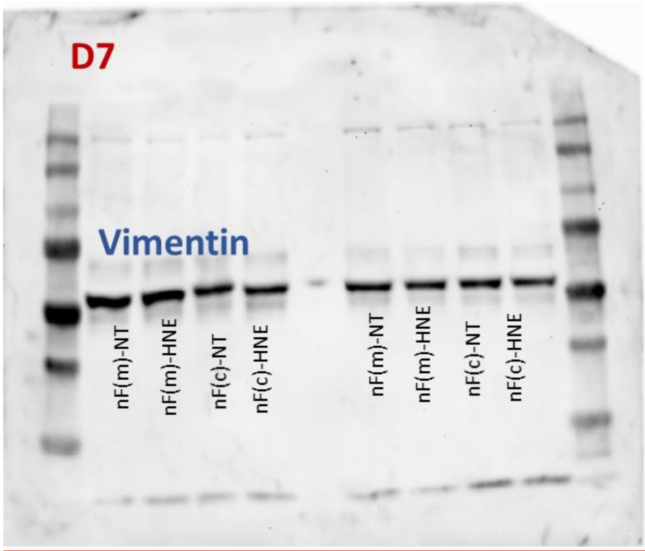
D7
Vimentin
nF(m)-NT
nF(m)-HNE
nF(c)-NT
nF(c)-HNE
nF(m)-NT
nF(m)-HNE
nF(c)-NT
nF(c)-HNE

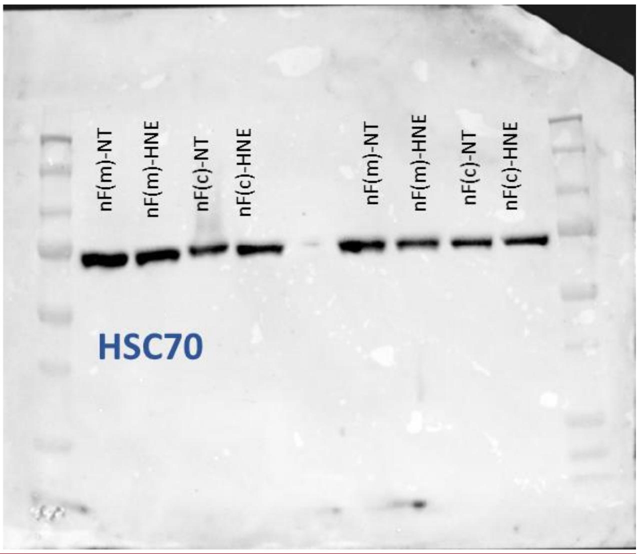
nF(m)-NT
nF(m)-HNE
nF(c)-NT
nF(c)-HNE
nF(m)-NT
nF(m)-HNE
nF(c)-NT
nF(c)-HNE
HSC70
