# Supplementary material for: Epithelial-mesenchymal interaction protects normal colonocytes from 4-HNE-induced phenotypic transformation
Source: PLoS One. 2024 Apr 26;19(4):e0302932. doi: 10.1371/journal.pone.0302932 (PMC11051638; doi:10.1371/journal.pone.0302932)
Supplement: S1 Table — (DOCX) [file pone.0302932.s013.docx]

| **Gene** | **Alias** | **sequence primer Forward** | **sequence primer Reverse** |
| --- | --- | --- | --- |
|  |  |  |  |
| **Hprt1** | Hypoxanthine Phosphoribosyltransferase 1 | GCCGACCCGCAGTCC | TCATAACCTGGTTCATCATCGC |
| **Apc** | Adenomatosis Polyposis Coli Tumor Suppressor | GTGGACTGTGAGATGTATGGGC | CACAAGTGCTCTCATGCAGCCT |
| **Ctnnb1** | Catenin Beta 1 | TCACATTTGAGAAGCGATCCTAC | TCCAGCTCGGATTCCATGAAC |
| **Wnt2b** | Wnt Family Member 2B | GCACGGCTGTTCGGAGATT | ACTCACACCGTGACACTTGC |
| **Wnt5a** | Wnt Family Member 5a | CACAGTGGACAATACTTCTGTCTTTG | CGTCTCTCGGCTGCCTATTTG |
| **Prom1** | Prominin 1 = cd133 | CAACTAGAAGAGGCTGTGTG | CCCAGCCATGAGGAAGA |
| **Cd44** | CD44 Molecule | TGAAACATGCAGGTATGGG | TCCTCGGAATTACCACATTTC |
| **Notch1** | Notch Receptor 1 | GTGCTCAGTGTGTCCTGTGAG | ACGTCAATGCCTCGCTTCTG |
| **Notch2** | Notch Receptor 2 | GTTACCTACCACAACGGCACA | CCAAGAAGCCCTCTGGACATC |
| **Hes1** | Hes Family BHLH Transcription Factor 1 | TAAGAAAGATAGCTCCCGGCATTC | CCAGAATGTCTGCCTTCTCTAGC |
| **Klf4** | Epithelial Zinc Finger Protein EZF | TCCTTTCCTGCCAGACCAGAT | CATGAGCTCTTGATAATGGAGAGAGG |
| **Krt18** | Keratin 18 | AGAATCATGAAGAGGAAGTCCAAGG | AGTCAATCCAGAGCTGGCAATC |
| **Nfe2l2** | Nrf2 | CAGCATGTTACGTGATGAGG | GCTCAGAAAAGGCTCCATCC |
| **Bach1** | BTB Domain And CNC Homolog 1 | TGTGGAGTTTCTAAGCGTACAC | GCTCTGAAGTGGAGTCCAAAAA |
| **Keap1** | Kelch Like ECH Associated Protein 1 | ACAGCAGCGTGGAGAGATATGA | GTCAACATTGGCGCGACTAGAT |
| **Gpx4** | Glutathione Peroxidase 4 | CATGCTGGGAAATGCCATCAAAT | ACCACGCAGCCGTTCTTATC |
| **Tgfb1** | Transforming Growth Factor Beta 1 | AGGTCACCCGCGTGCTAAT | TTTCTCATAGATGGCGTTGTTGC |
| **Smad2** | SMAD family member | CCAAGCACTTGCTCTGAAATTTGG | GTGTCCCACTGATCTACCGTATTTG |
| **Il6** | Interleukin 6 | GTAGCTATGGTACTCCAGAAGAC | ACGATGATGCACTTGCAGAA |
| **Egfr** | epithelial growth factor receptor | CTTCTTAAAGACCATCCAGGAG | TGAGGGCAATGAGGACATA |
| **Tnfa** | Tumor Necrosis Factor | GACCCTCACACTCAGATCATCTTCT | CCACTTGGTGGTTTGCTACGA |
| **Tnfrsf1a** | TNF Receptor Superfamily Member 1A | AGAAAGTGAGTGCGTCCCTTG | ATTTGCAAGCGGAGGAGGTAG |
| **Tnfrsf1b** | TNF Receptor Superfamily Member 1b | GTGCATGAGGCTGAGCAAGT | TTGCATAGCACATTTCCATTTGGG |
| **Myc** | MYC Proto-Oncogene, BHLH Transcription Factor | GCGACTCTGAAGAAGAGCAAGAA | CTTCTCCACAGACACCACATCAA |
| **Ccnd1** | Cyclin D1 | GCAGACCATCCGCAAGCAT | GAAATGAACTTCACATCTGTGGCA |
| **Rock1** | Rho Associated Coiled-Coil Containing Protein Kinase 1 | CAGACAGAGAAGTGAGATTGG | GAAGAGATGACGTTTGATTTCC |
| **Rhoa** | Transforming Protein RhoA | AAGCAGGAGCCGGTAAA | CCAATCCTGTTTGCCATATCT |
| **Akt1** | AKT Serine/Threonine Kinase 1 | CTGCTCAAGAAGGACCCTACAC | GCATGATCTCCTTGGCATCCT |
| **Bmp2** | bone morphogenetic protein 2 | ACGGACTGCGGTCTCCTAAA | GGGAAGCAGCAACACTAGAAGA |
| **Bmp4** | bone morphogenetic protein 4 | GAGCCAACACTGTGAGGAGTTT | CTGGGATGTTCTCCAGATGTTCTT |
| **Acvr1** | activin A receptor, type 1 | GAAGATGAGAAGCCCAAGGTCAA | GCCCTCACACACACACATGTAA |
| **Acvr2a** | activin receptor IIA | AAGCAAGGTTGTTGGCTGGA | ACAATCAGTCCTGTCATAGCAGTT |
| **Acvr2b** | activin receptor IIB | CTATTGCCCACAGGGACTTCAA | GGTCGCTCTTCAGCAGTACATT |
| **Bmpr1a** | bone morphogenetic protein receptor, type 1A | CAAAGCTCTGGGAGTGGATCTG | GGCAATAGTTCGCTGAACCAATAAA |
| **Bmpr2** | bone morphogenetic protein receptor, type II | TCCCAATGGATCTCTGTGCAAAT | ACCCAATCACTTGTGTGGAGAC |
| **Smad1** | SMAD family member 1 | CTACCACTATAAGCGAGTGGAGAG | GAAGGCTGTGCTGAGGATTGTA |
| **Rad51** | RAD51 Recombinase | GCTGCTTCAAGGTGGAAT | TCGGAATTCTCCAAACATCTC |
| **Cdkn1a** | Cyclin Dependent Kinase Inhibitor 1A | GACATTCAGAGCCACAGG | GAACAGGTCGGACATCAC |
| **Pten** | Phosphatase And Tensin Homolog | TGAGTTCCCTCAGCCATTGCCT | GAGGTTTCCTCTGGTCCTGGTA |
| **Acta2** | Actin Alpha 2 | TGACCCAGATTATGTTTGAGACCTT | GGGACAGCACAGCCTGAATAG |
| **Col1a1** | Collagen 1a1 | TGATGGACCTGCTGGCTCT | ACCACGTTGTCCAGCAATACC |
| **Col1a2** | Collagen 1a2 | TGCAATCGGGATCAGTACGAAAG | TCCACGTGGTCCTCTGTCTC |
| **Grem1** | Gremlin 1 | CCCACGGAAGTGACAGAATGAA | GCAACGCTCCCACAGTGTAT |
| **Vim** | Vimentin | CGAGAGAAATTGCAGGAGGAGAT | GTGCTTTCGGCTTCCTCTCTC |
