# Supplementary material for: Epithelial-mesenchymal interaction protects normal colonocytes from 4-HNE-induced phenotypic transformation
Source: PLoS One. 2024 Apr 26;19(4):e0302932. doi: 10.1371/journal.pone.0302932 (PMC11051638; doi:10.1371/journal.pone.0302932)
Supplement: S2 Table — (DOCX) [file pone.0302932.s014.docx]

| **Target** | **Clonality** | **Specy** | **Clone** | **Used dilution**  **Western blot** | **Used dilution**  **Immunofluorescence** |
| --- | --- | --- | --- | --- | --- |
| α-Smooth Muscle Actin | Monoclonal | Rabbit | 19245S  Thermofisher | 1 :1000 | 1 :100 |
| Cytokeratin 18 | Polyclonal | Rabbit | GTX105624  GeneTex | 1 :1000 |  |
| Cox2 | Monoclonal | Rabbit | 12282  Cel signaling Technology | 1 :1000 | 1 :400 |
| Rad51 | Polyclonal | Rabbit | GTX100469  GeneTex | 1 :500 |  |
| BMP4 | Polyclonal | Rabbit | SAB2700755 |  | 1 :300 |
| BMP2 | Monoclonal | Rabbit | Ab284387 | 1 :500 (dot blot) |  |
| P21 | Monoclonal | Mousse | GTX629543  GeneTex | 1 :500 |  |
| HSC70 | Monoclonal | Mouse | Sc-7298  Santa Cruz Biotechnology | 1 :1000 |  |
| Vinculin | Polyclonal | Rabbit | 26520  ProteinTech | 1 :5000 |  |
