# Supplementary material for: Epithelial-mesenchymal interaction protects normal colonocytes from 4-HNE-induced phenotypic transformation
Source: PLoS One. 2024 Apr 26;19(4):e0302932. doi: 10.1371/journal.pone.0302932 (PMC11051638; doi:10.1371/journal.pone.0302932)
Supplement: S3 Table — Western blot results were normalized and expressed as the mean ± SEM of at least 3 experiments. A two-way ANOVA was performed followed by Tukey’s multiple comparisons test. The same letters indicate no significant difference between the groups. qPCR data were normalized to the level of Hprt1 mRNA and analyzed via LinRegPCR v.11 software. Data are expressed as the mean ± SEM (n = 3 in triplicate). Two-way ANOVA was performed, followed by Tukey’s multiple comparisons test. The same letters indicate no significant difference between the groups. (DOCX) [file pone.0302932.s015.docx]

|  | Cell line | **D7 (western blot)**  **Mean ±SEM** | **Two way Anova** | **D21 (western blot)**  **Mean ±SEM** | **Two way Anova** |  | **D21 (rt-PCR)**  **Mean ±SEM** | **Two way Anova** |
| --- | --- | --- | --- | --- | --- | --- | --- | --- |
| **Krt18** | Co(m)-NT | 100.0 ± 4.3 | HNE treatment NS  Monoculture vs coculture NS  Interaction NS | 100.0 ± 4.6 | HNE treatment NS  Monoculture vs co-culture NS  **Interaction p=0.046** |  | 100.0 ± 2.4 ^b^ | HNE treatment NS  **Monoculture vs co-culture p=0.003**  Interaction NS |
|  | Co(m)-HNE | 130.7 ± 5.9 |  | 130.5 ± 11.5 |  |  | 104.9 ± 5.6^a,b^ |  |
|  | Co(c)-NT | 133.8 ± 15.7 |  | 149.2 ± 5.7 |  |  | 122.3 ± 5.1 ^a^ |  |
|  | Co(c)-HNE | 140.7 ± 9.0 |  | 125.2 ± 18.0 |  |  | 111.9 ± 4.5 ^a,b^ |  |
|  |  |  |  |  |  |  |  |  |
| **Vim** | nF(m)-NT | 100.0 ± 2.6 | HNE treatment NS  Monoculture vs coculture NS  Interaction NS | 100.0 ± 10.5 | HNE treatment NS  **Monoculture vs coculture p=0.043**  Interaction NS |  | 100.0 ± 4.4 | **HNE treatment p=0.010**  Monoculture vs co-culture NS  Interaction NS |
|  | nF(m)-HNE | 95.2 ± 11.8 |  | 86.2 ± 14.4 |  |  | 117.9 ± 9.26 |  |
|  | nF(c)-NT | 92.1 ± 8.4 |  | 66.6 ± 8.6 |  |  | 98.1 ± 2.5 |  |
|  | nF(c)-HNE | 87.9 ± 5.7 |  | 78.42 ± 8.4 |  |  | 109.9 ± 3.6 |  |
|  |  |  |  |  |  |  |  |  |
| **αSMA**  **(acta2)** | nF(m)-NT | 100.0 ± 6.6 | HNE treatment NS  Monoculture vs coculture NS  Interaction NS | 100.0 ± 10.4 ^a^ | HNE treatment NS  **Monoculture vs coculture p=0.001**  Interaction NS |  | 100.0 ± 4.5 ^a^ | **HNE treatment p=0.018**  Monoculture vs co-culture NS  Interaction NS |
|  | nF(m)-HNE | 75.1 ± 9.3 |  | 83.9 ± 7.3 ^a,b^ |  |  | 74.2 ± 4.3 ^a,b^ |  |
|  | nF(c)-NT | 86.0 ± 13.3 |  | 60.4 ± 4.9 ^b^ |  |  | 81.3 ± 3.5^a,b^ |  |
|  | nF(c)-HNE | 82.4 ± 12.1 |  | 70.6 ± 5.0 ^b^ |  |  | 69.7 ±13.1 ^b^ |  |
