# Supplementary material for: Epithelial-mesenchymal interaction protects normal colonocytes from 4-HNE-induced phenotypic transformation
Source: PLoS One. 2024 Apr 26;19(4):e0302932. doi: 10.1371/journal.pone.0302932 (PMC11051638; doi:10.1371/journal.pone.0302932)
Supplement: S4 Table — qPCR data were normalized to the level of Hprt1 mRNA and analyzed via LinRegPCR v.11 software. Data are expressed as the mean ± SEM (n = 3 in triplicate). A two-way ANOVA was performed followed by Tukey’s multiple comparisons test. Same letters indicate no significant difference between the groups. ne: not expressed. (DOCX) [file pone.0302932.s016.docx]

| Gene | Cell line | **D21**  **Mean ±SEM** | **Two way Anova** | Cell line | **D21**  **Mean ±SEM** | **Two way Anova** |
| --- | --- | --- | --- | --- | --- | --- |
| **TNFα** | Co(m)-NT | 100.0 ± 6.2 | HNE treatment NS  **Monoculture vs coculture p=0.032**  Interaction NS | nF(m)-NT | ne |  |
|  | Co(m)-HNE | 121.9 ± 15.9 |  | nF(m)-HNE | ne |  |
|  | Co(c)-NT | 144.7 ± 13.0 |  | nF(c)-NT | ne |  |
|  | Co(c)-HNE | 206.0 ± 51.0 |  | nF(c)-HNE | ne |  |
|  |  |  |  |  |  |  |
| **Tnfrsf1a** | Co(m)-NT | 100.0 ± 3.2 **^a,b^** | HNE treatment NS  Monoculture vs coculture NS  **Interaction p=0.039** | nF(m)-NT | 100.0 ± 3.4 **^b,c^** | HNE treatment NS  **Monoculture vs coculture p<0.001**  Interaction NS |
|  | Co(m)-HNE | 101.5 ± 4.0 **^a,b^** |  | nF(m)-HNE | 83.1 ± 4.7 **^c^** |  |
|  | Co(c)-NT | 111.3 ± 3.1 **^a^** |  | nF(c)-NT | 123.3 ± 8.9 **^a,b^** |  |
|  | Co(c)-HNE | 92.9 ± 6.4 **^b^** |  | nF(c)-HNE | 139.6 ±11.8 **^a^** |  |
|  |  |  |  |  |  |  |
| **Tnfrsf1b** | Co(m)-NT | 100.0 ± 2.6 **^a,b^** | **HNE treatment p<0.001**  Monoculture vs coculture NS  Interaction NS | nF(m)-NT | 100.0 ± 4.7 **^a,b^** | **HNE treatment p<0.001**  Monoculture vs coculture NS  Interaction NS |
|  | Co(m)-HNE | 87.5 ± 3.1 **^b,c^** |  | nF(m)-HNE | 70.2 ± 7.2 **^b^** |  |
|  | Co(c)-NT | 104.1 ± 2.4 **^a^** |  | nF(c)-NT | 114.9 ± 7.3 **^a^** |  |
|  | Co(c)-HNE | 78.2 ± 4.9 **^c^** |  | nF(c)-HNE | 81.8 ± 11.0 **^b^** |  |
