# Supplementary material for: Epithelial-mesenchymal interaction protects normal colonocytes from 4-HNE-induced phenotypic transformation
Source: PLoS One. 2024 Apr 26;19(4):e0302932. doi: 10.1371/journal.pone.0302932 (PMC11051638; doi:10.1371/journal.pone.0302932)
Supplement: S5 Table — qPCR data were normalized to the level of Hprt1 mRNA and analyzed using LinRegPCR v.11 software. Data are expressed as the mean ± SEM (n = 3 in triplicate). Two-way ANOVA was performed followed by Tukey’s multiple comparisons test. The same letters indicate no significant difference between the groups. (DOCX) [file pone.0302932.s017.docx]

|  | Cell line | **D21**  **Mean ±SEM** | **Two way Anova** | Cell line | **D21**  **Mean ±SEM** | **Two way Anova** |
| --- | --- | --- | --- | --- | --- | --- |
| **EGFR** | Co(m)-NT | 100.0 ± 4.3 | HNE treatment NS  Monoculture vs coculture NS  Interaction NS | nF(m)-NT | 100.0 ± 2.7 | **HNE treatment p=0.044**  Monoculture vs coculture NS  **Interaction p=0.040** |
|  | Co(m)-HNE | 102.2 ± 4.6 |  | nF(m)-HNE | 74.7 ± 6.0 |  |
|  | Co(c)-NT | 102.6 ± 10.2 |  | nF(c)-NT | 82.8 ± 6.3 |  |
|  | Co(c)-HNE | 99.5 ± 12.0 |  | nF(c)-HNE | 83.1 ± 7.1 |  |
|  |  |  |  |  |  |  |
| **IL6** | Co(m)-NT | 100.0 ± 3.4 | HNE treatment NS  Monoculture vs coculture NS  Interaction NS | nF(m)-NT | 100.0 ±3.5 ^b,c^ | HNE treatment NS  **Monoculture vs coculture p<0.001**  Interaction NS |
|  | Co(m)-HNE | 87.3 ± 6.6 |  | nF(m)-HNE | 71.1 ± 6.2 ^c^ |  |
|  | Co(c)-NT | 92.3 ± 7.2 |  | nF(c)-NT | 149.8 ± 15.6 ^a, b^ |  |
|  | Co(c)-HNE | 109.9 ± 14.0 |  | nF(c)-HNE | 165.0 ± 17.0^a^ |  |
|  |  |  |  |  |  |  |
| **Tgfb1** | Co(m)-NT | 100.0 ± 5.4 ^c^ | HNE treatment NS  **Monoculture vs coculture p<0.001**  Interaction NS | nF(m)-NT | 100.0 ± 3.7 | HNE treatment NS  Monoculture vs coculture NS  Interaction NS |
|  | Co(m)-HNE | 105.1 ± 4.5 ^b^**^, c^** |  | nF(m)-HNE | 108.1 ± 11.8 |  |
|  | Co(c)-NT | 133.1 ± 4.1 **^a^** |  | nF(c)-NT | 99.4 ± 9.9 |  |
|  | Co(c)-HNE | 120.2 ± 7.0 **^a, b^** |  | nF(c)-HNE | 100.8 ± 6.9 |  |
|  |  |  |  |  |  |  |
| **smad2** | Co(m)-NT | 100.0 ± 3.6 **^b^** | HNE treatment NS  **Monoculture vs coculture p<0.001**  Interaction NS | nF(m)-NT | 100.0 ± 3.2 | HNE treatment NS  Monoculture vs coculture NS  Interaction NS |
|  | Co(m)-HNE | 110.0 ± 5.7 **^a,b^** |  | nF(m)-HNE | 88.1 ± 4.5 |  |
|  | Co(c)-NT | 130.5 ± 4.0 **^a^** |  | nF(c)-NT | 99.6 ± 3.9 |  |
|  | Co(c)-HNE | 124.3 ± 8.5**^a^** |  | nF(c)-HNE | 102.9 ± 5.9 |  |
