# Supplementary material for: Epithelial-mesenchymal interaction protects normal colonocytes from 4-HNE-induced phenotypic transformation
Source: PLoS One. 2024 Apr 26;19(4):e0302932. doi: 10.1371/journal.pone.0302932 (PMC11051638; doi:10.1371/journal.pone.0302932)
Supplement: S1 File — (DOCX) [file pone.0302932.s018.docx]

**S1 Reference**

Khoury L, Zalko D, Audebert M. Validation of high-throughput genotoxicity assay screening using γH2AX in-cell western assay on HepG2 cells. Environmental and Molecular Mutagenesis 54, 737–746 (2013).
